# Assessing the Individual Interviewer Rapport-Building and Supportive Techniques of the R-NICHD Protocol

**DOI:** 10.3389/fpsyg.2021.659438

**Published:** 2021-07-21

**Authors:** Anett Tamm, Jana Otzipka, Renate Volbert

**Affiliations:** ^1^Psychologische Hochschule Berlin, Berlin, Germany; ^2^Institute of Forensic Psychiatry, Charité – Universitätsmedizin Berlin, Corporate Member of Freie Universität Berlin and Humboldt-Universität zu Berlin, Berlin, Germany

**Keywords:** R-NICHD protocol, child sexual abuse, rapport, support, interviewing

## Abstract

The use of the rapport-building and supportive techniques formulated by the R-NICHD protocol is intended to support children and increase the quality of their statements as well as disclosures without possessing suggestive potential. While the effectiveness of the entire R-NICHD protocol for children who have actually experienced child sexual abuse (CSA) has been supported by research, to date no study assessed the effect of each individual socio-emotional interview technique in both interviewees with and without CSA experiences. The current study aimed to address this gap in research by means of an online vignette-study, asking participants to rate the identified rapport-building and supportive techniques on the scales well-being, willingness to talk, and perceived pressure. A total of 187 participants were randomly assigned to either a hypothetical “abused” or a hypothetical “not abused” group by means of a vignette-manipulation. The results suggest that many socio-emotional interview techniques were perceived as supportive and non-suggestive, while a number of techniques were perceived as not supportive but suggestive. Few differences emerged between the hypothetical “abused” group and the hypothetical “not abused” control group. To conclude, most but not all rapport-building and supportive techniques proposed by the R-NICHD protocol had a positive effect on interviewees.

## Introduction

Once the suspicion of child sexual abuse (CSA) arises, it is pivotal to conduct a proper and adequate interview. To achieve this goal, several detailed interview protocols and interviewing guidelines have been drafted, reflecting approximately three decades of intensive research on children’s memory and suggestibility (Memon et al., 2010; Lyon, 2014; Saywitz and Camparo, 2014; Cirlugea and O’Donohue, 2016; Niehaus et al., 2017; Lamb et al., 2018). Although details differ, consensus exists regarding the central aim of all interview protocols: to enable children to give the most accurate and complete account of the event in question by interviewing them in a non-suggestive way and using open prompts. When children are willing to provide information and are interviewed in accordance with the recommended protocols, they are often able to provide reliable statements (Brubacher et al., 2019). Thus, the reason interview protocols were developed in the first place was to help interviewers to avoid poor questioning strategies that may lead to contamination or memory distortions.

On the other hand, research on disclosure supports the overall notion that many children do not disclose abuse on their own initiative (Lyon, 2007; London et al., 2008; McGuire and London, 2020). In addition, 7–26% of children who had previously disclosed abusive experiences did not make a disclosure in subsequent forensic interviews (e.g., Hershkowitz et al., 2005, 2006). This might be due to a number of barriers such as perceived negative consequences, feelings of self-blame, shame, and guilt or avoidance of an upsetting topic (Lemaigre et al., 2017). Investigative interviews in cases of CSA demand a high level of openness from children, which is rare in their typical interactions with strangers. Therefore, it is not surprising that many children are anxious, often have concerns about the consequences of their reports and are reluctant to disclose abuse (Saywitz et al., 2015). Moreover, Hershkowitz et al. (2006) found that investigative interviewers responded to children’s initial uncooperativeness with increasingly less supportive rather than more supportive comments. Predictably, this resulted in a further increase in children’s resistance.

Interview guidelines should therefore not only solely focus on memory and suggestibility outcomes, but also take into account that unfamiliar interviewers must gain trust and cooperation from children. Hence, it is considered important to build rapport with the interviewee and to support him or her in this difficult interview-situation (Pipe et al., 2007; Hershkowitz et al., 2015). Rapport can be defined as the establishment of interpersonal trust between the interviewer and the interviewee, while communicating respect, understanding, and acceptance (Hershkowitz et al., 2014). The use of rapport-building is thought to enhance cooperation, to help overcome anxiety and resistance and to make the process of talking to strangers about potentially aversive, fear- and shame-inducing topics easier (Saywitz et al., 2015). Interviewer support is defined as “a form of social interaction or communication that fosters a feeling of well-being in the target” (Davis and Bottoms, 2002, p. 186). In sum, socio-emotional interview techniques are supposed to promote adaptive coping, while having a calming effect on the interviewees, which allows them to effectively use their cognitive resources without being distracted by negative emotions and thoughts (Blasbalg et al., 2018).

Different needs may exist for different age groups in terms of the kind and amount of support required. Preschoolers, who may not have understood the sexual character of an abuse, might benefit especially from making them feel comfortable, while talking to a stranger. School-aged children on the other hand may be more aware of the sexual nature of the experienced abuse and may therefore feel ashamed or guilty which has to be overcome (Hershkowitz, 2009). Additionally, they may also be more aware of the possible consequences of their allegations (cf. Niehaus et al., 2017). Moreover, practitioners interviewed in a study by Collins et al. (2014) reported that adolescents were less willing than children to engage in an elaborate rapport phase and attempts at this could reduce their responsiveness. They warned against patronizing adolescents when trying to establish a relationship and suggested a shorter and less structured format for this age group (Collins et al., 2014).

Given that a forensic interview situation can induce anxiety or stress *per se* and many children are at least somewhat nervous when being interviewed by a stranger about a potentially aversive event, socio-emotional supportive elements are included in nearly all forensic interview guidelines (Memon et al., 2010; Lyon, 2014; Saywitz and Camparo, 2014; Faller, 2015; Niehaus et al., 2017; Lamb et al., 2018). One of these interview protocols, the NICHD protocol, now includes a revision that specifically addresses this issue (Hershkowitz et al., 2014).

The NICHD protocol is a structured but flexible interview protocol, which aims to guide the interviewer and attempts to elicit as much high-quality information from the child witness as possible (Lamb et al., 2007).^1^ The use of the NICHD protocol has been widely supported by research and showed superior performance, both in the sense of quantity and quality of information provided by the interviewee as well as the quality of the questions asked by the interviewer, compared to interviews in which no interviewing protocol was used (Lamb et al., 2007, 2009; La Rooy et al., 2015). The NICHD protocol has been translated into many languages, has directly influenced national or regional procedures in several countries (La Rooy et al., 2015) and is probably the most widely used interview protocol in interviews with children (Cirluega and O’Donohue, 2016).

While the NICHD protocol was initially developed to improve statements provided by children who are generally willing to disclose (Lamb et al., 2008), the development of the R-NICHD protocol led to a shift in focus on additionally creating a willingness to disclose in reluctant children. Compared to the standard version of the NICHD, the revised version focuses even more on building rapport with the interviewee as well as to support him or her in possibly difficult interview-situations by means of applying supportive techniques and utterances from six different categories: (A) Addressing the Child in a Personal Way, (B) Establishing Rapport, (C) Reinforcement, (D) Using Rapport, (E) Emotional Support, and (F) Encouragement (Hershkowitz et al., 2015; Lamb et al., 2018). The R-NICHD protocol does not only contain a rapport-building phase at the very beginning of the interview, i.e., the pre-substantive phase, but also provides a number of techniques throughout the substantive phase of the interview, shifting the focus of the interview from cognitive factors toward socio-emotional aspects (Hershkowitz et al., 2014). Several studies have confirmed the superior position of the R-NICHD compared to the standard NICHD protocol when considering disclosure rates as well as interviewer support and appropriate questioning (e.g., Ahern et al., 2014, 2019; Hershkowitz et al., 2014, 2015, 2017; Blasbalg et al., 2018, 2019; Hershkowitz and Lamb, 2020). It therefore appears that rapport-building, which is sustained throughout the interview, and supportive interviewer behavior are most effective to elicit new forensically relevant information from children concerning child (sexual) abuse.

However, it is crucial that a child who remains silent because he or she has not experienced abuse is not mistaken for a child who remains silent because he or she feels reluctant to disclose an abuse. In situations in which a child has not made any or only vague allegations it is of utmost importance to avoid rapport-building and supportive techniques that possess a suggestive potential, as certain types of support and reinforcement were shown to cause false allegations and inaccurate information to be provided by child witnesses. Suggestive reinforcement may not only cause children to make false allegations (Garven et al., 2000), but also caused them to falsely incriminate themselves in the theft of a toy (Billings et al., 2007).

The authors of the R-NICHD protocol state that the socio-emotional interview techniques included in the protocol are non-suggestive (e.g., Hershkowitz et al., 2014). In order to support this statement, they cite a meta-analysis by Saywitz et al. (2019) which showed that non-suggestive, non-contingent support improves the accuracy of children’s statements instead of impairing it (Hershkowitz et al., 2017; Blasbalg et al., 2018; Ahern et al., 2019). However, this meta-analysis included studies on various different rapport-building and supportive techniques, in many instances focusing on non-verbal techniques, and did not exclusively focus on the verbal rapport-building and supportive techniques put forth by the R-NICHD protocol. Beneficial effects were found when interviewers used a warm, friendly as well as positive approach, administered supportive behaviors in ways that were not contingent on the content of children’s responses, and avoided nonsupportive behaviors such as being formal and distant, making no attempt to develop rapport and demonstrating instances of frustration or criticism (Saywitz et al., 2019).

Furthermore, to the best of our knowledge, no single study on socio-emotional support so far nor any study supporting the effectiveness of the R-NICHD protocol contained a control group of children who had not experienced the target event (e.g., Ahern et al., 2014, 2019; Hershkowitz et al., 2014, 2015, 2017; Blasbalg et al., 2018, 2019; Saywitz et al., 2019; Hershkowitz and Lamb, 2020). All of the children’s allegations of child (sexual) abuse in the field studies assessing the R-NICHD protocol were externally validated by additional evidence such as previous disclosures or eyewitnesses and hence considered “true” child (sexual) abuse cases. Therefore, it is not known what effect the techniques proposed by the R-NICHD protocol have on children who have not experienced (sexual) abuse. Additionally, while studies did consistently show positive results for the rapport-building and supportive techniques in terms of disclosure rates for children who did experience CSA, it has to be noted that some of these studies counted every new/unknown forensically relevant detail the child disclosed in the interview as a new accurate detail, without knowing whether this specific detail was really accurate (Hershkowitz et al., 2015; Blasbalg et al., 2018, 2019). As it was shown that suggestive rapport-building and support can lead to inaccurate information being disclosed, it is important to resolve the question whether the socio-emotional interview techniques prescribed in the R-NICHD protocol are consistently supportive without jeopardizing the accuracy of the information. It should be ensured that none of the techniques increase children’s suggestibility due to a desire to please the interviewer and avoid adult disappointment or rejection, especially in the case of children who have not experienced CSA. When examining the techniques prescribed by the R-NICHD protocol more closely, some of them might appear, at least at face-value, to communicate a certain expectation of what the interviewer wants to hear from the child (e.g., “Child’s name, I really want to know when something happens to children. That’s what I am here for” or “It’s really important that you tell me if something is happening to you,” Lamb et al., 2018).

Previous studies have focused on the effectiveness of the entire R-NICHD protocol compared to no protocol or the standard NICHD protocol. Therefore, the effect of the individual rapport-building techniques is unknown. Thus, while the entire protocol as a whole has shown good results, it is unclear if this is due to the sum of techniques or a few good and effective techniques and whether some individual techniques could actually have a negative effect on the interviewees because of their suggestive potential. While focusing on the effectiveness of the individual techniques it has to be taken into account that certain techniques are only to be used in certain phases of the interview, for example, when the child expresses reluctance or avoidance and/or when independent evidence for the suspicion of CSA exists. However, in the protocol it is not specified what this independent evidence should entail.

Considering this background, the current study aims to shed light on the effects of each individual rapport-building and supportive technique recommended by the R-NICHD protocol. An online-study was developed, and participants were randomly assigned to either an experimental (hypothetically “abused”) group or a control (hypothetically “not abused”) group by means of a vignette-manipulation. They were then asked to rate the socio-emotional interview techniques formulated by the R-NICHD protocol. The groups were analyzed separately, but no differences between groups were expected in terms of the rating of the individual rapport-building techniques. Furthermore, it was expected that, contrary to statements made by the authors of the R-NICHD protocol, some techniques from the protocol would be perceived as pressuring, while reducing well-being and willingness to talk. However, no specific hypotheses were formulated per technique as the study was of an exploratory nature. This exploratory study aims to provide a first indication on how supportive and/or suggestive the individual rapport-building and supportive techniques prescribed by the R-NICHD protocol are perceived by both hypothetically sexually abused and hypothetically not abused interviewees.

## Materials and Methods

### Overview

This study was conducted by means of an online survey-platform involving a vignette-manipulation. The survey was created *via* SoSci Survey (Leiner, 2019) and made available to participants at www.soscisurvey.de. Ethical approval was obtained from the Ethical Committee of the Psychologische Hochschule Berlin. The survey was conducted with adult participants. For more information related to the participants and the reasoning behind this choice, refer to section “Participants and Recruitment.”

### Design

Participants were randomly assigned to one of two experimental groups, an “abused” group and a “not abused” group, by means of a vignette-manipulation (for more information on the vignettes refer to the vignettes section in “Materials and Procedure”). Thus, the hypothetical status of “abused” and “not abused” served as levels of the independent variable.

The dependent variables consisted first and foremost of the ratings of the selected rapport-building and supportive techniques. A good socio-emotional interview technique should make the interviewee feel supported, while not possessing any suggestive potential (Hershkowitz et al., 2014). As the lay participants were expected to lack the background knowledge pertaining to the constructs of suggestiveness und supportiveness, it was decided to instruct them to rate an operationalized version of these constructs in order to avoid misunderstandings. Therefore, the rating scales were reformulated into “willingness to talk,” “well-being” as well as “perceived pressure to adhere to the interviewer’s expectations.” The first two scales represent the concept of supportiveness as supportive techniques should put interviewees at ease, while at the same time increasing disclosure rates. The latter scale represents the concept of suggestiveness.

In summary, participants rated each technique in terms of how it changed their well-being, willingness to talk as well as the perceived pressure. In order to be able to compare the two groups with presumably different initial well-being and willingness to talk, participants rated the influence of each technique in relation to their initial level (and not as an absolute measure), which had to be indicated at the start of the survey.

### Participants and Recruitment

In order to answer the research questions, undergraduate students from universities throughout Germany as well as other lay people were recruited *via* social media and university-specific recruitment websites and systems. Students from the Freie Universität Berlin as well as the Psychologische Hochschule Berlin were rewarded with one research-participation-hour for their participation. Based on sample calculations by means of G*Power (Faul et al., 2009; input parameters: effect size = 0.4, adjusted α error probability = 0.012, power = 0.90), it was determined that a minimum of 94 participants per group is required in order to ensure sufficient power.

Due to ethical and practicality issues, it was decided to use an adult-participant sample instead of a child-participant sample, even though this would not allow us to draw conclusions on developmental aspects of support and rapport-building. The study addressed a distressing topic (CSA) and required advanced introspection skills due to the vignette-manipulation, thereby excluding the possibility to use child-participants. Furthermore, instead of instructing adult participants to imagine that they were in the position of a child being interviewed about the possibility of an abuse, participants were instructed to rate the techniques from their adult perspective. This was decided in order to avoid confounding through false or biased preconceptions participants may hold when it comes to what they think would be supportive or manipulative when interviewing children. It is deemed too difficult for participants to completely place themselves into the position of a child being interviewed without any influence of myths and preconceptions they hold as adults pertaining to this topic.

In total, 343 participants responded but only 197 participants completed the survey until its final page. The remaining 146 participants who prematurely ended their participation were excluded. Of the 197 who completed the survey, 10 additional participants had to be excluded, as they either did not fill in any questions and just clicked through until the last page of the survey (two participants) or they did not read the vignette properly and therefore failed on one or more of the manipulation-check multiple-choice-questions (eight participants). After the exclusion, 96 participants remained in the hypothetical “abused” group, while 91 participants remained in the hypothetical “not abused” group, making up a total of 187 participants.

The age of the participants ranged from 18 to 78 years (*M* = 27.44, *SD* = 10.52) with the median age being 23 years. Around 153 participants (82%) were female, while 32 participants (17%) were male. One participant indicated to identify as Other and one participant did not specify his or her gender (less than 1% respectively). Almost two-thirds of the sample had a background in psychology (65%), either as students or as practitioners. Moreover, one person indicated to have a background in legal psychology (less than 1%) and 3% of the sample worked as psychotherapists. About 7% of the sample indicated to have a background in social work or social pedagogy, 4% of the sample were currently in teacher training and another 7% of the sample stated that they hold a position in the police force or currently attend a police college. The remainder of the sample indicated to have a background in various different fields. Furthermore, 65% of the samples were Bachelor’s students, 11% were Master’s students, 3% currently partook in an educational job apprenticeship, and 16% indicated to be working professionals. Lastly, 19% of the sample stated to have experienced some form of sexual violence in the past, 77% indicated to not have experienced sexual violence and 4% chose to not provide an answer to this question.

### Materials and Procedure

#### Informed Consent

On the first page of the survey, participants were given background information on the study, its aim and design, the procedure, possible risks involved with their participation and contact information of the responsible researchers. It was ensured that participants were clearly informed that this study would address the topic of CSA. Thereby, participants who did not wish to be exposed to this topic had the opportunity to withdraw from participating before being confronted with the vignettes. Furthermore, on each page of the survey, contact information of organizations supporting sexual abuse/violence survivors were provided, in case any participant was distressed by the content of the survey or felt the need to talk to someone during and/or after his or her participation. After obtaining informed consent, participants were asked to provide information on their demographics, including their age, gender, and field of study or profession. In the case of students, their semester of study was obtained.

#### The Vignettes

Next, the online survey automatically and randomly assigned each participant by means of a vignette-manipulation to one of two experimental groups, a hypothetical “abused” group as well as a hypothetical “not abused” group (Appendix A). Both groups were instructed to imagine that they were surprisingly contacted by the police, as their former primary school teacher was suspected of having sexually abused several children. Next, the “abused” group was told to imagine that they indeed had been sexually abused by this teacher as a child; however, they had never shared this information with anyone and were reluctant to do so during the interview with the police as they simply wanted to forget about the abuse. On the other hand, the “not abused” control group was instructed to imagine that they never had been sexually abused by the teacher and remembered him as a caring person.

It was opted for the use of vignettes instead of a free imagination task as vignettes allow, at least to a certain extent, to exert control on what participants are imagining. Furthermore, vignettes offer the possibility to create a control group that is, besides from the manipulation (hypothetical sexual abuse occurred = yes/no), almost identical to the experimental group. In order to ensure that participants actually read their respective vignette, two straightforward multiple-choice questions were asked pertaining to the content of the vignette. Before analyzing the data, a manipulation check was conducted and participants who failed to answer one or more of these control-questions correctly were excluded from the analyses. Due to ethical considerations, it was decided to use a vignette-scenario involving sexual abuse by a primary school teacher instead of an intra-familial sexual abuse scenario. The selected scenario is considered easier to imagine for participants, while still involving a trusted caregiver perpetrating the sexual abuse.

#### The Rapport-Building and Supportive Techniques

In total, 26 socio-emotional interview techniques belonging to six overarching categories of rapport, namely establishing rapport, reinforcement, using rapport, emotional support, kind encouragements and addressing the child by name, were identified in the R-NICHD protocol (Lamb et al., 2018, p. 166, Table 9.1). For each of the 26 techniques, several examples were provided by the R-NICHD protocol and its appendices (retrieved from the official website http://nichdprotocol.com/Deutsch.pdf and http://nichdprotocol.com/Deutsch2.pdf, respectively). As previously mentioned, some of these examples ought to be used only in specific situations or phases of the interview. For example, in some cases examples are linked to the degree of reluctance displayed and/or expressed by the child as well as the presence of independent evidence arousing suspicion. In this first approach to examining the individual techniques, it was decided to ask participants to rate all identified examples of the rapport-building and supportive techniques, as contextual conditions and the developmental aspects addressed in the introduction cannot be easily replicated in a vignette study. However, some techniques were excluded as they appeared too similar. This resulted in 14 examples being excluded (Appendix C).

Furthermore, since participants were recruited throughout Germany, the official translated German version of the R-NICHD protocol was used to extract the examples for each technique (Noeker and Franke, 2018). The wording of the techniques was adapted to adult recipients.

In sum, a total of 67 examples of socio-emotional interview techniques from the R-NICHD protocol were included and rated in the survey. In addition, alternative translations were supplied for four examples in order to facilitate a comparison with the original translation. The results pertaining to the alternative translations are beyond the scope of this paper. For a list of the included techniques along with their item codes, refer to Appendix B.

#### The Rating

Before participants proceeded to rate the rapport-building and supportive techniques, a detailed explanation of each of the three rating-scales was provided along with an example question, representing the format in which the techniques were going to be rated. It was explained that some of the presented techniques could increase well-being, while others could create a feeling of discomfort. Then again, some techniques could encourage them to talk about what they had experienced pertaining to the teacher, while other techniques might cause them to feel like they did not want to share any more information with the police officer, for various reasons. Lastly, some techniques could potentially communicate a certain expectation of what the police officer seemed to want to hear, while other techniques might make them feel like they were completely free to talk about whatever they had experienced. The three scales were rated on seven-point Likert-scales: very decreased (=−3), moderately decreased (=−2), slightly decreased (=−1), neutral (=0), slightly increased (=1), moderately increased (=2), and very increased (=3). Furthermore, participants were informed that they would have the opportunity to comment on each of the techniques presented to them and they were encouraged to make use of this option in order for the authors to better understand their ratings.

After reading these instructions, the 67 rapport-building and supportive examples were presented to the participants for rating in a randomized order.

#### Other Variables

As previously mentioned, at the beginning of the survey participants were asked to indicate how they would rate their current state in terms of well-being as well as willingness to talk (on a slider-bar ranging from 1 to 101 with 1 being very low and 101 being very high) in order to specify a reference point. This measurement provided another manipulation-check variable for the effectiveness of the vignette-manipulation. If the vignette-manipulation worked as intended, participants in the “abused” group should initially indicate a lower state of well-being as well as willingness to talk than the “not abused” group.

Additionally, on the last page of the survey participants were again asked to rate their current state in terms of well-being as well as willingness to talk. Post-survey well-being and willingness to talk provided an impression of the overall effect of the survey, thus the sum of techniques. It was decided not to ask participants to rate pre- and post-survey perceived pressure as this concept is thought to only refer to a specific question or statement posed by the interviewer.

Furthermore, after completion of the survey participants were asked to indicate how distressing the experience of partaking in the study was for them. This variable also allowed to control for the intended effect of the survey, with participants in the “abused” group being expected to report a higher level of distress caused by the survey compared to participants in the “not abused” group.

Lastly, participants were asked to indicate whether they had ever experienced any form of sexual violence before (levels: “yes,” “no,” and “no response”). It was not expected that this variable would have an impact on the ratings of the socio-emotional interview techniques. Nonetheless, it was deemed important to include this question in order to control for this variable in later analyses. Due to the small expected sample size of participants with own sexual violence experiences, analyses focused on differences in the pre- and post-survey measurements of well-being and willingness to talk as well as the indicated amount of distress caused by the survey.

## Results

### Pre- and Post-survey Well-Being and Willingness to Talk

As previously discussed, participants were asked to specify their well-being as well as willingness to talk both in the beginning and at end of the survey in order to control for the effectiveness of the vignette-manipulation, as well as to obtain an indication of the cumulative effect of all techniques. Assuming that the vignette-manipulation was effective, it was expected that the well-being and willingness to talk at the onset of the survey, thus right after having read the vignette, would be significantly lower in the “abused” group compared to the “not abused” group. Furthermore, it was expected that there would be a significant increase in both reported well-being and willingness to talk, irrespective of group, from pre- to post-survey measurements. No interaction effect of survey and group was expected.

#### Well-Being

A mixed factorial ANOVA, with survey as the within-subjects factor (pre- and post-survey) and group as the between-subjects factor, was conducted to test these hypotheses for reported well-being.

**Figure 1:**
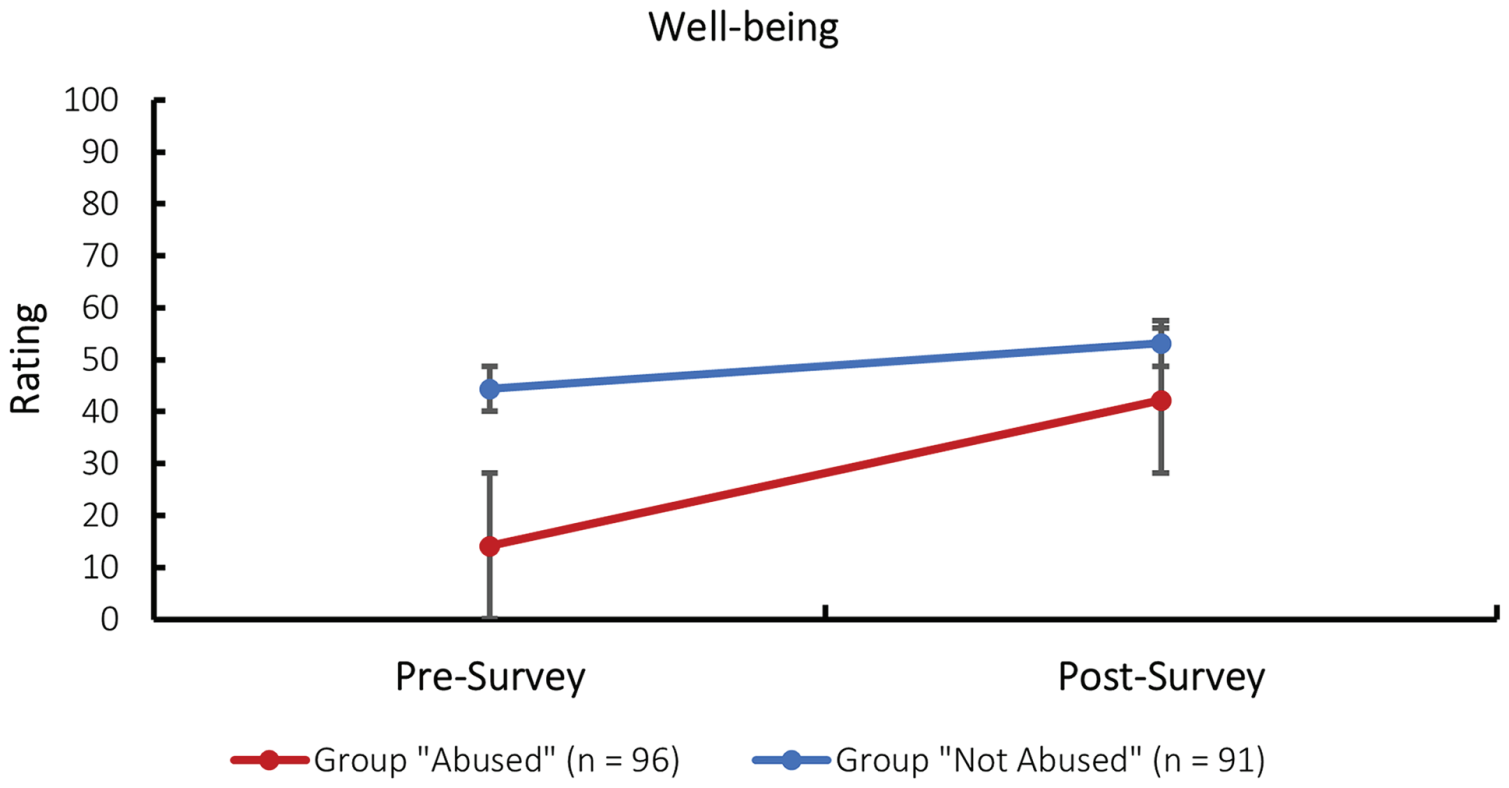
Change from pre- to post-survey well-being per experimental group. Error bars show SEs.

The test for between-subjects effects yielded a significant main effect for group *F*(1, 185) = 71.30, *p* < 0.001, partial *η*^2^ = 0.28. Upon examining the output it appeared that participants in the “not abused” group (*M*_PRE_ = 44.37, *SD*_PRE_ = 25.66; *M*_POST_ = 53.09, *SD*_POST_ = 21.07) rated their well-being as higher than participants in the “abused” group (*M*_PRE_ = 14.10, *SD*_PRE_ = 14.49; *M*_POST_ = 42.11, *SD*_POST_ = 19.43) at all times. This result is in line with the previously postulated hypotheses. The tests of within-subjects effects yielded a significant result for both the main effect of the survey (pre- and post-survey), *F*(1, 185) = 112.17, *p* < 0.001, partial *η*^2^ = 0.38, as well as the interaction between the survey and experimental group, *F*(1, 185) = 30.97, *p* < 0.001, partial *η*^2^ = 0.14. In other words, while the survey including all its rapport-building and supportive techniques increased the participants well-being significantly across both groups, it had a stronger effect in the “abused” group, who started off with a significantly lower well-being, compared to the “not abused” group (see Figure 1). The fact that the “abused” group indicated a significantly lower well-being at the beginning of the survey confirmed the effectiveness of the vignette-manipulation.

#### Willingness to Talk

A mixed factorial ANOVA, with survey as the within-subjects factor (pre- and post-survey) and group as the between-subjects factor, was next conducted for the measurements of reported willingness to talk.

**Figure 2:**
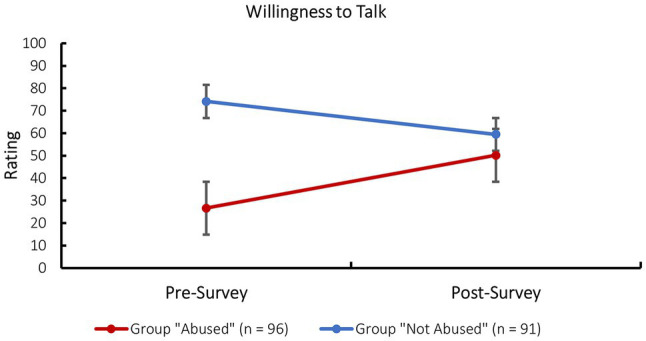
Change from pre- to post-survey willingness to talk per experimental group. Error bars show SEs.

The test for between-subjects effects yielded a significant main effect for group, *F*(1, 185) = 94.42, *p* < 0.001, partial *η*^2^ = 0.34. Participants in the “not abused” group (*M*_PRE_ = 74.13, *SD*_PRE_ = 21.74; *M*_POST_ = 59.46, *SD*_POST_ = 22.24) reported their willingness to talk to be higher compared to the “abused group” (*M*_PRE_ = 26.60, *SD*_PRE_ = 26.85; *M*_POST_ = 50.16, *SD*_POST_ = 23.29) at all times. The tests of within-subjects effects on the other hand yielded a significant result for both the main effect of the survey (pre- and post-survey), *F*(1, 185) = 5.75, *p* = 0.017, partial *η*^2^ = 0.03, as well as the interaction between the survey and experimental group, *F*(1, 185) = 106.54, *p* < 0.001, partial *η*^2^ = 0.37. Upon closer inspection of the results, it appeared that while the accumulative effect of all socio-emotional interview techniques in the survey significantly increased the willingness to talk for the “abused” group, it significantly decreased the willingness to talk for the “not abused” group (see Figure 2). The fact that the “abused” group indicated a significantly lower willingness to talk at the beginning of the survey again confirmed the effectiveness of the vignette-manipulation.

### Rating of the Individual Techniques

The main objective of our study was to gain insight into the influence of each individual rapport-building and supportive technique postulated by the R-NICHD protocol on well-being, willingness to talk, and perceived pressure. The influence was operationalized as the deviation of the ratings of each variable from neutral zero. Two-sided one-sample *t*-tests were conducted for each item of the survey. Significant deviations were interpreted, depending on their valence, as a significant enhancement or reduction in well-being and willingness to talk, or as perceived pressure to agree with the statements of the interviewer as opposed to the perceived emphasis on being able to convey one’s own point of view. An average rating not significantly differing from 0 would translate to the item having no effect on the participants.

The data analysis was split between the two experimental groups and the means for each item on the three scales were calculated per group. Even though no differences between groups were expected, this split would allow for possible differences to become visible. Additionally, in order to better understand the results, the comments provided by participants on each respective example were consulted. A full and extensive documentation of all comments provided by participants in this study can be found in Appendix G.

#### Group “Abused”

As multiple hypothesis testing leads to an inflated risk for a Type I error, the Benjamini-Hochberg procedure was applied. The Benjamini-Hochberg procedure controls the false discovery rate (FDR) at a set percentage, which is selected *a priori* by the researcher (Benjamini and Hochberg, 1995). For this study, an FDR of 5% was chosen. This means that 95% of the significant results discovered by this analysis can be assumed to be truly significant results. Although it was expected that the majority of the items would increase well-being and willingness to talk, while having no or even a decreasing effect on perceived pressure, as suggested by the authors of the R-NICHD protocol, it was also anticipated that some items would have the opposite effect. However, no specific hypotheses were formulated per item as this analysis was exploratory in nature. For this reason, it was decided not to apply the Bonferroni correction, as this method is very conservative and can diminish the statistical power of the tests, thereby inflating the number of type II errors (Benjamini and Hochberg, 1995; Narum, 2006).

The one-sample *t*-tests applying the Benjamini-Hochberg procedure on the scale measuring *well-being* revealed significant results for 54 of the 67 items representing the rapport-building and supportive techniques; 13 items did not receive a rating which significantly differed from 0 and can therefore be assumed to have no effect on this scale. Of the 54 techniques yielding a significant result, 10 significantly differed from 0 in the negative direction (Cohen’s *d* ranging between −0.22 and −2.42), indicating that they significantly decreased the participants well-being instead of increasing it. In turn, 44 techniques significantly increased well-being (Cohen’s *d* ranging between 0.22 and 1.94).

Next, the same procedure as described previously was applied to the ratings of the scale *willingness to talk*; 51 of the 67 items significantly differed from 0, while 16 of the items did not and can therefore be assumed to have no effect on the participants’ willingness to talk. Of the 55 items significantly differing from 0, four differed in the negative direction thereby significantly decreasing the participants’ willingness to talk (Cohen’s *d* ranging between −0.40 and −1.69). The other 47 items induced the inverse effect (Cohen’s *d* ranging between 0.21 and 1.43).

Finally, the same procedure was applied to the ratings of *perceived pressure*. The tests found significant results for 52 of the 67 techniques; 15 of the items received ratings that did not significantly differ from 0. Among the 52 items that received ratings, which significantly differed from 0, 26 items significantly differed from 0 in the positive direction (Cohen’s *d* ranging between 0.25 and 1.37). In other words, 26 items representing rapport-building and supportive examples drawn from the R-NICHD protocol significantly increased the perception of pressure to adhere to the interviewer’s expectation for participants in the “abused” group. On the other hand, another 26 of the items significantly differed from 0 in the negative direction (Cohen’s *d* ranging between −0.23 and −1.24), which means that participants felt free to share their story on their terms in response to these statements and questions (for the results per *t*-test refer to Appendix D).

**Figure 3:**
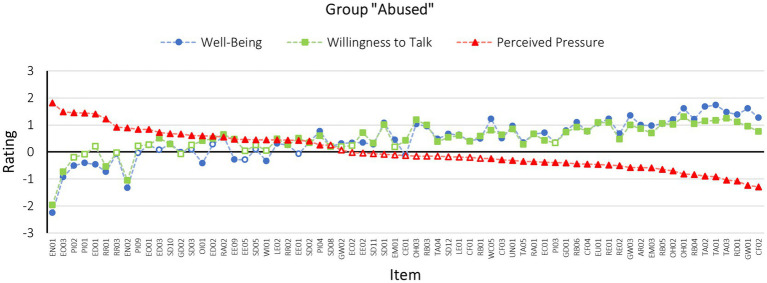
Scale means for each R-NICHD technique example as rated by the group “abused” (*n* = 96). The items are sorted based on their rating on the scale perceived pressure. Filled dots refer to significant differences from 0 based on the results of one-sample *t*-tests corrected by means of the Benjamini-Hochberg procedure, while blank dots refer to non-significant differences from 0. For an explanation of the item codes, please refer to Appendix B.

Figure 3 depicts participants’ average ratings per item on each scale within the “abused” group. The items are sorted based on their rating on the scale perceived pressure. Table 1 (above the diagonal) shows that the ratings on the three scales are highly correlated with one another. In other words, when an item increased the perceived pressure exerted by the interviewer it was simultaneously rated as reducing both participants’ well-being and willingness to talk. On the other hand, items rated as increasing participants’ well-being and willingness to talk were found to additionally reduce the perceived pressure exerted by the interviewer.

**Table 1:** Intercorrelations of participants’ average R-NICHD technique ratings on the scales well-being, willingness to talk, and perceived pressure for both groups.

	Average well-being	Average willingness to talk	Average perceived pressure
Average well-being	–	0.93^**^	−0.88^**^
Average willingness to talk	0.94^**^	–	−0.80^**^
Average perceived pressure	−0.88^**^	−0.81^**^	–

The results for the average ratings in the group “abused” (*n* = 67) are shown above the diagonal. The results for the average ratings in the group “not abused” (*n* = 67) are shown below the diagonal.

** *p* < 0.001, two-tailed.

Next, participants’ comments were considered. Items sorted on the right end of Figure 3 involve statements rated as reducing the perceived pressure communicated by the interviewer, while significantly increasing participants’ well-being and willingness to talk. These items were described by participants as empathetic, respectful, and communicating that the interviewee is taken seriously (e.g., “empathetic” [GW01 – Are you cold? Would you like a short break?], “Appreciation, being seen, being taken seriously” [TA02 – I really appreciate that you have spoken to me], “I feel valued” [TA03 – Thanks for trying hard to remember and tell me what happened. Thank you for sharing with me], “I’m staying self-determined” [RD01 – It’s your choice whether to tell me or not, and it is my job to let you choose/and I will go with your choice], “Sometimes it is difficult to put bad experiences into words, I feel understood” [RB06 – I can see what you are saying], “I feel treated with respect” [WC05 – I am glad to meet you today/to get to know you/to get to talk to you. My name is…] and “I feel taken seriously and not as if words are being put into my mouth” [RB05 – You corrected me and that is important]).

Items sorted on the left side of Figure 3 are items, which were rated to significantly increase the perceived pressure by the interviewer, while reducing well-being and in some cases the willingness to talk. Comments pertaining to these items communicated that participants were experiencing the techniques as manipulative, pressuring as well as unprofessional (e.g., “looks very personal, not professional” [PI01 – I really want to get to know about you. Today is the first time we have met and it is important for me to know you better], “Feeling: Person only wants to get to know me in order to get my information and is not really interested in me” [RR01 – You have told me a lot about yourself and I feel I know you better. Now that we know each other better you can share with me] and “The officer expects me to tell him about an incident. He’s pushing me for a statement” [EO03 – I’m sure you could tell me]). Some items were moreover described as inappropriate, discomforting and in several cases perceived as communicating distrust toward the interviewee (e.g., “I find it very inappropriate to sit next to him” [EN02 – *Name*, go ahead and sit closer to me] as well as “Why does he want to look into my eyes? It’s depressing. Does not he believe me? I do not want this” [EN01 – Go ahead and face me, so I can see you]).

#### Group “Not Abused”

Following the same procedure as the analyses in the previous section, one-sample *t*-tests applying the Benjamini-Hochberg procedure were conducted for each item of the survey.

The one-sample *t*-tests on the scale measuring *well-being* revealed significant results for 55 of the 67 items representing the socio-emotional interview techniques. A further 12 of the items did not significantly differ from 0 and can therefore be assumed to have no effect on well-being. Of the 55 items, which did significantly differ from 0, 10 items differed in the negative direction (Cohen’s *d* ranging between −0.22 and −2.14), meaning that they significantly decreased participants’ well-being. The other 45 techniques significantly increased the participants’ well-being (Cohen’s *d* ranging between 0.27 and 1.83).

The same procedure was applied to the ratings on the scale *willingness to talk*. About 54 of the 67 items significantly differed from 0 in their ratings, while 13 items had no effect on participants’ willingness to talk. Of the 54 techniques that did differ significantly from 0, six did so in the negative direction of the scale (Cohen’s *d* ranging between −0.22 and −1.88), thus significantly decreasing the participants’ willingness to talk. On the other hand, the remaining 48 items significantly increased the participants’ willingness to talk (Cohen’s *d* ranging between 0.22 and 1.22).

Finally, the same procedure was applied to the ratings on the scale *perceived pressure*. Around 44 of the 67 items significantly differed from 0, while 27 items did not. Of the 41 items that did differ significantly from 0, 27 did so in the positive direction of the scale (Cohen’s *d* ranging between 0.28 and 1.53). In other words, 27 items significantly increased the perceived pressure to adhere to the interviewer’s expectations. The remaining 14 items significantly decreased the perceived pressure (Cohen’s *d* ranging between −0.24 and −0.86) thereby allowing participants to feel free to share their version of events even if this version contradicted the police’s suspicions (for the results per *t*-test refer to Appendix E).

**Figure 4:**
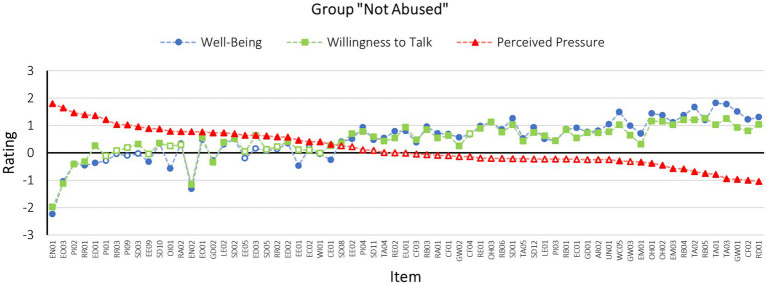
Scale means for each R-NICHD technique example as rated by the group “not abused” (*n* = 91). The items are sorted based on their rating on the scale perceived pressure. Filled dots refer to significant differences from 0 based on the results of one-sample *t*-tests corrected by means of the Benjamini-Hochberg procedure, while blank dots refer to non-significant differences from 0. For an explanation of the item codes, please refer to Appendix B.

Figure 4 depicts participants’ average ratings per item on each scale within the “not abused” control group. The items are again sorted based on their rating on the scale perceived pressure. It once more appears that the three scales are highly correlated with each other (see Table 1, below the diagonal). Thus, it also holds true for the “not abused” control group that items increasing the perceived pressure exerted by the interviewer were simultaneously rated as reducing both participants’ well-being and willingness to talk. On the other hand, items rated as increasing participants’ well-being and willingness to talk were again found to simultaneously reduce the perceived pressure by the interviewer.

Next, participants’ comments in the “not abused” control group were consulted. Items sorted on the right end of Figure 4, thus statements rated on average as significantly reducing the perceived pressure communicated by the interviewer, while increasing participants’ well-being and willingness to talk, were described as sensitive, thoughtful and appreciating (e.g., “Very sensitive” [CF02 – How are you feeling now that we are done?], “I think that’s thoughtful and nice” [RB04 – I can see what you are saying], “Appreciation of my information increases my well-being and reduces the pressure of expectations” [TA02 – I really appreciate that you have spoken to me]). Moreover, some participants indicated that they felt free to share their story, even if they could not confirm the police’s suspicion (e.g., “Even though I have reported the innocence of the teacher, my information is appreciated” [TA03 – Thanks for trying hard to remember and tell me what happened. Thank you for sharing with me]).

Items sorted at the left side of Figure 4 are again items that were observed to significantly increase the perceived pressure by the interviewer, while reducing participants’ well-being and in some cases their willingness to talk. It appears that these items were seen as pressuring, manipulative and sometimes even violating participants’ boundaries (e.g., “Here, too, I would feel under pressure again, as I could not confirm any accusations. The more often I would be asked such a question, the more this feeling would increase” [RR01 – You have told me a lot about yourself and I feel I know you better. Now that we know each other better you can share with me], “That feels pushy and assaulting!” [EN02 – *Name*, go ahead and sit closer to me], “Very manipulative and pressurizing. Would now think about ending the conversation” [EO03 – I am sure you could tell me]). Interestingly, one participant indicated that the implied assumption that something must have happened to him/her increased the willingness to talk, as s/he wanted to correct the interviewer’s wrong assumptions (“I get the feeling the cop thinks I do not have the guts to tell him I was abused. That’s why I’d like to set the record straight. That’s why I have an increased need to speak” [SD03 – *Name*, if something has happened to you and you want it to stop, you can tell me about it]). For item EN01 (Go ahead and face me so I can see you) participants, similarly as in the “abused” group, indicated that they felt confronted by the interviewer in a way that communicated disbelief in their denial of the suspicions (e.g., “Very uncomfortable, as if person would want to find out if I’m lying, assaulting”).

### Control Analyses: Distress, Sexual Victimization, Gender, and Occupation

The mean distress level as measured after completion of the survey was 21.41 on a scale from 1 to 101, with the most common answer being 1. Interestingly, participants in the “not abused” group (*M* = 25.11, *SD* = 26.74) experienced significantly more distress, *t*(165.91) = −2.09, *p* = 0.039, *d* = −0.31, than participants in the “abused” group (*M* = 17.90, *SD* = 19.88). This difference cannot be explained by differing numbers of people having actually experienced sexual violence across experimental groups (*n* = 17, i.e., 18% in the “abused” group vs. *n* = 18, i.e., 20% in the “not abused” group).

Even though no difference was expected, it was nonetheless controlled whether participants with own sexual violence experiences responded differently to the techniques in the survey as compared to participants without own sexual violence experiences. Participants who chose not to provide an answer concerning own sexual violence experiences were excluded from these specific analyses. In order to determine differences in well-being and willingness to talk before and after the survey, independent-samples Mann-Whitney *U*-tests were conducted as the ratings of initial well-being and willingness to talk were not normally distributed and the sample size was too small for one-way ANOVAs to be robust to this violation. The independent-samples Mann-Whitney *U*-tests examined the null-hypotheses that the distributions of well-being and willingness to talk, both before and after the survey, were the same across participants with and without own sexual violence experiences. None of the null hypotheses were rejected (*p* ranging from 0.067 to 0.628 for the different comparisons). In other words, no indications for participants with own sexual violence experiences responding differently to the survey as well as the rapport-building and supportive techniques in general emerged.

In order to determine whether sexual violence victims experienced more *distress* due to the survey than participants who had not experienced sexual violence themselves, independent-samples Mann-Whitney *U*-tests were conducted per experimental group, as the ratings of distress were not normally distributed. The null hypotheses, stating that there is no difference between participants with and without own sexual violence experiences in terms of the distress experienced, was rejected for the “abused” group (*U* = 289.0, *p* = 0.001, and *η*^2^ = 0.13) but not the “not abused” group (*U* = 465.5, *p* = 0.075, and *η*^2^ = 0.04). In other words, participants with own sexual violence experiences were significantly more distressed by the “abused”-vignette than other participants, while they did not respond differently to the “not abused”-vignette. Nonetheless, the average reported distress caused by the survey amongst sexual violence victims was still relatively low (*M* = 31.17 on a scale of 1 to 101).

Lastly, multiple regression analyses were performed for well-being as well as willingness to talk, both before and after the survey. Using the forced entry method, the vignette manipulation (hypothetically “abused” vs. “not abused”), gender, own sexual violence experiences, occupational field as well as occupational status were included as predictors. Participants who had chosen not to provide information on these variables were excluded from these analyses resulting in *n* = 176. All four regression analyses showed that the vignette manipulation was a significant predictor for well-being and willingness to talk, both before and after the survey (*t* ranging from 3.00 to 13.34, *p* ranging from <0.001 to 0.003 and *beta* ranging from 0.22 to 0.71). The multiple regression analyses additionally revealed a significant effect of gender on willingness to talk before (*t* = −3.06, *p* = 0.003, and *beta* = −0.17) and after the survey (*t* = −2.10, *p* = 0.037, and *beta* = −0.16), meaning that women in this sample reported significantly lower willingness to talk on both measurements, although the effect sizes are comparably small. As the distribution of gender is relatively equal across the “abused” and “not abused” control group (*n* = 76 vs. *n* = 77 for women and *n* = 18 vs. *n* = 14 for men, respectively), it is assumed that the effect of gender does not affect the results reported in previous sections. None of the other predictors were significant.

## Discussion

This study aimed to investigate the possible supportive and/or suggestive effects of each individual rapport-building and supportive technique formulated by the R-NICHD protocol, while for the first time including a control group having not experienced the abuse in question. Additionally, analyses were conducted in order to assess the overall effect of the survey, i.e., all socio-emotional interview techniques taken together, on participants’ well-being and willingness to talk.

The results suggested that the vignette-manipulation used in this study in order to create a hypothetical “abused” group and a hypothetical “not abused” control group was effective. Mixed factorial ANOVAs showed that the initial well-being and willingness to talk was lower in the “abused” compared to the “not abused” group, as would be expected for the comparison between real victims of CSA and interviewees who did not experience CSA.

Interestingly, however, while participants in the “abused” group reported a significantly higher well-being and willingness to talk after the survey, as was expected based on the previous positive results on the R-NICHD protocol, participants in the “not abused” group showed a smaller yet still significant increase in well-being but a significant decrease in willingness to talk at the end of the survey. There are several possible explanations for this result: The scale measuring the perceived pressure to adhere to the interviewer’s expectations could have had a different meaning for participants in the two groups. While participants in the “abused” group might have felt invited to share information they held but were not ready or willing to share yet, participants in the “not abused” group might have felt pressured to share information they simply did not hold and were unable to provide. Some comments provided by participants in the “not abused” group indicated that they felt that an implicit assumption that something must have happened to them was communicated in certain items (e.g., “Here, too, I would feel under pressure again, as I could not confirm any accusations. The more often I would be asked such a question, the more this feeling would increase,” comment regarding item RR01 – You have told me a lot about yourself and I feel I know you better. Now that we know each other better you can share with me). Thus, the perceived pressure in the “not abused” group might have caused them to feel that they were not listened to and their exonerating information was not taken seriously which could have decreased their willingness to talk. This could also explain the finding that participants in the “not abused” control group reported significantly higher distress as caused by the survey.

On the other hand, it is also possible that participants in the “not abused” group simply felt as if they had nothing left to share at the end of the survey, since nothing had happened to them. Either way, this result is not in line with our expectations and indicates that the earlier expressed concerns about the lack of a no-abuse control group in previous studies is valid. Research on the R-NICHD protocol as well as rapport-building and supportive techniques in general should not solely focus on corroborated cases of (sexual) abuse but preferably include both children who did experience an event and children who did not in an experimental study design. It is important that the R-NICHD protocol’s socio-emotional interview techniques are supportive in a non-suggestive way for children who did experience (sexual) abuse, but it should be considered equally important to test and ensure that these techniques are supportive and above all not suggestive for children who did not experience (sexual) abuse.

Upon comparing the results of both groups, it appears that participants in both groups showed a fair amount of agreement in terms of techniques they perceived as particularly good, thus supportive and non-suggestive, as well as particularly bad, thus non-supportive and suggestive (also refer to Appendix F). Techniques for which all examples provided by the R-NICHD protocol were universally perceived as supportive as well as non-suggestive include showing gestures of good will (e.g., “Are you cold? Would you like a short break?”), reinforcing behavior (e.g., “You corrected me and that is important”), expressing thanks and appreciation (e.g., “I really appreciate that you have spoken to me”), showing empathy (e.g., “I know it’s been a long interview”), checking on the interviewee’s feelings (e.g., “How are you doing so far?”), removing responsibility from the interviewee (e.g., “When things happen to children, it’s not their fault”) as well as offering help (e.g., “Begin talking and I’ll help with questions, I am here to help”). It appears that these positively rated items communicate thankfulness, appraisal, sympathy, stressing the interviewee’s self-efficacy, and control over the situation, while offering help. Additionally, these techniques include components of both rapport-building and support. All techniques focused on a friendly interaction as well as reinforcement and support of the interviewee. Thus, the evaluation of these techniques is in accordance with expectations derived from the literature regarding theoretical assumptions of rapport and support. This also corresponds with findings on the effects of rapport and support on interview outcomes (Saywitz et al., 2015, 2019; Blasbalg et al., 2018).

In contrast, all provided examples for the techniques involving expressions of personal interest (e.g., “I really want to get to know about things that happened to you. Today is the first time we have met and it is important for me to know you better”), reflecting on the relationship (e.g., “You have told me a lot about yourself and I feel I know you better. Now that we know each other better you can share with me”), expression of confidence and optimism (e.g., “I’m sure you could tell me”), encouraging non-verbal communication (“e.g., *Name*, go ahead and sit closer to me”) as well as encouraging disclosure (“It’s really important that you tell me if something is happening to you”) were perceived as pressuring and not-supportive across both groups. It appears that these techniques were perceived to violate physical and emotional boundaries and make use of a seemingly manipulative approach to extracting information along the lines of “Since you told me about A, now you can tell me about B as well.” Other negatively perceived techniques seem to overly focus on the wish of “really” wanting and/or needing to know. These techniques seemingly attempt to force rapport as well as overcome personal boundaries against the will of the interviewee, even though coercion and manipulation contradict the basic idea of rapport, which is characterized by mutual cooperation (Saywitz et al., 2015). These negative evaluations may be attributed to the fact that the techniques were designed for children with different developmental needs regarding cooperation and trust as compared to adults. Therefore, they might have been perceived as inappropriate by adults. On the other hand, it is also possible that the techniques have a similarly negative effect on children. Subsequently, further research on the techniques’ effects on other age groups is required.

For techniques ranked in the middle of the rating spectrum in Figures 3, 4, the two experimental groups showed less strong and consistent ratings in terms of the change in perceived pressure, well-being and willingness to talk and the results are therefore more difficult to interpret. It appears that these techniques have a positive effect on some participants, while at the same time having a negative effect on others.

Prior research did show positive results for the entire R-NICHD protocol (e.g., Hershkowitz et al., 2014, 2017; Blasbalg et al., 2018; Ahern et al., 2019) and this study confirmed that the protocol clearly contains socio-emotional interview techniques universally perceived as supportive and non-suggestive. Additionally, the rated techniques overall significantly increased participants’ well-being in both, the “abused” as well as the “not abused” group, while also increasing “abused” participants’ willingness to talk. Although the results must be interpreted with caution due to the study’s limitations, results also suggest that the protocol included several examples of techniques that were perceived as non-supportive, while being suggestive. Additionally, this study only measured perceived pressure exerted by the interviewer, which is a consciously perceivable aspect of suggestiveness. It can be argued that suggestiveness includes far more than consciously perceivable pressure and can also influence a person on a subconscious level, which could not be measured in this self-report based study. Therefore, the need for research on individual rapport-building and supportive techniques, preferably involving appropriate control groups, is stressed once more. This is of particular importance considering the added impetus of using the R-NICHD protocol to not only support disclosures but also moreover to create a willingness to disclose in reluctant children (Hershkowitz et al., 2014).

## Limitations and Suggestions for Future Research

This study contains several limitations. Firstly, while the R-NICHD protocol is intended for the use on children, adults were selected as participants in the current laboratory study. For ethical and practical reasons, it was decided not to use a sample consisting of child-participants in this first approach. However, the possibility that children would have responded differently to the techniques as compared to adults cannot be excluded. Furthermore, on 11 occasions (less than 1% of all comments) participants in this study actually noted that they did not appreciate certain items as they felt that they were being treated like a child. Nonetheless, it has to be noted that perceived pressure, even when only perceived by adults and going unnoticed by children, still counts as a form of suggestion which should be avoided. In fact, numerous studies show that children are more responsive to different kinds of suggestive influences (for an overview, see Volbert, 1995, 2015). Studies focusing on false memories in children regularly show higher rates of induced memory as compared to studies focusing on adults (Nichols and Loftus, 2019). The mere fact that children are used to adhere to adults’ expectations and most likely would not pick up on demanding questions in a negative way, does not warrant that they should be asked such questions as the result might still be a statement contaminated by suggestive influences. Additionally, it can be expected that techniques creating a sense of well-being and willingness to talk in adults would also have a positive effect on children. While it would be difficult to replicate this study with child-participants due to the required introspection skills and the severity of the addressed topic, future research focusing on the effects of the individual socio-emotional interview techniques postulated by the R-NICHD protocol and other interview protocols in children of different age groups is needed (Saywitz et al., 2015, 2019). This study cannot draw conclusions pertaining to developmental aspects of rapport-building and support. Additionally, future studies should focus on the effect of socio-emotional techniques on adolescents as field research has shown pronounced difficulties when it comes to building rapport with this specific age group (Collins et al., 2014). Moreover, the current study showed significant effects of gender on willingness to talk, meaning that women in this sample reported a significantly lower willingness to talk. However, these results are somewhat difficult to interpret due to the small number of men in our sample. Future research should take possible effects of gender into account when assessing rapport-building as well as support.

Secondly, the design of this study as well as certain aspects of the methodology do not necessarily translate into real-life situations and interviews without problems. The vignette-manipulation did not involve real experiences but an imagination task instead. Additionally, the items in the current study were presented in a randomized order to the participants. This was done to reduce order effects as well as the impact of fatigue on ratings. However, a randomized presentation of the items removes them from their context and might make some statements or questions appear somewhat artificial. Despite this, since the aim of the current study was to address the individual effects of the socio-emotional interview techniques, irrespective of their context, a randomized presentation was required and this limitation could not be avoided. Nonetheless, it cannot be excluded that some items might have been perceived differently when used in the appropriate moment during an actual interview. This should be noted especially for those items that are recommended for usage in specific situations only (e.g., when external evidence for CSA exists) or in response to specific statements by the interviewee. Furthermore, a real-life interview would most likely not involve all 67 examples or even all 26 rapport-building and supportive techniques but rather a sub-selection instead, depending on the interview-situation, developmental considerations or the level of reluctance displayed by the interviewee. In sum, the results have to be treated with caution, as it is possible that the evaluated techniques might have had different effects in a real-life interview as compared to this hypothetical study-scenario. Our study should not be taken as conclusive evidence that some techniques have the desired effects and others do not. Instead, this study aims to be the first to test whether each technique is perceived to be as supportive as intended. As perceived support has been found to be a stronger predictor for the interview outcome than the level of actual support provided, the interviewees’ perception of rapport and support may also provide valuable insights for studying the effects of socio-emotional interview techniques (Saywitz et al., 2015). It is suggested that future research should involve experimental laboratory studies, for example, by including some of the techniques and examples that were rated as having a particularly positive or negative effect on participants in this exploratory study, to validate these findings. Nevertheless, as usually only a limited number of techniques is used in an interview, the results suggest that the use of some rapport-building and supportive techniques may be preferred to others.

Thirdly, the study was conducted on a German sample. This is not *per se* a limitation, as the R-NICHD protocol has been translated into various languages worldwide and it is necessary to test its applicability in different cultures (La Rooy et al., 2015; Jud and Kindler, 2019). However, it should be noted that the R-NICHD protocol, as compared to the standard NICHD protocol, shifted in focus from mostly using cognitive techniques to additionally making extensive use of socio-emotional interview techniques. While techniques focusing on cognitive factors should have a fairly comparable effect across cultures, it is possible that specific socio-emotional interview techniques are perceived differently across cultural groups (Bowe et al., 2014). For example, it would have to be examined whether the negative ratings of specific techniques that express a particular personal interest, or are associated with great physical closeness, are due to cultural differences in the perception of personal space.

Lastly, recent research has shown the significance of including cultural and contextual aspects in the translation process when it comes to the translation of investigative interview protocols for children (IIPCs) as compared to a mere direct translation process (Navarro et al., 2019). Upon reading the official German translation of the R-NICHD protocol (Noeker and Franke, 2018), which was used in this study, some translations appeared to be either sub-optimal or too complicated due to the grammatical structure of some translated statements when compared to the English R-NICHD protocol. Therefore, future research should address whether the translation of the (German) R-NICHD protocol needs to be reassessed and possibly improved.

## Conclusion

The results of this laboratory study showed that the rapport-building and supportive techniques proposed by the R-NICHD protocol generally have a positive effect on interviewees. Many techniques had the intended supporting effect, increasing participants’ well-being and willingness to talk, while additionally encouraging them to share their version of events without pressure. Most importantly, it was shown that this positive effect extended across both, the “abused” as well as the “not abused” control group. However, the analyses also identified several techniques and statements that were perceived negatively by all participants. The conclusions need to be viewed in light of the study’s limitations, since they are drawn from evaluations by adults regarding hypothetical scenarios. Even if the results cannot be readily transferred to real-life interviews, they do stress the need to incorporate interviewees’ perceptions of interviewer rapport-building and supportive behavior in future research. Additionally, although no profound differences were found between the hypothetical “abused” and “non-abused” groups in the present study, the inclusion of appropriate control groups is deemed important whenever possible.

## Data Availability Statement

The raw data supporting the conclusions of this article will be made available by the authors, without undue reservation.

## Ethics Statement

The studies involving human participants were reviewed and approved by Ethics Committee of Psychologische Hochschule Berlin. The participants provided their written informed consent to participate in this study.

## Author Contributions

AT, JO, and RV contributed to the conception and design of the study, discussed the results, and commented on the manuscript. JO programmed the online survey, collected the data, performed the statistical analysis, and wrote the first draft of the manuscript. AT supervised all these steps and wrote sections of the manuscript. All authors contributed to the article and approved the submitted version.

### Conflict of Interest

The authors declare that the research was conducted in the absence of any commercial or financial relationships that could be construed as a potential conflict of interest.

## Footnotes

^1^http://nichdprotocol.com
