# Supplementary material for: Assessing the Individual Interviewer Rapport-Building and Supportive Techniques of the R-NICHD Protocol
Source: Front Psychol. 2021 Jul 21;12:659438. doi: 10.3389/fpsyg.2021.659438 (PMC8333708; doi:10.3389/fpsyg.2021.659438)
Supplement: Supplementary file 1 [file Data_Sheet_1.PDF]

Supplementary Material

This document contains the appendices to the paper "Assessing the Individual Interviewer Rapport-Building and Supportive Techniques of the R-NICHD Protocol".

1 Appendix A – The Vignettes as Presented to the Participants (Translation From German)

1.1 Group "Abused"

We kindly ask you to carefully read the following vignette and to picture yourself in the described situation. This is of utmost importance for the further proceedings in this survey.

Two weeks ago you were surprisingly contacted by the police. You were informed that your former primary school teacher is being suspected of having sexually abused several children. During the investigation it became clear that the teacher in question had been your class teacher in grade 3. For this reason the police invited you for an interview.

In grade 3 you were indeed sexually abused on several occasions by the accused primary school teacher. One time, for example, your teacher had asked you for a private conversation after class during one of the breaks. Once you were alone with him in the classroom, he had pulled down his pants and had forced you to look at and touch his penis. You never shared this incident or other sexually abusive experiences involving the accused teacher with anyone – neither your friends nor your family. You would prefer to simply forget about what has happened and you are not interested in suddenly opening up on this topic after so many years. Nonetheless, the interview with the police officer is going to take place today.

1.2 Group "Not Abused"

We kindly ask you to carefully read the following vignette and to picture yourself in the described situation. This is of utmost importance for the further proceedings in this survey.

Two weeks ago you were surprisingly contacted by the police. You were informed that your former primary school teacher is being suspected of having sexually abused several children. During the investigation it became clear that the teacher in question had been your class teacher in grade 3. For this reason the police invited you for an interview.

You are very surprised by the accusations against your primary school teacher. Even though you intensively thought about it, you simply cannot remember that you had ever experienced sexually abusive incidents in relation to the accused teacher. The only thing you can remember is that the teacher had always hugged you and other children, when you had fallen on the school's playground and hurt yourselves. Sometimes the teacher had also stroked your head to comfort you. Generally, you've always experienced the primary school teacher as a very caring person and you only have positive memories of him. Nonetheless, the interview with the police officer is going to take place today.

2 Appendix B – Included Examples for Each Technique per Category

Technique Category	Technique	Examples From the English R-NICHD Protocol	Included Examples From the German R-NICHD Protocol (Including Small Modifications for Use on Adults)	Item Code
Establishing Rapport	Welcoming the child	I am glad to [meet you today, to get to know you, to get to talk to you]. My name is _______.	Ich freue mich Sie heute zu sehen/Sie kennenzulernen/mit Ihnen sprechen zu können. Mein Name ist...	WC05
	Expression of personal interest in the child	I really want to get to know about you. Today is the first time we have met and it is important for me to know you better.	Ich möchte wirklich mehr über Sie erfahren. Heute ist zwar das erste Mal, dass wir uns sehen, aber es ist wichtig, dass ich mehr über Sie erfahre.	PI01
		[Name], [I am, people] are worried about you and I want to know if something may have happened to you.	[Ihr Name], [ich bin/Personen sind] Ihretwegen besorgt und ich möchte wissen, ob Ihnen vielleicht etwas zugestoßen ist.	PI09
		I really want to get to know about things that happened to you. Today is the first time we have met and it is important for me to know you better.	Ich möchte wirklich von den Sachen erfahren, die Ihnen widerfahren sind. Heute ist zwar das erste Mal, dass wir uns sehen, aber es ist wichtig, dass ich mehr über Sie erfahre.	PI02
		Now, [name], I want to get to know you better. Tell me about things you like to do.	Nun, liebe/r [Ihr Name], ich würde dich gerne besser kennenlernen. Erzählen Sie mir doch über Dinge, die Sie gerne machen.	PI03
		I am glad I can talk to you today [name].	Ich bin froh, dass ich heute mit Ihnen sprechen kann, [Ihr Name].	PI04
	Small gestures of good will	Are you cold? Would you like a short break?	Ist Ihnen kalt? Möchten Sie, dass wir eine kleine Pause machen?	GW01
		Are you comfortable?	Fühlen Sie sich wohl?	GW02
		Can I do anything to make you more comfortable?	Was kann ich machen, dass Sie sich wohler fühlen?	GW03
Reinforcement		You are describing it clearly.	Sie beschreiben es klar und deutlich.	RB01

	Reinforcing behavior	You are giving a lot of details and that's important.	Sie haben mir viele Details genannt, was sehr wichtig ist.	RB03
		You are really helping me understand, thank you.	Sie helfen mir wirklich sehr, Sie zu verstehen. Danke!	RB04
		You corrected me and that is important.	Sie haben mich korrigiert, was sehr wichtig ist.	RB05
		I can see what you're saying.	Ich verstehe, was Sie mir sagen möchten.	RB06
	Thanks and appreciation	I want to thank you for your help.	Ich möchte Ihnen für Ihre Hilfe danken.	TA01
		I really appreciate that you have spoken to me.	Ich weiß es wirklich zu schätzen, dass Sie mit mir darüber gesprochen haben.	TA02
		Thanks for trying hard to remember and tell me what happened. Thank you for sharing with me.	Danke, dass Sie sich bemüht haben, sich an alles zu erinnern und dass Sie mir erzählt haben, was passiert ist. Danke, dass Sie mir dies mitgeteilt haben.	TA03
		Thank you for sharing that with me, it helps me get to know you.	Danke, dass Sie mir dies mitgeteilt haben, dadurch lerne ich Sie besser kennen.	TA04
		I am glad I am starting to get to know more about you.	Es freut mich, dass ich mehr über Sie erfahren darf.	TA05
	Respect for the child's decisions	It's your choice whether to tell me or not, and it is my job to let you choose/and I will go with your choice.	Es ist ganz Ihre Entscheidung, ob Sie mir davon erzählen möchten oder nicht und ich werde Ihre Entscheidung akzeptieren/. Es ist nämlich mein Job Sie entscheiden zu lassen.	RD01
Using Rapport	Reflecting on the relationship	You have told me a lot about yourself and I feel I know you better. Now that we know each other better you can share with me.	Sie haben mir viel über sich erzählt und ich habe das Gefühl, Sie nun besser zu kennen. Nun, da wir uns besser kennen, können Sie mir von dem Ereignis erzählen.	RR01
		You told me a lot about yourself. I feel I know you better and you can tell me more [about things, about both good things and bad things] that have happened to you.	Sie haben mir viel über sich erzählt. Ich habe das Gefühl, ich kenne Sie nun besser und Sie können mir mehr [über	RR02

			Dinge, sei es gute oder schlechte Dinge] erzählen, die Ihnen widerfahren sind.	
		You told me a lot about yourself, thank you for letting me know. When you talk to me today please go on and tell me about other things that have happened to you.	Sie haben mir viel über sich erzählt, Danke, dass Sie mich diese Dinge haben wissen lassen. Wenn Sie jetzt mit mir sprechen, erzählen Sie mir über weitere Dinge, die Ihnen passiert sind.	RR03
	Expression of care	I'm here for you.	Ich bin für Sie da.	EC01
		I care about you.	Ich interessiere mich für Sie./Sie sind mir von Bedeutung.	EC02
	Emphasizing that you are someone to disclose to	If something happened I'm here to listen to you.	Wenn Ihnen etwas passiert ist, bin ich hier, um Ihnen zu zuhören.	SD01
		You can trust me and tell me if something happened.	Sie können mir vertrauen und mir ruhig sagen, falls etwas passiert sein sollte.	SD02
		[Name], if something has happened to you and you want it to stop, you can tell me about it.	[Ihr Name], wenn Ihnen etwas zugestoßen ist und Sie möchten, dass es aufhört, können Sie mir davon erzählen.	SD03
		I talk to many kids and they tell me about things that have happened to them.	Ich rede mit vielen Menschen und diese erzählen mir über Dinge, die ihnen widerfahren sind.	SD05
		[Name], my job is to listen to children about things that happened to them.	[Ihr Name], mein Job ist es Menschen zu zuhören, denen etwas widerfahren ist.	SD08
		[Name], I really want to know when something happens to children. That's what I am here for.	[Ihr Name], ich möchte es wirklich erfahren, wenn Menschen etwas zugestoßen ist. Dafür bin ich hier.	SD10
		[Name], here kids can talk about good things and bad things that have happened to them.	[Ihr Name], hier können Menschen über gute und schlechte Dinge sprechen, die sie erlebt haben.	SD11
		My job is to try to help kids.	Meine Arbeit ist es zu versuchen, Menschen zu helfen.	SD12
Emotional Support	Generalization of the child's difficulties	Many children find it difficult to tell.	Viele Menschen finden es schwer, darüber zu reden.	GD01
		Many children have secrets and I am here to listen.	Viele Menschen haben Geheimnisse und ich bin hier, um zu zuhören.	GD02

	Empathy	I know it's been a long interview.	Ich verstehe, dass das Interview ziemlich lange dauert.	EM01
		I understand that it is difficult for you to tell.	Ich verstehe, dass es Ihnen schwer fällt, darüber zu reden.	EM03
	Checking on the child's feelings	How are you doing so far?	Wie geht's Ihnen denn soweit?	CF01
		How are you feeling now that we are done?	Wir sind nun fertig mit dem Interview. Wie fühlen Sie sich nun?	CF02
		How did you feel before we talked?	Wie haben Sie sich gefühlt, bevor wir miteinander gesprochen haben?	CF03
		How did you feel when you were talking to me today?	Wie haben Sie sich gefühlt, als Sie heute mit mir gesprochen haben?	CF04
	Exploring Emotions	Tell me more about your [fear, anger].	Erzählen Sie mir mehr über [Ihre Ängste; Ihre Wut].	EE01
		I see you're crying. How come?	Ich sehe, Sie weinen gerade [oder andere emotionale Reaktion]. Was ist denn passiert?	EE05
		[Name] what would happen if you told me?	[Ihr Name], was würde (denn) passieren, wenn Sie mir davon erzählen würden?	EE09
		[Name] is there anything you are concerned about?	[Ihr Name], gibt es etwas, was Ihnen Sorgen bereitet?	EE02
	Echoing emotions	You said you were [sad, you cried, got angry].	Sie sagten, Sie waren traurig/wütend/dass Sie geweint haben.	CE01
	Open questions about feelings/thought s during the incident	You said you were [sad, grossed out, wanted to run away]. Tell me more about that.	Sie sagten Sie waren traurig/angeekelt/wollten weglaufen. Erzählen Sie mir mehr darüber.	OI01
	Accepting and recognizing emotions	I understand what you are saying.	Ich verstehe, was Sie mir versuchen zu sagen.	AR02
	Reassurance	Don't worry [Worry that the child mentions such as: I won't tell other children, I will make sure you won't be late to the bus, I will make sure nobody is going to arrest you, Sometimes it is possible to help families	Machen Sie sich keine Sorgen. [Ich werde anderen nichts erzählen/Sorge ansprechen die zuvor benannt wurde]./In manchen Fällen, ist es möglich [Familien	RA01

		with problems/the people who have hurt others].	mit Problemen/Leuten, die anderen wehgetan haben zu helfen.].	
		Sometimes it helps children when they can talk and do not have to keep a secret.	Manchmal hilft es Menschen, wenn sie über etwas sprechen können und kein Geheimnis für sich behalten müssen.	RA02
	Removing responsibility from the child	If [something happened, someone hurt you], [it is not your fault, you are not responsible for that].	Falls [etwas passiert ist; jemand Ihnen wehgetan hat], [ist es nicht Ihre Schuld, denn Sie sind nicht dafür verantwortlich.]	RE01
		When things happen to children, it's not their fault.	Generalisierung: Wenn Kindern etwas zustößt, ist es nicht deren Fehler.	RE02
	Exploring unexpressed emotions	If it is hard for you to talk about it, what you are concerned about?	Falls es Ihnen schwer fallen sollte, darüber zu sprechen, erzählen Sie mir was Ihnen Sorgen bereitet.	EU01
Kind encouragement	Warmly emphasizing that the child is the key source of information	I am asking you these questions because I was not there.	Ich stelle Ihnen diese Fragen, da ich nicht dabei war.	WI01
	Legitimating expressions	You can talk about bad things and good things.	Sie können mit mir über gute und schlechte Dinge sprechen.	LE01
		In this office you can say anything.	In diesem Büro können Sie wirklich alles sagen.	LE02
	Expression of confidence/optimism	I think you can describe it well.	Ich bin mir sicher, dass Sie es gut beschreiben können.	EO01
		I'm sure you could tell me.	Ich bin mir sicher, dass Sie es mir sagen können.	EO03
	Offering help	I want to make it easier for you, how can I help you tell me?	Ich möchte es für Sie leichter gestalten, wie kann ich Ihnen helfen?	OH01
		Would it be easier if you wrote it?	Wäre es für Sie einfacher, wenn Sie es selbst aufschreiben?	OH02
		Begin talking and I'll help with questions, I am here to help.	Fangen Sie ruhig an zu sprechen und ich werde Ihnen helfen, indem ich Ihnen Fragen stelle. Ich bin hier, um zu helfen.	OH03

	Encouraging non-verbal communication	Go ahead and face me, so I can see you.	Erzählen Sie ruhig weiter und schauen Sie mich dabei an, sodass ich Ihnen in die Augen schauen kann.	EN01
		[Name], go ahead and sit closer to me.	[Ihr Name], erzählen Sie weiter und setzen Sie sich ruhig neben mich.	EN02
	Encouraging disclosure	It's really important that you tell me if something is happening to you.	Es ist von großer Bedeutung, dass Sie mir sagen, falls Ihnen etwas passiert ist.	ED01
		[Name], if there is anything you want to tell me, [I want to know/listen, It's important for me to know/listen]	[Ihr Name], falls es irgendetwas geben sollte, was Sie mir sagen möchten, möchte ich dies wissen/Ihnen zuhören. Es ist wichtig, dass ich dies weiß/dass ich Ihnen zuhöre.	ED02
		Please go ahead and tell me.	Bitte fahren Sie fort und erzählen Sie mir mehr.	ED03
Addressing the Child by Name	Using the interviewee's name	Using the interviewee’s name provided along with German examples to illustrate.	Beim Namen angesprochen werden: Vielen Dank, dass Sie gekommen sind, [Ihr Name]. oder Wenn ich Sachen falsch sage, sagen Sie mir bitte Bescheid, ja, [Ihr Name]?	UN01

3 Appendix C – Excluded Examples for Each Technique per Category

Technique Category	Technique	Excluded Examples
Establishing Rapport	Small gestures of goodwill	Let me show you where the bathroom is.
		You can look at the video camera if you want.
Reinforcement	Respect for the child's decision	It's your choice whether to tell and I will go with your choice.
Using Rapport	Expression of care	You are important to me.
	Emphasizing that you're someone to disclose to	It’s my job to listen to children if they have a problem.
Emotional Support<br>´	Generalization of the child's expressed difficulties	Many children feel [ashamed, shy, a bit quiet] at the beginning but then it’s fine.
		Many children are [difficulty the child mentioned] and I try to help them.
	Accepting and recognizing emotions	I can see what you are saying.
		You said that it was very painful.
		[Child’s name], if it is hard for you to tell, what makes it so hard?
	Removing responsibility from the child	Sometimes children think that if something happened to them, it's their fault, but children are not responsible if things happen to them.
	Exploring Emotions	You said you cannot tell me. Tell me more about that.
Kind encouragement	Legitimating expressions	It is ok to talk [about this kind of thing, to say those/bad words].
	Encouraging non-verbal communication	Let me see your eyes.

4 Appendix D – Results One-Sample t-Tests per Item Group “Abused” Sorted Based on Perceived Pressure in Ascending Order

	Well-being					Willingness to talk					Perceived pressure				
**Technique**	*M*	*SD*	*t*	*p (sig.)*	*d*	*M*	*SD*	*t*	*p (sig.)*	*d*	*M*	*SD*	*t*	*p (sig.)*	*d*
**CF02**	1.27	1.33	9.39	0.000	0.96	0.75	1.20	6.14	0.000	0.63	-1.29	1.50	-8.44	0.000	-0.86
**GW01**	1.61	1.01	15.68	0.000	1.60	0.95	0.97	9.61	0.000	0.98	-1.24	1.00	-12.11	0.000	-1.24
**RD01**	1.38	1.31	10.30	0.000	1.05	1.11	1.38	7.94	0.000	0.81	-1.08	1.67	-6.35	0.000	-0.65
**TA03**	1.47	0.95	15.13	0.000	1.54	1.24	0.98	12.38	0.000	1.26	-1.04	1.22	-8.35	0.000	-0.85
**TA01**	1.74	1.04	16.41	0.000	1.67	1.16	1.05	10.79	0.000	1.10	-0.92	1.25	-7.21	0.000	-0.74
**TA02**	1.68	0.86	19.01	0.000	1.94	1.14	0.88	12.67	0.000	1.29	-0.89	1.20	-7.21	0.000	-0.74
**RB04**	1.21	1.09	10.82	0.000	1.10	1.04	1.00	10.16	0.000	1.04	-0.84	0.99	-8.37	0.000	-0.85
**OH01**	1.61	0.94	16.75	0.000	1.71	1.30	0.91	14.06	0.000	1.43	-0.82	1.21	-6.69	0.000	-0.68
**RB05**	1.04	0.92	11.13	0.000	1.14	1.05	0.92	11.18	0.000	1.14	-0.65	1.16	-5.45	0.000	-0.56
**OH02**	1.31	1.19	10.65	0.000	1.09	1.13	1.14	9.62	0.000	0.99	-0.61	1.30	-4.58	0.000	-0.47
**EM03**	0.97	1.22	7.79	0.000	0.80	0.70	1.04	6.59	0.000	0.67	-0.59	1.17	-4.95	0.000	-0.51
**GW03**	1.35	1.10	12.01	0.000	1.23	0.99	1.10	8.81	0.000	0.90	-0.58	1.33	-4.31	0.000	-0.44
**AR02**	1.00	1.23	7.96	0.000	0.81	0.86	1.22	6.95	0.000	0.71	-0.58	1.16	-4.94	0.000	-0.50
**RE01**	1.22	1.30	9.19	0.000	0.94	1.09	1.15	9.30	0.000	0.95	-0.48	1.28	-3.66	0.000	-0.37
**EU01**	1.06	1.16	8.98	0.000	0.92	1.09	1.12	9.53	0.000	0.97	-0.46	1.34	-3.36	0.001	-0.34
**CF04**	0.75	1.28	5.74	0.000	0.59	0.77	1.03	7.33	0.000	0.75	-0.45	1.30	-3.36	0.001	-0.34
**RB06**	1.10	1.05	10.29	0.000	1.05	0.91	1.05	8.48	0.000	0.87	-0.44	1.04	-4.10	0.000	-0.42
**RE02**	0.78	1.39	5.48	0.000	0.56	0.57	1.23	4.49	0.000	0.46	-0.42	1.10	-3.74	0.000	-0.38
**GD01**	0.79	1.26	6.18	0.000	0.63	0.73	1.05	6.80	0.000	0.69	-0.41	1.34	-2.97	0.004	-0.30
**PI03**	0.35	1.59	2.18	0.031	0.22	0.33	1.66	1.97	0.052	0.20	-0.39	1.49	-2.54	0.013	-0.26
**EC01**	0.71	1.39	4.99	0.000	0.51	0.42	1.32	3.10	0.003	0.32	-0.38	1.23	-2.98	0.004	-0.30
**RA01**	0.66	1.65	3.90	0.000	0.40	0.67	1.53	4.28	0.000	0.44	-0.36	1.10	-3.26	0.002	-0.33
**UN01**	0.96	1.21	7.74	0.000	0.79	0.85	1.14	7.33	0.000	0.75	-0.31	1.15	-2.65	0.009	-0.27

**CF03**	0.51	1.25	4.01	0.000	0.41	0.63	1.09	5.63	0.000	0.57	-0.29	1.16	-2.46	0.016	-0.25
**TA05**	0.45	1.34	3.29	0.001	0.34	0.38	1.29	2.86	0.005	0.29	-0.26	1.12	-2.29	0.025	-0.23
**WC05**	1.22	0.98	12.24	0.000	1.25	0.80	0.96	8.20	0.000	0.84	-0.25	0.99	-2.46	0.016	-0.25
**RB01**	0.49	1.21	3.98	0.000	0.41	0.58	1.16	4.94	0.000	0.50	-0.23	1.13	-1.99	0.049*	-0.20
**CF01**	0.40	1.22	3.18	0.002	0.32	0.39	1.06	3.56	0.001	0.36	-0.20	0.99	-1.96	0.053	-0.20
**LE01**	0.63	1.10	5.58	0.000	0.57	0.61	1.09	5.53	0.000	0.56	-0.19	1.19	-1.54	0.126	-0.16
**SD12**	0.66	1.11	5.78	0.000	0.59	0.54	1.12	4.73	0.000	0.48	-0.18	1.07	-1.63	0.107	-0.17
**RB03**	0.95	0.99	9.40	0.000	0.96	0.99	0.92	10.50	0.000	1.07	-0.16	1.08	-1.42	0.159	-0.14
**TA04**	0.48	1.10	4.25	0.000	0.43	0.38	1.10	3.35	0.001	0.34	-0.16	0.94	-1.62	0.108	-0.17
**OH03**	1.03	1.11	9.11	0.000	0.93	1.19	1.02	11.42	0.000	1.17	-0.15	1.29	-1.11	0.271	-0.11
**CE01**	-0.13	1.20	-1.02	0.309	-0.10	0.43	1.19	3.53	0.001	0.36	-0.13	1.04	-1.18	0.241	-0.12
**EM01**	0.45	1.19	3.70	0.000	0.38	0.19	0.97	1.90	0.060	0.19	-0.11	1.07	-1.05	0.299	-0.11
**SD01**	1.07	1.03	10.22	0.000	1.04	1.01	1.03	9.60	0.000	0.98	-0.09	1.27	-0.73	0.470	-0.07
**SD11**	0.38	1.10	3.35	0.001	0.34	0.32	1.24	2.56	0.012	0.26	-0.06	1.17	-0.52	0.601	-0.05
**EE02**	0.35	1.25	2.78	0.007	0.28	0.71	1.12	6.18	0.000	0.63	-0.04	1.24	-0.33	0.743	-0.03
**EC02**	0.34	1.55	2.18	0.032	0.22	0.23	1.44	1.56	0.122	0.16	-0.02	1.28	-0.16	0.874	-0.02
**GW02**	0.31	1.31	2.34	0.021	0.24	0.20	1.16	1.68	0.097	0.17	0.07	1.20	0.60	0.553	0.06
**SD08**	0.21	1.20	1.71	0.091	0.17	0.20	1.24	1.56	0.123	0.16	0.25	1.26	1.94	0.056	0.20
**PI04**	0.77	1.03	7.33	0.000	0.75	0.60	0.98	6.05	0.000	0.62	0.26	1.04	2.46	0.016	0.25
**SD02**	0.36	1.31	2.73	0.007	0.28	0.33	1.21	2.70	0.008	0.28	0.40	1.34	2.89	0.005	0.30
**EE01**	-0.08	1.25	-0.65	0.516	-0.07	0.51	1.28	3.90	0.000	0.40	0.42	1.18	3.45	0.001	0.35
**RR02**	0.25	1.26	1.95	0.054	0.20	0.27	1.26	2.11	0.038	0.21	0.44	1.30	3.29	0.001	0.34
**SD05**	0.09	1.38	0.66	0.509	0.07	0.25	1.31	1.88	0.064	0.19	0.45	1.24	3.54	0.001	0.36
**WI01**	-0.34	0.96	-3.51	0.001	-0.36	0.05	1.10	0.46	0.643	0.05	0.45	1.04	4.20	0.000	0.43
**LE02**	0.31	1.32	2.33	0.022	0.24	0.48	1.35	3.49	0.001	0.36	0.45	1.45	3.03	0.003	0.31
**EE05**	-0.29	1.46	-1.96	0.053	-0.20	0.04	1.34	0.31	0.761	0.03	0.46	1.27	3.53	0.001	0.36

**EE09**	-0.28	1.26	-2.18	0.032	-0.22	0.47	1.40	3.28	0.001	0.34	0.47	1.35	3.40	0.001	0.35
**RA02**	0.59	1.24	4.71	0.000	0.48	0.64	1.20	5.20	0.000	0.53	0.55	1.18	4.59	0.000	0.47
**ED02**	0.28	1.39	1.98	0.050*	0.20	0.49	1.47	3.26	0.002	0.33	0.59	1.30	4.47	0.000	0.46
**OI01**	-0.42	1.39	-2.94	0.004	-0.30	0.41	1.36	2.93	0.004	0.30	0.60	1.20	4.93	0.000	0.50
**SD03**	0.10	1.43	0.72	0.476	0.07	0.25	1.33	1.84	0.069	0.19	0.61	1.29	4.66	0.000	0.48
**GD02**	-0.01	1.42	-0.07	0.943	-0.01	-0.08	1.32	-0.62	0.537	-0.06	0.67	1.15	5.69	0.000	0.58
**SD10**	0.30	1.13	2.63	0.010	0.27	0.29	1.25	2.29	0.024	0.23	0.68	1.24	5.33	0.000	0.54
**ED03**	0.07	0.99	0.72	0.471	0.07	0.51	1.20	4.18	0.000	0.43	0.72	1.01	6.96	0.000	0.71
**EO01**	0.26	1.28	1.99	0.05*	0.20	0.27	1.28	2.08	0.04*	0.21	0.83	1.32	6.19	0.000	0.63
**PI09**	-0.04	1.44	-0.28	0.778	-0.03	0.22	1.22	1.76	0.081	0.18	0.84	1.09	7.59	0.000	0.77
**EN02**	-1.33	1.51	-8.68	0.000	-0.89	-1.05	1.45	-7.09	0.000	-0.72	0.89	1.43	6.08	0.000	0.62
**RR03**	-0.06	1.24	-0.50	0.622	-0.05	-0.03	1.21	-0.25	0.801	-0.03	0.91	1.20	7.42	0.000	0.76
**RR01**	-0.74	1.40	-5.17	0.000	-0.53	-0.54	1.36	-3.90	0.000	-0.40	1.22	1.21	9.89	0.000	1.01
**ED01**	-0.46	1.12	-4.00	0.000	-0.41	0.21	1.38	1.48	0.141	0.15	1.41	1.24	11.15	0.000	1.14
**PI01**	-0.41	1.23	-3.24	0.002	-0.33	-0.09	1.24	-0.74	0.461	-0.08	1.44	1.05	13.36	0.000	1.36
**PI02**	-0.51	1.35	-3.70	0.000	-0.38	-0.20	1.42	-1.37	0.175	-0.14	1.45	1.07	13.20	0.000	1.35
**EO03**	-0.93	1.24	-7.32	0.000	-0.75	-0.74	1.28	-5.65	0.000	-0.58	1.48	1.18	12.30	0.000	1.26
**EN01**	-2.25	0.93	-23.73	0.000	-2.42	-1.97	1.16	-16.56	0.000	-1.69	1.81	1.32	13.41	0.000	1.37

Note. *These items are no longer considered significant when applying the Benjamini-Hochberg procedure.

5 Appendix E – Results One-Sample t-Tests per Item Group “Not Abused” Sorted Based on Perceived Pressure in Ascending Order

	Well-being					Willingness to talk					Perceived pressure				
**Technique**	*M*	*SD*	*t*	*p (sig)*	*d*	*M*	*SD*	*t*	*p (sig.)*	*d*	*M*	*SD*	*t*	*p (sig.)*	*d*
**RD01**	1.30	1.47	8.40	0.000	0.88	1.03	1.30	7.56	0.000	0.79	-1.05	1.65	-6.10	0.000	-0.64
**CF02**	1.21	1.22	9.48	0.000	0.99	0.80	1.16	6.62	0.000	0.69	-1.00	1.37	-6.94	0.000	-0.73
**GW01**	1.51	1.12	12.83	0.000	1.35	0.92	1.07	8.25	0.000	0.87	-0.97	1.14	-8.09	0.000	-0.85
**TA03**	1.78	0.98	17.41	0.000	1.83	1.25	1.03	11.62	0.000	1.22	-0.95	1.10	-8.20	0.000	-0.86
**TA01**	1.82	1.01	17.29	0.000	1.81	1.02	1.21	8.05	0.000	0.84	-0.79	1.22	-6.21	0.000	-0.65
**RB05**	1.19	1.19	9.50	0.000	1.00	1.26	1.15	10.46	0.000	1.10	-0.75	1.30	-5.46	0.000	-0.57
**TA02**	1.67	1.00	15.92	0.000	1.67	1.20	1.09	10.51	0.000	1.10	-0.69	1.06	-6.22	0.000	-0.65
**RB04**	1.37	1.19	11.02	0.000	1.15	1.20	1.11	10.32	0.000	1.08	-0.59	1.27	-4.45	0.000	-0.47
**EM03**	1.11	1.17	9.06	0.000	0.95	1.02	1.02	9.54	0.000	1.00	-0.57	1.21	-4.50	0.000	-0.47
**OH02**	1.37	1.20	10.93	0.000	1.15	1.15	1.20	9.16	0.000	0.96	-0.45	1.32	-3.26	0.002	-0.34
**OH01**	1.44	1.15	11.97	0.000	1.26	1.16	1.08	10.31	0.000	1.08	-0.38	1.36	-2.69	0.009	-0.28
**EM01**	0.70	1.22	5.52	0.000	0.58	0.31	1.14	2.57	0.012	0.27	-0.34	1.14	-2.86	0.005	-0.30
**GW03**	0.99	1.30	7.28	0.000	0.76	0.64	1.25	4.86	0.000	0.51	-0.32	1.45	-2.09	0.039*	-0.22
**WC05**	1.49	1.07	13.34	0.000	1.40	1.02	1.07	9.07	0.000	0.95	-0.29	1.08	-2.53	0.013	-0.27
**GD01**	0.77	1.15	6.40	0.000	0.67	0.73	1.11	6.25	0.000	0.66	-0.25	1.34	-1.80	0.075	-0.19
**AR02**	0.80	1.42	5.38	0.000	0.56	0.73	1.39	4.97	0.000	0.52	-0.25	1.22	-1.98	0.051	-0.21
**UN01**	1.05	1.34	7.48	0.000	0.78	0.77	1.11	6.63	0.000	0.70	-0.25	1.05	-2.30	0.024	-0.24
**EC01**	0.91	1.32	6.58	0.000	0.69	0.54	1.21	4.23	0.000	0.44	-0.24	1.06	-2.18	0.032*	-0.23
**PI03**	0.44	1.58	2.66	0.009	0.28	0.44	1.74	2.41	0.018	0.25	-0.23	1.44	-1.53	0.129	-0.16
**RB01**	0.85	1.23	6.57	0.000	0.69	0.84	1.27	6.29	0.000	0.66	-0.23	1.29	-1.70	0.092	-0.18
**SD12**	0.92	1.19	7.43	0.000	0.78	0.74	1.16	6.04	0.000	0.63	-0.22	1.09	-1.92	0.058	-0.20
**SD01**	1.26	1.13	10.63	0.000	1.11	1.02	1.11	8.82	0.000	0.92	-0.21	1.30	-1.54	0.128	-0.16

**TA05**	0.53	1.44	3.49	0.001	0.37	0.43	1.38	2.96	0.004	0.31	-0.21	1.01	-1.98	0.051	-0.21
**OH03**	1.11	1.26	8.40	0.000	0.88	1.13	1.19	9.11	0.000	0.96	-0.20	1.36	-1.39	0.169	-0.15
**RB06**	0.86	1.23	6.62	0.000	0.69	0.75	1.24	5.73	0.000	0.60	-0.20	1.10	-1.72	0.089	-0.18
**GW02**	0.56	1.39	3.84	0.000	0.40	0.25	1.32	1.82	0.071	0.19	-0.13	1.40	-0.90	0.371	-0.09
**CF04**	0.67	1.35	4.74	0.000	0.50	0.70	1.23	5.44	0.000	0.57	-0.13	1.30	-0.97	0.336	-0.10
**LE01**	0.61	1.21	4.81	0.000	0.51	0.72	1.20	5.71	0.000	0.60	-0.12	1.38	-0.84	0.403	-0.09
**CF01**	0.69	1.09	6.05	0.000	0.63	0.63	1.05	5.69	0.000	0.60	-0.10	1.04	-0.90	0.369	-0.09
**RA01**	0.71	1.60	4.26	0.000	0.45	0.54	1.58	3.25	0.002	0.34	-0.09	1.31	-0.64	0.522	-0.07
**RE01**	1.09	1.29	8.03	0.000	0.85	1.00	1.15	8.24	0.000	0.87	-0.09	1.22	-0.69	0.493	-0.07
**RB03**	0.96	1.16	7.84	0.000	0.82	0.84	1.10	7.26	0.000	0.76	-0.07	1.13	-0.56	0.580	-0.06
**CF03**	0.38	1.34	2.74	0.007	0.29	0.47	1.16	3.89	0.000	0.41	-0.05	1.15	-0.46	0.649	-0.05
**EU01**	0.80	1.19	6.46	0.000	0.68	0.93	1.29	6.91	0.000	0.72	-0.01	1.26	-0.08	0.934	-0.01
**RE02**	0.79	1.35	5.57	0.000	0.58	0.54	1.31	3.92	0.000	0.41	0.00	1.34	0.00	1.000	0.00
**TA04**	0.54	1.34	3.82	0.000	0.40	0.43	1.17	3.51	0.001	0.37	0.01	1.04	0.10	0.920	0.01
**SD11**	0.47	1.27	3.56	0.001	0.37	0.58	1.27	4.36	0.000	0.46	0.09	1.32	0.63	0.527	0.07
**PI04**	0.93	1.20	7.43	0.000	0.78	0.78	1.00	7.46	0.000	0.78	0.11	1.03	1.02	0.310	0.11
**EE02**	0.51	1.14	4.23	0.000	0.44	0.70	1.09	6.15	0.000	0.65	0.23	1.11	1.99	0.050*	0.21
**SD08**	0.41	1.28	3.03	0.003	0.32	0.36	1.28	2.71	0.008	0.28	0.26	1.33	1.89	0.062	0.20
**CE01**	-0.26	1.08	-2.32	0.022	-0.24	0.26	1.06	2.37	0.020	0.25	0.31	1.02	2.88	0.005	0.30
**EC02**	0.19	1.66	1.07	0.286	0.11	0.10	1.61	0.59	0.558	0.06	0.41	1.44	2.70	0.008	0.28
**WI01**	-0.05	1.29	-0.41	0.686	-0.04	-0.01	1.43	-0.07	0.942	-0.01	0.41	1.30	2.99	0.004	0.31
**EE01**	-0.38	1.20	-3.00	0.004	-0.32	0.21	1.41	1.42	0.159	0.15	0.57	1.23	4.38	0.000	0.46
**RR02**	0.13	1.19	1.05	0.295	0.11	0.23	1.26	1.75	0.083	0.18	0.59	1.26	4.48	0.000	0.47
**SD05**	0.08	1.18	0.62	0.534	0.07	0.12	1.33	0.87	0.389	0.09	0.62	1.23	4.78	0.000	0.50
**EE05**	-0.20	1.48	-1.28	0.205	-0.13	0.05	1.36	0.39	0.701	0.04	0.64	1.20	5.08	0.000	0.53
**ED03**	0.16	1.05	1.50	0.136	0.16	0.63	1.23	4.87	0.000	0.51	0.64	1.20	5.08	0.000	0.53

**ED02**	0.46	1.16	3.72	0.000	0.39	0.51	1.19	4.07	0.000	0.43	0.68	1.28	5.03	0.000	0.53
**SD02**	0.51	1.39	3.46	0.001	0.36	0.52	1.35	3.64	0.000	0.38	0.70	1.08	6.21	0.000	0.65
**GD02**	-0.29	1.34	-2.03	0.046*	-0.21	-0.35	1.41	-2.38	0.019	-0.25	0.73	1.38	5.00	0.000	0.52
**LE02**	0.30	1.41	2.01	0.048*	0.21	0.38	1.38	2.66	0.009	0.28	0.73	1.31	5.29	0.000	0.55
**EO01**	0.48	1.24	3.72	0.000	0.39	0.57	1.30	4.19	0.000	0.44	0.77	1.37	5.37	0.000	0.56
**RA02**	0.34	1.40	2.32	0.023	0.24	0.29	1.45	1.88	0.063	0.20	0.78	1.14	6.51	0.000	0.68
**OI01**	-0.57	1.32	-4.14	0.000	-0.43	0.25	1.43	1.68	0.096	0.18	0.79	1.24	6.07	0.000	0.64
**SD10**	0.34	1.27	2.57	0.012	0.27	0.35	1.29	2.61	0.011	0.27	0.88	1.15	7.27	0.000	0.76
**EE09**	-0.33	1.29	-2.44	0.017	-0.26	-0.05	1.39	-0.38	0.708	-0.04	0.89	1.29	6.56	0.000	0.69
**EN02**	-1.23	1.49	-7.84	0.000	-0.83	-1.08	1.33	-7.71	0.000	-0.81	0.89	1.18	7.12	0.000	0.75
**SD03**	-0.03	1.42	-0.22	0.825	-0.02	0.31	1.40	2.10	0.038	0.22	0.96	1.21	7.54	0.000	0.79
**PI09**	-0.10	1.48	-0.64	0.524	-0.07	0.19	1.49	1.20	0.235	0.13	1.02	1.22	7.99	0.000	0.84
**RR03**	-0.04	1.21	-0.35	0.730	-0.04	0.08	1.28	0.57	0.569	0.06	1.04	1.23	8.11	0.000	0.85
**PI01**	-0.29	1.35	-2.02	0.047*	-0.21	-0.11	1.31	-0.80	0.426	-0.08	1.21	1.12	10.29	0.000	1.08
**RR01**	-0.46	1.29	-3.43	0.001	-0.36	-0.33	1.51	-2.09	0.040	-0.22	1.40	1.26	10.61	0.000	1.11
**PI02**	-0.40	1.32	-2.85	0.005	-0.30	-0.42	1.42	-2.80	0.006	-0.29	1.46	1.09	12.81	0.000	1.34
**ED01**	-0.28	1.25	-2.10	0.038	-0.22	0.37	1.38	2.53	0.013	0.27	1.48	1.18	11.86	0.000	1.25
**EO03**	-1.05	1.33	-7.58	0.000	-0.79	-1.12	1.41	-7.57	0.000	-0.79	1.64	1.34	11.68	0.000	1.22
**EN01**	-2.24	1.05	-20.42	0.000	-2.14	-1.98	1.05	-17.91	0.000	-1.88	1.80	1.18	14.62	0.000	1.53

Note. *These items are no longer considered significant when applying the Benjamini-Hochberg procedure.

6 Appendix F – Results for Each Scale per Technique and Group Sorted Based on Perceived Pressure in Group "Abused" in Ascending Order

				Perceived Pressure		Well-Being		Willingness to talk	
Category	Technique	Example	Item Code	A	NA	A	NA	A	NA
Emotional Support	Checking on the child's feelings	How are you feeling now that we are done?	CF02	—	—	+	+	+	+
Establishing Rapport	Small gestures of good will	Are you cold? Would you like a short break?	GW01	—	—	+	+	+	+
Reinforcement	Respect for the child's decisions	It's your choice whether to tell me or not, and it is my job to let you choose/and I will go with your choice.	RD01	—	—	+	+	+	+
Reinforcement	Thanks and appreciation	Thanks for trying hard to remember and tell me what happened. Thank you for sharing with me.	TA03	—	—	+	+	+	+
Reinforcement	Thanks and appreciation	I want to thank you for your help.	TA01	—	—	+	+	+	+
Reinforcement	Thanks and appreciation	I really appreciate that you have spoken to me.	TA02	—	—	+	+	+	+
Reinforcement	Reinforcing behavior	You are really helping me understand, thank you.	RB04	—	—	+	+	+	+
Kind encouragement	Offering help	I want to make it easier for you, how can I help you tell me?	OH01	—	—	+	+	+	+
Reinforcement	Reinforcing behavior	You corrected me and that is important.	RB05	—	—	+	+	+	+
Kind encouragement	Offering help	Would it be easier if you wrote it?	OH02	—	—	+	+	+	+
Emotional Support	Empathy	I understand that it is difficult for you to tell.	EM03	—	—	+	+	+	+
Emotional Support	Accepting and recognizing emotions	I understand what you are saying.	AR02	—	/	+	+	+	+
Establishing Rapport	Small gestures of good will	Can I do anything to make you more comfortable?	GW03	—	/	+	+	+	+
Emotional Support	Removing responsibility from the child	If [something happened, someone hurt you], [it is not your fault, you are not responsible for that].	RE01	—	/	+	+	+	+
Emotional Support	Exploring unexpressed emotions	If it is hard for you to talk about it, what you are concerned about?	EU01	—	/	+	+	+	+

Emotional Support	Checking on the child's feelings	How did you feel when you were talking to me today?	CF04	—	/	+	+	+	+
Reinforcement	Reinforcing behavior	I can see what you're saying.	RB06	—	/	+	+	+	+
Emotional Support	Removing responsibility from the child	When things happen to children, it's not their fault.	RE02	—	/	+	+	+	+
Emotional Support	Generalization of the child's difficulties	Many children find it difficult to tell.	GD01	—	/	+	+	+	+
Establishing Rapport	Expression of personal interest in the child	Now, [name], I want to get to know you better. Tell me about things you like to do.	PI03	—	/	+	+	+	+
Using Rapport	Expression of care	I'm here for you.	EC01	—	/	+	+	+	+
Emotional Support	Reassurance	Don’t worry [Worry that the child mentions such as: I won’t tell other children, I will make sure you won’t be late to the bus, I will make sure nobody is going to arrest you, Sometimes it is possible to help families with problems/the people who have hurt others].	RA01	—	/	+	+	+	+
Addressing the Child by Name	Using the interviewee's name	Using the interviewee’s name provided along with German examples to illustrate.	UN01	—	—	+	+	+	+
Emotional Support	Checking on the child's feelings	How did you feel before we talked?	CF03	—	/	+	+	+	+
Reinforcement	Thanks and appreciation	I am glad I am starting to get to know more about you.	TA05	—	/	+	+	+	+
Establishing Rapport	Welcoming the child	I am glad to [meet you today, to get to know you, to get to talk to you]. My name is ________.	WC05	—	—	+	+	+	+
Reinforcement	Reinforcing behavior	You are describing it clearly.	RB01	/	/	+	+	+	+
Emotional Support	Checking on the child's feelings	How are you doing so far?	CF01	/	/	+	+	+	+
Kind encouragement	Legitimating expressions	You can talk about bad things and good things.	LE01	/	/	+	+	+	+
Using Rapport	Emphasizing that you are someone to disclose to	My job is to try to help kids.	SD12	/	/	+	+	+	+
Reinforcement	Thanks and appreciation	Thank you for sharing that with me, it helps me get to know you.	TA04	/	/	+	+	+	+
Reinforcement	Reinforcing behavior	You are giving a lot of details and that's important.	RB03	/	/	+	+	+	+

Kind encouragement	Offering help	Begin talking and I'll help with questions, I am here to help.	OH03	/	/	+	+	+	+
Emotional Support	Echoing emotions	You said you were [sad, you cried, got angry].	CE01	/	+	/	—	+	+
Emotional Support	Empathy	I know it's been a long interview.	EM01	/	—	+	+	/	+
Using Rapport	Emphasizing that you are someone to disclose to	If something happened I'm here to listen to you.	SD01	/	/	+	+	+	+
Using Rapport	Emphasizing that you are someone to disclose to	[Name], here kids can talk about good things and bad things that have happened to them.	SD11	/	/	+	+	+	+
Emotional Support	Exploring Emotions	[Name] is there anything you are concerned about?	EE02	/	/	+	+	+	+
Using Rapport	Expression of care	I care about you.	EC02	/	+	+	/	/	/
Establishing Rapport	Small gestures of good will	Are you comfortable?	GW02	/	/	+	+	/	/
Using Rapport	Emphasizing that you are someone to disclose to	[Name], my job is to listen to children about things that happened to them.	SD08	/	/	/	+	/	+
Establishing Rapport	Expression of personal interest in the child	I am glad I can talk to you today [name].	PI04	+	/	+	+	+	+
Using Rapport	Emphasizing that you are someone to disclose to	You can trust me and tell me if something happened.	SD02	+	+	+	+	+	+
Emotional Support	Exploring Emotions	Tell me more about your [fear, anger].	EE01	+	+	/	—	+	/
Using Rapport	Reflecting on the relationship	You told me a lot about yourself. I feel I know you better and you can tell me more [about things, about both good things and bad things] that have happened to you.	RR02	+	+	/	/	+	/
Kind encouragement	Legitimating expressions	In this office you can say anything.	LE02	+	+	+	/	+	+
Kind encouragement	Warmly emphasizing that the child is the key source of information	I am asking you these questions because I was not there.	WI01	+	+	—	/	/	/

Using Rapport	Emphasizing that you are someone to disclose to	I talk to many kids and they tell me about things that have happened to them.	SD05	+	+	/	/	/	/
Emotional Support	Exploring Emotions	I see you're crying. How come?	EE05	+	+	/	/	/	/
Emotional Support	Exploring Emotions	[Name] what would happen if you told me?	EE09	+	+	—	—	+	/
Emotional Support	Reassurance	Sometimes it helps children when they can talk and do not have to keep a secret.	RA02	+	+	+	+	+	/
Kind encouragement	Encouraging disclosure	[Name], if there is anything you want to tell me, [I want to know/listen, It's important for me to know/listen]	ED02	+	+	/	+	+	+
Emotional Support	Open questions about feelings/thought s during the incident	You said you were [sad, grossed out, wanted to run away]. Tell me more about that.	OI01	+	+	—	—	+	/
Using Rapport	Emphasizing that you are someone to disclose to	[Name], if something has happened to you and you want it to stop, you can tell me about it.	SD03	+	+	/	/	/	+
Emotional Support	Generalization of the child's difficulties	Many children have secrets and I am here to listen.	GD02	+	+	/	/	/	—
Using Rapport	Emphasizing that you are someone to disclose to	[Name], I really want to know when something happens to children. That's what I am here for.	SD10	+	+	+	+	+	+
Kind encouragement	Encouraging disclosure	Please go ahead and tell me.	ED03	+	+	/	/	+	+
Kind encouragement	Expression of confidence/optimism	I think you can describe it well.	EO01	+	+	/	+	/	+
Establishing Rapport	Expression of personal interest in the child	[Name], [I am, people] are worried about you and I want to know if something may have happened to you.	PI09	+	+	/	/	/	/
Kind encouragement	Encouraging non-verbal communication	[Name], go ahead and sit closer to me.	EN02	+	+	—	—	—	—
Using Rapport	Reflecting on the relationship	You told me a lot about yourself, thank you for letting me know. When you talk to me today please go on and tell me about other things that have happened to you.	RR03	+	+	/	/	/	/
Using Rapport	Reflecting on the relationship	You have told me a lot about yourself and I feel I know you better. Now that we know each other better you can share with me.	RR01	+	+	—	—	—	—

Kind encouragement	Encouraging disclosure	It's really important that you tell me if something is happening to you.	ED01	+	+	—	—	/	+
Establishing Rapport	Expression of personal interest in the child	I really want to get to know about you. Today is the first time we have met and it is important for me to know you better.	PI01	+	+	—	/	/	/
Establishing Rapport	Expression of personal interest in the child	I really want to get to know about things that happened to you. Today is the first time we have met and it is important for me to know you better.	PI02	+	+	—	—	/	—
Kind encouragement	Expression of confidence/optimism	I'm sure you could tell me.	EO03	+	+	—	—	—	—
Kind encouragement	Encouraging non-verbal communication	Go ahead and face me, so I can see you.	EN01	+	+	—	—	—	—

Note. + = Rating differed significantly from 0 in the positive direction. — = Rating differed significantly from 0 in the negative direction. / = rating did not differ significantly from 0.

7 Appendix G – Remarks Regarding the Individual Rapport Building and Supportive Techniques (Translated via www.deepl.com Without Further Editing)

7.1 Welcoming (WC05): “I am pleased to see you today/to get to know you/to be able to talk to you. My name is ...”

7.1.1 Group “Abused”

1. I find “I am glad” wrongly formulated, rather the police officer should thank you for coming here, because the topic has very little to do with joy
2. instead of saying I am happy: thank you very much for coming here today to talk to me
3. In itself I feel appreciated, but the statement is strangely formulated (again too offensively personal). Rather: “nice that they came and want to talk to us.
4. I feel treated with respect
5. It is important that I know who I am dealing with.
6. this is not a joyful topic
7. Personal and appreciative, I find good
8. Very friendly
9. I like “being able to talk to them” (I rated them according to this one), the other proposals don't.
10. This fits more into a job interview than into this context.
11. I think “I'm...” much more pleasant than “My name is...”
12. factual
13. i answer everything from my gut feeling, but i believe that the different options given here make a difference
14. The personal contact helps.
15. Creates trust and loosens up the “strictly” believed situation of an interrogation.
16. Also implies that I was only loaded because it is assumed that something happened to me. Better: Thank you for making it possible for you to come here.

7.1.2 Group “Not Abused”

1. too personal
2. Very friendly, neutral formulation and presentation of the policeman.
3. The pressure of expectation generally does not come up with me. I think it's because I've never met policemen like that before.
4. Felt oppressed
5. Good introduction to the conversation: polite and trustworthy.
6. has an inviting effect
7. This reception would make me happy, as the greeting seems to be honest and open and without any intention
8. What should be the reason for him to be happy about it, if he doesn't know me and doesn't know anything about me (hopefully!!!!) yet?
9. in the version “to be able to speak with you”.

7.2 Expression of personal interest (PI01): “I really want to learn more about you. Today is the first time we meet, but it is important that I learn more about you.”

7.2.1 Group “Abused”

1. seems very personal, not professional

2. This sounds more like a statement on a 1st date where someone is interested in you as a person. I would feel a little turned on.
3. I would think he/she only wants to get my information, because it is “important” that he/she learns more about me without giving reasons
4. a little too much emphasis
5. Isn't it about the assaults?
6. Pressure build-up
7. Print
8. Works very much like work/mandatory.
9. Why about me? It's about the deed, about what happened!
10. for me: too brisk and too demanding; rather intimidates
11. why is not the investigation in the foreground
12. I think it is important to put myself in the right perspective and to ask a setting question. Is it a closed or open space?
13. first sentence seems nice and interested at first, but then the pressure of expectation increases (it's important --> you don't want to do anything wrong) and with increasing pressure of expectation the willingness to talk probably decreases
14. The pressure to reveal oneself definitely does not ease the situation. The repetition makes the pressure even worse.
15. see above
16. Implies that something must have happened to me. “Why do I have to testify to that when people think something happened to me anyway?”

7.2.2 Group “Not Abused”

7.2.3 “it is important” puts more pressure on me

1. I have the feeling that the statements repeat themselves.
2. Possibly too demanding wording
3. for what? if you are a victim with a guilt theme, you might think that you are being made a rope out of what you are told
4. Although it is clear that it is difficult with the 1st contact is demanded
5. i don't want to be asked about me, what does this have to do with it? Now I'm being careful...
6. I personally find this question too demanding and would feel pressured by it. What exactly would the policeman want to know now? Does he have a specific intention?
7. I ask myself why it is about me as a person and not about the events of that time and want to separate myself.
8. More clearly pressure cannot be built up and borders ignored, thus already the existence of personal borders is ignored in this sentence!
9. I find too close
10. By repeating the set, slight pressure is applied.

7.3 Expression of personal interest (PI02): “I really want to know about the things that happened to you. Today is the first time we meet, but it is important that I learn more about you.”

7.3.1 Group “Abused”

1. Why “.... learn about you.”? ... probably rather about the assaults and incidents and the behavior of the former teacher. .....then the question would be good.
2. Why do I have to collect so much personal information about myself and get to know me during the survey? It's all about what the teacher has done to you and others.
3. exerts pressure, not good
4. This puts a lot of pressure on me. Maybe rather “I would like to know about the things ... but it would help me a lot if I knew more about you.”
5. About me or the act?
6. I would probably be less willing to answer open questions. However, due to the emphasis on importance, I would respond to closed questions (although probably hesitantly)
7. Too brisk and too demanding
8. ok
9. I feel a strong pressure to say something.
10. too demanding
11. It is not important for you to learn more about me as a person. What does my favorite color or my favorite food do to matter? It is potentially about finding evidence or counter evidence for a crime. Limit yourself to that.

7.3.2 Group “Not Abused”

1. Well-being is reduced because it seems to me like a strong invasion of privacy and therefore willingness to talk is lower. pressure of expectation increases, since it is again implied that I have been the victim of a rape, which does not correspond to reality
2. I feel under pressure
3. That explains the situation well.
4. too early
5. This puts pressure because it emphasizes the fact that you don't know each other and yet you want me to tell you about potentially intimate things
6. Too demanding and researchful
7. Pressure is being built up, but I am left in the dark as to why this is so urgent.
8. I personally find this question very challenging, especially since nothing happened to me in my role. I would feel pressured
9. See note question 8
10. I would like to be able to report at my own pace.
11. Why does he WANT to know? Own speed, personal limits, obviously do not interest this person. Could well be a perpetrator himself, that's the basis for it. I would certainly not talk to him. That's really crass. I hope the reality is less horrible on average.
12. more about the possible events / about the suspect even better than “about me

7.4 Expression of personal interest (PI03): “Well, dear [your name], I would like to get to know you better. Why don't you tell me about things you like to do?”

7.4.1 Group “Abused”

1. I can distract and fill the time with other things
2. Too intimate & unsuitable in this situation.
3. is too private for me, away from the topic
4. Better: If you like, you can tell me about things you like to do
5. creepy; not the business of the officials --> suggests an attempt to build up a friendly relationship, but this will not/may not happen at all.
6. Must be done in both directions.
7. Has no place in such a survey
8. This is none of his business
9. encroaching
10. Why ? Careful. the explanation is missing
11. Very good, because I am not reduced to the experience!
12. But in this situation I would not like to
13. It is clear why the conversation is taking place. There I find it inappropriate to talk about things I “like to do”, or I simply find the wording inappropriate. It would be okay to talk about current job or something similar.
14. Takes the pressure/fear out and loosens the atmosphere
15. This has nothing to do with the context and would bother me.
16. Can have a very negative effect on people with a disturbed relationship with the police.
17. What is the question ? What I like to do does not matter in this situation!
18. Why does he need to know that?
19. i wonder why the policeman wants to get to know me better? Does he not believe me?
20. I would feel screwed. I'm here to make a statement and not to have a coffee party and be addressed as dear XY by a stranger. This is cross-border.
21. “Sweet” is inappropriate here. How does he know that I am sweet?

7.4.2 Group “Not Abused”

1. I find this question strange, because it is not target-oriented and rather less loosening or relaxing
2. the expression love/r would come too close to me, especially in this context
3. encroaching
4. Neutral question
5. What does this have to do with the matter? I think it's private.
6. inappropriate question
7. Why should I talk about it now. It has nothing to do with the incident.
8. i would ask myself what this is important for, as a person with a great need for control, i would like the officer to say that it is about getting a little warm/comfortable with the situation, the information about my hobbies is not really relevant
9. unsuitable

10. do not address with “love...” under any circumstances, crossing a personal limit, very inappropriate situation for small talk
11. Am I with the therapist or with the police - irritation
12. I don't know why this should be important, but why not? That's a nice question.
13. Side issues
14. Why does a civil servant need to know when it's not about me, but about the teacher?
15. Creepy. Too close! Encroaching!
16. Irrelevant and border violating question
17. how is this relevant?
18. “love”????!!! Cross-border! Disrespectful. Once again: he should not “get to know” me. And what is the point of telling things I like to do?
19. Could be okay as an introduction, find it with “love”, this private level, wanting to get to know each other too unprofessional
20. The phrase “I would like to get to know you better” sounds wrong in this context. But it is positive to talk about nice things.

7.5 Expression of personal interest (PI09): “[Your name], [I am/persons are] concerned about you and I would like to know if something has happened to you.”

7.5.1 Group “Abused”

1. Suggests that you can burden other people by telling them about the incident
2. too suggestive
3. This question seems very private. Actually it is more about the fact that I could help to convict my former teacher and protect other children from him. Of course it is important if something has happened to him, but I am in a police interrogation, not in a therapy session.
4. I am still too suspicious and this introduction does not change my attitude
5. puts under pressure, I am not responsible for the others
6. I realize that others care about me.
7. A feeling of pressure is created.
8. Feelings of guilt, why is he worried
9. I wouldn't want to worry anyone, although I have not actively contributed to it. So to take away the worry from the other person I could say that I am fine and nothing has happened.
10. I don't want to burden anyone with it, that's what kept me from telling anyone! This statement would rather keep me from telling other things.
11. It seems a bit fake when a stranger says he is worried about you.
12. If it is a first question, it would already go too far for me.
13. “...concerned about you and your former classmates.” gives the impression of not being alone and exposed. Some people also find it easier to be strong for others (victims) than for themselves.

7.5.2 Group “Not Abused”

1. too personal

2. Anxiety increases well-being and willingness to talk. The phrase “whether something might have happened to them” slightly increases the pressure of expectation. However no such demanding formulation, therefore only in small measure.
3. I find it excessive to speak of “worried” when the police officer does not know anything about my situation yet.
4. If it came across authentically, I would feel picked up.
5. The fact that a police officer who is a stranger to me is immediately “worried” would be too much/too close for me.
6. A positive start to the conversation (shows sympathy and empathy). However, it may be that too personal a reference to the beginning is rather counterproductive. After all, at the beginning, trust and bonds are rather weak: Therefore, too prompt a personal reference could make you feel insecure.
7. you do not necessarily want to burden others, even if it is nice when others think of you
8. Too close, is a stranger, why does he worry about me, his job is to investigate, please be more neutral
9. Helmet on, off into the trench, ready for defense: the question doesn't even go
10. That the policeman expresses concern and apparently wants to care would make me feel good, even if nothing happened to me in my role
11. unrealistic that they are worried
12. If other people are worried, ok. If he is- pretentious
13. I do not believe the concern, does not seem authentic, inhibited.
14. The question sounds very emotional (people are concerned) and strongly suggests that the teacher has indeed been assaulted.
15. Who is concerned? Why should you be worried about me? We don't even know each other.

7.6 Expression of personal interest (PI04): “I am glad that I can speak with you today, [your name].”

7.6.1 Group “Abused”

1. Very personal, therefore pleasant
2. Glad I find inappropriate
3. The I-message rather triggers trust in me
4. exerts pressure, assault, focus on the feelings of the policeman, I feel bad, it is not about how the policeman feels!
5. “glad”?
6. Seems very much like a professional meeting.
7. The date was not my wish - so he/she can be as happy as he/she wants and I would not care.
8. okay
9. Is it still a friendly police officer?
10. “I'm glad you came.” is enough, you don't have to pretend to be friends.

7.6.2 Group "Not Abused"

1. it sounds as if the interviewer is personally interested in the results, if he would say "nice that ..." it would be more neutral than "I am glad ...".
2. unnecessary phrase, better come straight to the point
3. Incredible
4. I wonder where the joy of the interviewer comes from. I would like to hear a phrase such as " Thank you for making it possible, or thank you for allowing us to come together today to talk about...".
5. I am not the person to contact if the interviewer needs to talk! Totally out of place and inappropriate.

7.7 Small gestures of goodwill (GW01): "Are you cold? Would you like us to take a little break?"

7.7.1 Group "Abused"

1. My needs are taken care of, I feel better
2. it is dealt with my sensitivities
3. he pays attention to me. Break is good
4. I feel well taken care of, because my counterpart does not only go off his list, but pays attention to me and my signals
5. Rather quickly go away again...
6. I would be happy if people would inquire about my condition in between. Besides, the offered break gives me the impression that the policeman is patient and does not want to get the information from me as fast as possible.
7. empathetic
8. first question too intrusive (physical), second question pleasant
9. This is a concrete offer that relieves me.
10. Adults can usually take care of their own body temperature. The question about the break makes sense.
11. The official will take my well-being into account and leave the decision about the continuation of the interrogation to me.

7.7.2 Group "Not Abused"

1. 2 questions at once are confusing
2. Readiness to speak after a break but not at the moment
3. Concern for my well-being in a very pleasant way increases the well-being and lowers the pressure of expectation in this situation
4. This signals that there is no time pressure. That relaxes me.
5. in itself a very friendly, attentive question, but under certain circumstances you may feel "weak" (not able to guarantee a smooth flow of the survey)
6. The possibility of a break is important, it also makes the conversation more everyday in some respects (reduces pressure of expectation)

7. I would rather ask if the person needs something, a break or a warm drink etc. Otherwise the answer can be “no”, but actually there is
8. Takes speed out, gives me time to take care of myself
9. I am glad that my condition is important, that the policeman cares about me
10. My personal well-being is taken into consideration here
11. If I don't say or tell by signs that I am cold, then such a question is annoying. It should be pointed out in advance that I can ask for a break at any time. It is ok to be reminded of this, otherwise I find such a question unpleasant, unless it is clear that something has touched me very much.
12. At the beginning of the conversation it is strange to pause and ask if I am cold. Irritated.

7.8 Small gestures of goodwill (GW02): “Do you feel comfortable?”

7.8.1 Group “Abused”

1. Maybe even: “what can I do to make the situation more pleasant for them?
2. stupid question - nobody feels comfortable in an interrogation situation Better to ask openly: how do you feel?
3. In this situation certainly not
4. This question sounds more like sympathy
5. I feel compelled to say “yes” or make similar gestures.
6. What kind of question is that?
7. How should I feel comfortable
8. Formulation and suggestive question. Of course I do not feel comfortable after such a conversation. Better would be an open question: “how do you feel now?
9. The closed character of the question makes it difficult to negate. With an open question it would be easier to report negative feelings.
10. What kind of question in this situation?!
11. What a flat question !
12. This question is like salt in the wound, little empathic after such a topic! I feel anger germinating inside me.
13. This is an absurd question
14. What kind of question is that? Who feels good after reliving his childhood trauma?!
15. This question seems too suggestive. The official almost expects me to answer with “yes”.
16. Somewhat better feeling than in the previous question.

7.8.2 Group “Not Abused”

1. also a good situation to show that you don't necessarily feel comfortable in your current position (but still make a statement to move the research forward)
2. In my opinion, puts the focus on becoming more aware if you feel uncomfortable.
3. Suggestive question I do not like
4. what if he doesn't? What am I supposed to say then, with the convention of “how are you?” “good” in our society? Better to ask openly.
5. nobody feels comfortable talking about sexual violence
6. more openly asked would be nicer - might sound like irony Question 57 sounds better

7. A very research question. Do I have to answer with yes or no?
8. Feel good? For such topics??????
9. when? in general?
10. Depending upon cause good attentive question, perhaps one is with the narration however in the happening in and away from the present well-being, so that this demand inappropriately placed brings one out of the concept
11. I would never admit it if it wasn't.

7.9 Small gestures of goodwill (GW03): “What can I do to make you feel more comfortable?”

7.9.1 Group “Abused”

1. given as a note before - sorry :)
2. I find it more pleasant to be asked whether one can do something that leads to more well-being.
3. It is questionable whether someone with an unknown policeman, in an unfamiliar environment, is so reflected and can answer or dares to answer.
4. possibly add a direct suggestion (e.g. offer tea)
5. In such a situation, there is little that could increase well-being
6. It's good that I'm involved and asked, but you just can't feel comfortable in such a situation!
7. positive effect
8. Good question
9. Gives me the feeling that the policeman cares about me and perceives me as a person with feelings.
10. So no policeman asks
11. Concrete offers for breaks or something to drink would be better, because with this setting I wouldn't know what is possible.
12. * to make you feel more comfortable.
13. Although he responds to my wishes / requests, he also expects me to finally talk.

7.9.2 Group “Not Abused”

1. Well-being somewhat reduced as it is still a formal police interview and the relationship with the interviewer should not be too personal.
2. It depends on the time of the conversation when this statement appears: In the beginning it would unsettle me, later I would probably find it helpful.
3. Good question!
4. shows more readiness than the question “Can I do anything to make you feel better?
5. good, because open questions and I can decide for myself
6. Here it is easier for me to express something that makes me feel uncomfortable
7. Polite question, but seems not very empathetic
8. That builds up pressure, according to the motto “now why not relax? I would like the interviewer to say something like “what do you need”.
9. Maybe nothing, after all the attempt is well meant. It would be more respectful . “Is there anything I can do to help.....”
10. Is it obvious that I feel uncomfortable? Then I feel caught now and doubly uncomfortable.

7.10 Small gestures of goodwill (GW07): “Are you comfortable?”

7.10.1 Group “Abused”

1. Inappropriate and not authentic
2. always a good start
3. Does not matter
4. That sounds kind of rude, although I'm sure that with the right intonation it can sound friendly
5. Unsuitable
6. this is always an important start
7. maybe already too detailed, a general “Do you feel well?” maybe better
8. I would need an offer of change here if I am not sitting comfortably. Here I lack the option, otherwise I would not answer honestly.
9. What if not?
10. He wants me to do as well as possible in this difficult situation.
11. first gut feeling: strange question...

7.10.2 Group “Not Abused”

1. I find the question unsuitable
2. I find that a bit funny. Sounds like a movie. But it would relax me a little bit.
3. Too much attention
4. -> Well-being important for readiness to talk
5. in such a situation I would not like to make small talk
6. it is also looked after my condition
7. I personally find this phrase very friendly, because you feel so well taken care of
8. Interesting. Despite the pressure of expectation (very discreet) well-being, because it reminds me to feel how I am doing, thereby also talking more openly ....
9. the question seems very artificial. it can be assumed that you will find acceptable seating when you are invited for a questioning. all in all, the question seems to me to be popular psychological and artificial/not sincere
10. not necessary question, rather offer drinking or similar.

7.11 Small gestures of goodwill (GW08): “Can I do anything to make you feel better?”

7.11.1 Group “Abused”

1. 75. better than 74. question
2. Better but similar to 24)
3. More professionally formulated than the previous question.
4. I find this question better than the question “What can I do to make you feel better?
5. s. Question 14
6. upper question is more concrete
7. I found the previous wording better (“What can I do to make you feel better”) because it sounds more motivated to really do something. With this question (“Can I ...”) I feel I should answer with “no, thank you”.

8. Would trigger the same reaction in me as when I ask “What can I do to make you feel better”.
9. Even if there was something, at least in most cases I would say no. I find the phrase “What can I do...” much better because it immediately makes you realize that there is something.
10. already better
11. see 59.
12. Better than 54.
13. He responds to my wishes / requests without creating additional pressure of expectation.
14. feels better

7.11.2 Group “Not Abused”

1. slightly more pleasant formulation than the first one
2. I like it better than statement 19.
3. expression implies that I might feel uncomfortable because something unpleasant has happened. Therefore increased pressure of expectation. Well-being slightly increased because question seems less intrusive.
4. See 44.
5. He tries hard. Not as inviting as the question before.
6. Personally, I find this question very empathetic and it would make me feel that the policeman cares
7. Did I say that I feel uncomfortable?
8. YES. exactly. This is how respect works. Whew. Finally time
9. creates a relaxed atmosphere

7.12 Reinforcing behavior (RB01): “You describe it clearly.”

7.12.1 Group “Abused”

1. I am not quite clear whether this statement is meant to praise me or what is meant by it.
2. I cannot really place this statement
3. Positive gain
4. This can be either criticism or praise. I can't judge it that way.
5. low-dose recognition
6. Include a praise. “This is good, we need details to...”
7. The official encourages me to go on like this and keep on telling.

7.12.2 Group “Not Abused”

1. Positive feedback motivates
2. Am I passing sentence here?
3. I personally would not want to talk at all with this demanding statement.
4. At least it's worth the effort! That feels good.
5. What is this confirmation for? All-inclusive?

7.13 Reinforcing behavior (RB03): “You have given me many details, which is very important.”

7.13.1 Group “Abused”

1. Recognition for my performance, which I can't assess well myself. I feel rather incapable
2. ...mentioned many details, for which I would like to thank you, because it helps me to better understand what happened
3. The feeling I have done it seemingly right
4. The feedback about “how well you do it” is very important to me. Well done!
5. There might be some pressure to go into more detail and maybe I'm overwhelmed because I don't remember more or I'm not sure if certain things really happened that way.
6. ??
7. Appreciation
8. I have done that well. I still remember the experience well.

7.13.2 Group “Not Abused”

1. Emphasize that it is right and important to talk about it.
2. good to know which information is important
3. Works like a praise, motivates
4. I think it would be more appropriate for the interviewer to say something like “thank you for describing xyz in such detail”.
5. Important for what? It would be nice if he added that. Otherwise it would be a relief, obviously I told enough so that the facts are sufficient.

7.14 Reinforcing behavior (RB04): “You really help me a lot to understand you. Thank you!”

7.14.1 Group “Abused”

1. Am I the problem here?? is going through my mind.
2. It is not about understanding me, but what happened back then
3. I feel appreciation for myself, that makes me less suspicious
4. empathic and appreciative
5. Very nice reinforcement
6. okay
7. Do not perceive yourself as a victim.

7.14.2 Group “Not Abused”

1. I find this attentive and nice.
2. i would then also ask myself why this is about understanding me, i thought it was about my teacher.
3. Depends on the context, looks a bit thickly applied
4. In my opinion, this is where the emphasis is important. Could give me a feeling of support, but also a feeling of pressure

5. Appreciation is also good
6. Triggers alertness and irritation in any case. Why should I HELP someone else to UNDERSTAND ME:
7. to help me understand sounds strange with the thank you for that.

7.15 Reinforcing behavior (RB05): “You have corrected me, which is very important.”

7.15.1 Group “Abused”

1. I am accepted as an expert, very important.
2. I feel taken seriously and not as if words are put into my mouth
3. I could still take that as an accusation, I can't judge what he really thinks
4. I can keep control, that is important
5. A very good sentence, because I usually do not dare to correct others
6. Conveys the feeling that the information is received exactly as I present it and that it is not interpreted differently.
7. I can't do anything with this statement in terms of content and don't understand it.
8. The correct representation would be important to me.
9. Very good. I did it right and this is expected of me.

7.15.2 Group “Not Abused”

1. comes here very strongly on the tone is, if it comes credibly, it can be good
2. I think that's good, because it shows that the person leading the conversation is unbiased.
3. funny
4. I am allowed to correct without the other person being angry - gives security
5. can actively design
6. So the policeman is really interested in my story, even if nothing happened.
7. See note question 14
8. Insight is good
9. Maybe a thank you in addition, that would increase the well-being
10. You can tell that the policeman wants to know what I have to say and does not want to confirm his own assumptions.

7.16 Reinforcing behavior (RB06): “I understand what you want to tell me.”

7.16.1 Group “Abused”

1. “I understand.” would be better
2. Sometimes it is difficult to put bad experiences into words, I feel understood
3. Understanding
4. As not concerning nobody can understand that probably - not so good statement on the part of the interviewer
5. ??
6. again thought, if male policeman can understand me...
7. one feels understood --> self-efficacy

8. “I think I understand...” Declaring that a fact is presumptuous.
9. The official can understand my remarks.

7.16.2 Group “Not Abused”

1. Understanding shows that I am well listened to and not just paying attention to the details that prove the teacher's guilt.
2. I don't think you should say something like that in this professional situation, because after such a short time of speaking you can't “understand” the situation of a person.
3. Very important: To be responsive to the person and what they tell. Make it clear that what is said is taken seriously and understood (-> positive reinforcement)
4. does he really?
5. Why do I have doubts now?
6. And that would be? Personally, I find this a bold statement
7. Implies that person knows more about me than I do
8. Pretentious. How does he know that, when I obviously haven't even said it directly? At least before “I think, ...” and afterwards the question whether it is true.
9. It is better to repeat the content or have it summarized and confirmed (e.g. nod) or ask specifically that you understood it to be correct. Thus it seems incoherent, possibly not applicable.
10. The policeman conveys understanding. This directly increases the willingness to speak.

7.17 Thanks and appreciation (TA01): “I would like to thank you for your help.”

7.17.1 Group “Abused”

1. Pleasant recognition again
2. More formal than “I appreciate you talking to me” and therefore not so good for the well-being.
3. If this sentence is at the end of the conversation, you may be afraid that you have forgotten something important and you may not feel so good. Then there would be something like “If you think of anything else that could be important to us, please feel free to call us again.
4. okay
5. equal interlocutor, you yourself less in the “victim role
6. I was able to help with the investigation of the case and was free to decide whether and to what extent I would like to report about my experiences.

7.17.2 Group “Not Abused”

1. More appropriate than the previous remark.
2. It's nice that the conversation is finally over. Nice to be thanked
3. like 11
4. Very nice farewell words.

7.18 Thanks and appreciation (TA02): “I really appreciate you talking to me about this.”

7.18.1 Group “Abused”

1. Appreciation, being seen, being taken seriously
2. I find this respectful interaction good and I feel “valuable”.
3. very appreciative
4. I could make him happy that I talked about it and I feel relieved.

7.18.2 Group “Not Abused”

1. Appreciation of my information increases my well-being and reduces the pressure of expectations
2. personal formulation and appreciation creates closeness
3. it's good that here the talking and not the content is praised, at the same time it sounds again like it is important to the person personally, if/that I tell, do I want that from an official? He should not be burdened or personally affected
4. A bit too much - thank you for the interview would be enough
5. Appreciation is always good, might even be tempted to say things I have held back so far
6. The conversation seems to be over. Sounds friendly
7. Very nice farewell words.

7.19 Thanks and appreciation (TA03): “Thank you for trying to remember everything and for telling me what happened. Thank you for telling me.”

7.19.1 Group “Abused”

1. Appreciation reduces the pressure of expectations to a minimum.
2. the word strive sounds as if I had failed
3. Gives me the feeling that I can help
4. I feel valued
5. I am taken seriously
6. I would be relieved that I am not being condemned and that my testimony is appreciated.
7. comes across well,
8. Do not repeat yourself so often, you are not stupid towards her.

7.19.2 Group “Not Abused”

1. Even if I have reported the teacher's innocence, my information is valued.
2. This feels very picking up and understanding in the performance. As if my counterpart was very quick-witted.
3. Very appreciative
4. Feels good!

7.20 Thanks and appreciation (TA04): "Thank you for sharing this with me, it helps me to get to know you better."

7.20.1 Group "Abused"

1. "this" again referring to sexual violence? - through this one gets to know someone better only to a limited extent, perhaps feels reduced to that, moreover the police officer does not have to get to know me better
2. I don't want to get to know you better, but I am here to talk about a very unpleasant topic
3. ... why get to know me better? "Thank you for sharing this with me, so we know more about what really happened. You help us a lot", I would find better.
4. Again: it should not be about getting to know me better, but about catching the culprit
5. I do not want you to get to know me
6. In itself good, but it sounds a bit like this experience is a big part of my personality, which I don't want to see like that.
7. Why does he want to "get to know me"?
8. getting to know each other is not necessary
9. this is a bit exaggerated
10. it is actually "pretended" that it is about getting to know each other better, it is already about the case, besides, one has usually also no interest to "get to know" the policeman better
11. Real interest

7.20.2 Group "Not Abused"

1. Since this is not a therapy session, but a one-time session, I find it too personal and daunting.
2. I do not want the foreign policeman to know me well.
3. This triggers something strange in me. Distrust or something. I don't even want to meet him.
4. what does he need to know me for?
5. It's nice to say that he gets to know me better that way, but why does he want to?
6. Why would I want him to KNOW ME!? across borders!
7. I am now automatically suspected of lying when the interviewer "has to get to know me first?"/ this sentence confuses me
8. getting to know each other is strange

7.21 Thanks and appreciation (TA05): "I am pleased that I can learn more about you."

7.21.1 Group "Abused"

1. Works like real interest
2. find "rejoice" in this scenario simply unsuitable
3. I am here to comment on the topic/to get involved and nothing else
4. happy sounds wrong in this context
5. this statement has a very mechanical effect. One suspects that rapport is to be built up and fixed phrases are used for this.
6. In connection with the above question positive, otherwise negative
7. am not here to drink coffee

8. the “may” gives me the feeling of being able to decide for myself what I tell
9. This sentence fits in a job interview but not in such a situation.
10. Cliche
11. I find these statements somehow strange
12. “Glad” may be a bit exaggerated, but it is not immediately about unpleasant topics.
13. see 39.
14. He is really interested in my person.
15. Joy is probably not appropriate.

7.21.2 Group “Not Abused”

1. I have the feeling that the policeman is interested in my situation.
2. I do not want to make friends with the policeman. He is not here to learn more about me, but about the case. Sounds a bit hypocritical.
3. huh? he is doing a job here and this is not dating - I think this sounds so personal and not appropriate to the situation
4. I am not here to bring them joy, am I? Irritating
5. Too close. Especially in this setting this feels very obtrusive.
6. What is this? Have you ever heard of privacy?
7. looks somewhat artificial
8. Does not sound serious
9. too unprofessional, personal
10. Sounds too personal
11. Too polite, so it seems artificial.

7.22 Respect fot the child's decision (RD01): “It is entirely your decision whether you want to tell me about it or not and I will accept your decision/. It's my job to let you decide.”

7.22.1 Group “Abused”

1. It is my job to let them decide is difficult to understand and gives me pressure to bring about the enlightenment
2. It is said that the decision is mine, but there is also a certain expectation of me. Maybe I am only told that I have the power of decision, so that I feel more comfortable and then decide for the “right” one. (From the point of view of the police, the right thing here would be to make a statement against the accused by reporting what happened to me).
3. This statement is very important & should be made right at the beginning.
4. Being told that it is the job does not make a very sensitive impression.
5. I would feel a little bit dropped, since the policeman refers to his “job” in this way. Better for me would be the addition that it would mean a lot to him if I managed to tell him about it.
6. 1st set good, second set not so good
7. very good to reduce the pressure
8. “...and I will accept your decision. Because it's my job to let you decide” can be omitted. Then more positive.
9. I feel the pressure taken from me

10. Voluntariness increases my well-being
11. the sentence “it is my job” is not possible
12. I would probably leave out the “it's my job” part because it makes the relationship less familiar. Otherwise a very good statement
13. It would make it easier for me to know that I don't have to say anything I don't want to, so I would be much more willing to say something on my own
14. Unfortunately, the last sentence does not fit with the rest.
15. The first part of the question is good, after that it's hard to let up. It doesn't matter what his job is. He accepts my decision and that is good. It would be better to create a pleasant atmosphere, that's all too “cool”.
16. Probably the intention is that I become more relaxed. But I would put pressure on myself to talk through the whole “your decision”.
17. kind of funny: Police must clarify facts
18. As a person I am taken very seriously by it!
19. omit the last sentence, this one sounds as if you would do it only because of that --> it should be self-evident that you are not forced to do anything
20. I remain self-determined.
21. Repeatedly saying it is my decision is what I imagine to be stress-increasing. “Can you tell me about it” would be much more neutral and expresses my choice as well.
22. “Job” sounds pejorative. The official is not really interested in me. He actually doesn't want to let me decide for myself, but has to.
23. Put me under pressure!

7.22.2 Group “Not Abused”

1. “it's my job” statements always seem slightly instructive and somehow strange and not necessary or goal-oriented
2. Better without the last sentence
3. That sounds very clarified and distant to me (“It's my job”)
4. That is to be explained to me. But it can also be because I slowly have an overload. I think there are many questions/statements. Slowly I feel like I'm in a museum when I've looked at fifty paintings and can no longer say which one touches me.
5. Good question!
6. I would feel more comfortable without the 2nd sentence
7. Answering depends very much on your own previous knowledge.
8. Right! An answer or the will to talk about it cannot be forced anyway. To force this with all means will rather have the opposite effect. Therefore leave the decision to the person and express this clearly.
9. that it is his job to let me decide is simply not true. I find this sentence funny, the rest is good
10. omit the last sentence
11. I get room for maneuver, may decide
12. It does not restrict me, which I find good

13. A somewhat casually formulated, there could be indifference behind it. If the behavior otherwise shows that this is not so, then it is very respectful.
14. the second movement seems clumsy/unprofessional
15. It is unusual that people are not convinced to testify for the benefit of the victims. For me it is also questionable whether this is the way to decide against it (when quoting for questioning etc.)

7.23 Reflecting on the relationship (RR01): "You have told me a lot about yourself and I feel that I know you better now. Now that we know each other better, you can tell me about the event."

7.23.1 Group "Abused"

1. I don't know him, I feel a little bit lied to/cheated/tricked
2. I find it inappropriate to take general information as an obvious prerequisite for the other person to reveal more intimate details about himself.
3. As a policeman, I would rather ask if the victim feels ready to talk about the event and not simply state that the victim should be ready now.
4. Aha, you're the checker!
5. ...if you feel like it, you can tell me about what happened
6. Feeling: Person only wants to get to know me to get my information and is not really interested in me.
7. More important than the policeman knowing the victim (me) would be that I get to know him better, so that trust can develop. The policeman should tell more about himself. For example how long he has been dealing with this topic. Why he, as a man, stands up for or against it.
8. It is about a very bad and private experience. I feel oppressed. Formulation unprofessional.
9. Too confidential
10. that's arrogant, he doesn't know me at all, I give him the little finger and he wants the whole hand, that was just strategy, now he puts pressure on me, now I don't say anything
11. he decides here
12. The second sentence is very demanding and more like checking off a checklist. I would like it better if it was formulated like this "I have the feeling I know you better now. Well, do you feel ready to tell me about the event?"
13. I don't want him to know me. I do not know him either
14. Frightens me rather. I would put myself under pressure, which would make me nervous.
15. The first set is good, but the last set destroys everything again.
16. what is this ? The event is in the center of the questioning, not just getting to know each other
17. I feel adequately introduced to the topic and understood by the question.
18. he determines about me (in general I would of course prefer to talk to a woman)
19. I am not reading a question here but a challenge. That is where pressure arises.
20. It is presumptuous to claim to know someone after one or a few conversations. Refrain from hypocrisy and instead be honest and authentic, this will encourage him to do the same.
21. The official thinks he knows me already? I hardly know him. Was that just an act of his interest in me? Actually he only wants my statement about the oppressive experience.

7.23.2 Group “Not Abused”

1. this wording seems rather strange in view of the fact that you only had a (short) conversation before. Just if no abuse took place the formulation is rather insinuated/unappropriate
2. Exchange increases well-being, because anonymous barrier is reduced, but expectation pressure increases, because actually nothing bad has happened that I could report. Readiness to talk is high, because I do not have to report what is unpleasant or has to be concealed.
3. I don't understand what I should have told them.
4. In relation to the event, this corresponds more to the expectations of the conversation and can therefore increase willingness to speak.
5. De facto “we” do not know each other better. He knows something about me, but not I about him.
6. If he “goes to so much trouble” I have to deliver something now? He thinks he already knows me?
7. For me personally, this question would be much too demanding, it would intimidate me, even if nothing happened. If something had happened, I would probably close down,
8. Again, I would feel pressured because I could not confirm any accusations. The more often I would be asked such things, the stronger this feeling would increase.
9. I find it rather unpleasant to have “told a lot about myself”, since I am actually there to talk about an event. If I would rather learn more about my counterpart...
10. “because WE know US better”- again so assaulting. that is terrible! Why are there so few respectful questions? And he is not even supposed to “know me better”!
11. if there was no event?
12. For me personally, knowing better does not have a calming effect. To trust the policeman, I would base my decision on something else: his competence to take time, to really want to understand me (not: to know me personally, to be a friend, etc.)
13. Sounds too personal.

7.24 Reflecting on the relationship (RR02): “You have told me a lot about yourself. I feel I know you better now and you can tell me more [about things, good or bad] that happened to you.”

7.24.1 Group “Abused”

1. Aim clearly for the bad things. Something like that would put me under pressure to tell about my negative experiences, which I actually wanted to suppress.
2. I know it now better suggests an intimacy that is not yet there.
3. manipulative
4. I want to decide for myself when I know someone well enough to tell them about something. This should not determine my counterpart.
5. empty phrase. Sounds empty
6. Same as question before: Emphasis should be placed on the victim getting to know the interviewer better in order to build trust and then be able to talk about the incidents more effectively. When it comes to the policeman emphasizing that it is important for him to get to know the victim, I ask myself why. ... To want to check the credibility? This would not be very conducive to trust.

7. It is about my statement, not about getting to know me.
8. Why tell even more, have the feeling it is always drilled further
9. increases the pressure and takes the decision
10. Very nice formulation. However very unspecific and I don't think I would know immediately what to say
11. Why do you know me better? Why can I tell you more. I want to decide whether I tell something and this depends on my impression of the person interviewing.
12. Why should he know me
13. “I have the feeling I know you better now” would encourage sympathy for the policeman.
14. this is somehow too much exaggerated
15. I personally am more of a fan of “getting straight to the point” and not “talking through a flower” (I feel rather ridiculous/ like a child because I know why I'm here), but not everyone is like that and I might be glad if something really happened to me
16. I cannot imagine that these statements from a police officer would be helpful for me or that I would feel more connected to him
17. If a conversation is already in progress and confidence is conveyed, things are a bit easier.
18. You don't know a person after a conversation, you shouldn't pretend you do. But you can certainly understand someone better and this may also be said
19. We are getting closer to each other. He understands my situation.

7.24.2 Group “Not Abused”

1. after a conversation you do not know a person better...
2. Neutral formulation that does not imply that I was a victim of the suspect.
3. I would not like the policeman to think that he knows me better after a short conversation.
4. Hmm... I have the feeling that I don't have that much to tell, because my fictional teacher only hugged me or stroked me over the head. I think I would probably have told everything by now if the official had said: “You've told me a lot about yourself.
5. Possibly formulated too generally. The person's expectations regarding the interview will most likely be limited to the incident with the school teacher. This question is more likely to be expected during a therapy session and could be unsettling. Perhaps relate the question more specifically to the situation at hand.
6. too close approximation
7. creates pressure - had to listen to a lot, now I have to deliver
8. manipulative
9. Here I would probably talk personally, but since nothing happened to my role, I would probably react annoyed to this long execution
10. Because HE knows me better I should be able to tell ME more? The prerequisite is probably the other way around... that I would have got to know him better, to find out whether I am in good hands.
11. Difficult to know if the police have this context to build the relationship in this way?
12. The word “happened” is slightly negative. By saying “good or bad things” in front of it, this is somewhat weakened.

7.25 Reflecting on the relationship (RR03): “You have told me a lot about yourself, thank you for letting me know these things. If you talk to me now, tell me about other things that have happened to you.”

7.25.1 Group “Abused”

1. strange formulation
2. Statement? do not quite understand what this remark is meant to say
3. Better: “If you talk to me today, I would be happy if you could tell me more about the things that happened to you.
4. The second movement is unspecifically suggestive. If the first movement stood alone, I would find it much better.
5. somewhat unclear, at what time this statement is made, before the conversation/ after the conversation/ during the conversation?
6. Beautifully professionally formulated
7. The first part of the sentence is very appreciative, but then comes the next request, which triggers pressure
8. Print and no explanation
9. It does not seem very human/natural, but somehow mechanical and professional
10. Relief at having talked.
11. That sounds like a command. It would be better formulated in a question.
12. Appreciation of the efforts made so far.
13. *of more things. Leave the repetitions, your counterpart is not difficult to understand. “What else can you tell me about the topic/from that time?”

7.25.2 Group “Not Abused”

1. A question instead of a statement would increase my willingness to speak.
2. I don't want to reveal too much about myself and have nothing to report about a rape.
3. I feel under pressure to tell something relevant. Maybe there is nothing relevant left.
4. I have the feeling - as I said before - that I don't have that much to tell.
5. If you talk to me today... But I'm talking to him now...
6. this is suggestive
7. The 1. part is appreciative, the 2. part makes some pressure - as a question inviting
8. Nothing happened to my role. Why doesn't he want to know more? What more can I tell him? What does he want to hear?
9. Question formulated comically
10. Extreme expectation. Only if it is really my need, I will tell you more.
11. is that an invitation?
12. suggests a little that “other things” have happened, only when it was predetermined. And the “tell me today” is strangely demanding

7.26 Expression of care (EC01): “I am here for you.”

7.26.1 Group “Abused”

1. Sounds very personal for a foreign police officer.
2. what about after the interview?
3. again suggests false proximity. After the interrogation the officer is no longer there for you. Very unprofessional, manipulative.
4. If this is really the case: the same contact person for the entire time, with good accessibility: very good. Then perhaps you could also say in what way.
5. As already mentioned, the policeman should not serve as a therapist. This statement also seems extremely unprofessional.
6. What that means. is not really convincing to me
7. depends on credibility, sympathy....
8. Skepticism, because we hardly know each other and no confidence-inspiring question has been asked yet.
9. But I do not want
10. Gives me the feeling of getting support when I need it
11. Inappropriate slogan and too sweeping or credible
12. that comes across exaggerated
13. Too personal - a friend or partner may say that.
14. I would still be skeptical, but not yet challenged.
15. The statement seems personal, but de facto the questioning policeman could be exchanged for a colleague at any time. Before making false promises, it is better not to make any at all, or to keep it more general “We (as organization/police) are here for you.
16. This is very dependent on the atmosphere and situation. Depending on how sympathetic the official is to me, his statement can be oppressive as well as encouraging.

7.26.2 Group “Not Abused”

1. to personal statement
2. Too intimate for police officers
3. I also feel that this is too personal.
4. too personal
5. Perhaps this statement is also exaggerated, because I would be unsure whether this is really true, or whether it is just an empty phrase to get me to talk. That's something a person should rather say who you have known for a long time.
6. I think that depends on HOW it is said. It can be encouraging, but also exaggerated.
7. Also a promise that a stranger may not be able to keep.
8. There one slips immediately into the role of the victim, too close - does not fit the situation
9. It could at least follow “if you want it” otherwise- is he a psychologist or a policeman!
10. Can help with excitement/strong feelings as consolation, but only then helpful.

11. The statement tells me that something bad would have happened to me or the policeman assumes that something bad is happening to me. Nevertheless, it also conveys personal closeness and interpersonal support, both of which increase the feeling of well-being and the willingness to talk.

7.27 Expression of care (EC02): “I am interested in you/you are important to me.”

7.27.1 Group “Abused”

1. “You matter to me” is too personal
2. This is a foreign person, this statement is rather less credible. Better: I am interested in what happened to you and would like to help you
3. suggests false proximity, I find super stupid. Would have the feeling that the official wants to manipulate me.
4. maybe a bit unusual/too personal when it comes from a complete stranger police officer
5. Sounds like a hollow phrase... Too much of an unbelievable phrase.
6. Only if it is authentic ... and several meetings take place so that a relationship can develop. “Your well-being is important to me, and it is important to me that you feel better after our conversation(s)”.
7. sounds too formal
8. I don't know the policeman. EXTREMELY unprofessional
9. Why? No explanation, that makes me rather suspicious
10. I find the second formulation better/more appropriate
11. Skepticism, am I or my statement important ?
12. “They are important to me” sounds rather stuffy and I feel more like an object. “I am interested in you” helps me more.
13. Skepticism. It is about the investigation of a crime and not about the interest in the person!
14. This statement makes a queasy feeling
15. I would not take this statement seriously. This is too sweeping a statement, which has no personal reference to me or I will not be addressed personally.
16. Meaning does not fit into the sentence. You are important to me, I care about your well-being.... - there are 100 other formulations that better address the relationship level.
17. this is a bit exaggerated
18. The police officer does not do that, he just wants information from me!
19. second variant could quickly appear implausible or simply too close too quickly
20. I would not believe that.
21. That is a lie. The civil servant is not my therapist to whom I can turn for the next years in emotional crises and emerging traumas! Do not make false promises!
22. The formulation is too personal for me.
23. The official wants to help and support me. He wants me to be well.

7.27.2 Group “Not Abused”

1. this formulation would rather lead to nervousness, because I would have the feeling that my statement (e.g. the teacher has taken me in his arms from time to time) would trigger equally serious conclusions / consequences without me wanting to.

2. a stranger who is said to be important to you would be rather unpleasant to me.
3. To personal expression, which is unpleasant for me
4. Do I find it unsuitable
5. This seems excessive and would be uncomfortable for me.
6. too much
7. Does not sound real
8. this sounds again like a personal consternation and one could burden the other person, I would find a “I am there for you and I can stand it” more reasonable
9. unsuitable
10. Police station or dating portal?
11. ...Sure. - I find this statement a dry phrase
12. Uh...creepy. This is getting too close and too personal for an interview.
13. unpleasant to be told that by a stranger
14. Excessive, extreme! At best he is interested in what I have experienced. Disgusting.
15. could also arrive funny

7.28 Emphasizing that you are someone to disclose to (SD01): “If something has happened to you, I am here to listen to you.”

7.28.1 Group “Abused”

1. Should something happen again now? Does he mean the past?
2. Gives you security
3. I do not know if I need a listener in this situation... I don't want to talk.
4. well
5. maybe don't believe the policeman that he just wants to listen to me, since his job is to quickly find out what happened
6. I do not hear any demand here.
7. “I'm here to listen to you” is enough without triggering all the potentially bad things that have ever happened to you.
8. Why does he immediately believe that something has happened to me?
9. Without the first half-sentence a statement that does me good and strengthens my will to open up.

7.28.2 Group “Not Abused”

1. better if it is pointed out that this is a non-binding offer
2. Instead of “if” better “if”?
3. “If” shows that I am not expected to confirm the allegations. Furthermore, empathy is conveyed very carefully.
4. inviting, if something would have happened to me - so rather inviting
5. “If...” - Exactly, nothing happened to my character. So I could also talk calmly about what I really experienced and perceived
6. see above, it is there if then to prevent it in the future. Listening is only there to give him the facts (in a sensitive way) that he needs.
7. Strangely psychological, he is personally there to listen (instead of acting, arresting, etc.).

8. Better would be “if” and maybe the use of subjunctive

7.29 Emphasizing that you are someone to disclose to (SD02): “You can trust me and tell me calmly if something happens.”

7.29.1 Group “Abused”

1. How can I trust a stranger?
2. I don't trust anybody anymore for good reason, and certainly not because the person says so
3. How can I trust him, I do not know him
4. Unfortunately, this formulation does not inspire much confidence.
5. One reason why the question poser can be trusted could help.
6. generates trust
7. The statement does not fit the setting. I have no confidence in a police interrogation.
8. This has nothing to do with trust but with the obligation of secrecy. Once again: do not treat your counterpart like a child!
9. Feeling of loss of power. I would like to decide for myself if I want to talk about it. Police officers almost expect that something must have happened and I feel I have to answer affirmatively to his assumption.

7.29.2 Group “Not Abused”

1. Trustworthiness is expressed and thus well-being is increased
2. creates pressure, someone wants to hear something and I should just trust
3. Personally, I would feel very comfortable with this question, because I would not feel that the policeman had the intention to get a very specific answer

7.30 Emphasizing that you are someone to disclose to (SD03): “[Your name], if something has happened to you and you want it to stop, you can tell me about it.”

7.30.1 Group “Abused”

1. “If you want it to stop” increases the pressure & contradicts my situations because it has already stopped
2. “that it stops” - it's already in the past? my negative feelings towards it will certainly not be much better for the police officer
3. “If you want it to stop” implies that if you don't want to talk about the issue, it is not urgent enough to solve the problem. Better in this case would be “You could be very supportive of the other victims with your statement, if they are willing to talk about it.
4. does not fit to this situation. After all, it is a matter of a long past abuse, i.e. there is no acute danger.
5. “if you want it to stop” might increase the pressure, because it sounds like a condition
6. It has already stopped
7. The blame is then put on me when I cannot do it
8. I have to take responsibility for the deed?!
9. good! an offer, I do not have to, I may decide

10. Not really fitting to the situation, since it is already about 10 years ago and has therefore already "stopped".
11. ambivalent feeling: on the one hand, a burden, on the other hand, hope for relief
12. I think it is good that nothing is demanded, but that I am given the opportunity.
13. It has already happened to me, so it cannot stop and the whole statement is useless.
14. The phrase "you want it to stop" does not fit the given situation. It only fits if something happens to me regularly at the moment. If something has often happened to me but hasn't happened for 10-15 years or more, the phrase "you want it to stop" is inappropriate.
15. okay
16. gives hope, but could also build up pressure (in the sense of, it's your own fault if you don't tell it, besides, in this case the experience is in the past anyway) --> it is generally difficult or even impossible to put oneself in someone's place who has experienced sexual abuse, if one has not experienced it oneself, especially since every person reacts differently and accordingly finds other sentences pleasant/unpleasant
17. Attribution of responsibility if I do not talk.
18. Here a list would be meaningful with which assistance and support victims can count!
19. Police officers leave it up to me to decide whether and to what extent I report about my experiences.

7.30.2 Group "Not Abused"

1. typical sentence in a film
2. I get the feeling that the policeman thinks I don't dare to tell him that I was abused. That's why I'd like to put that right. That's why my need to talk is increased.
3. Do not understand this statement grammatically.
4. If something "happened", it has already stopped.
5. What should stop? If the suspected abuse was not many years ago, it no longer exists. And just talking to him helps, doesn't really make the pain stop if something had happened.
6. much too suggestive, nothing happened to me
7. Lets space to express fears - relieves
8. Personally, the question would put me under some pressure, because "being able" to read it feels like "having to".
9. 1. I CAN tell- good. 2. if nothing happened - presumptuous insinuation, extreme pressure to expect - if I experienced something very inviting, the promise that he will do something about it.
10. it implies an incident
11. Somewhat suggestive

7.31 Emphasizing that you are someone to disclose to (SD05): "I talk to many people and they tell me about things that have happened to them."

7.31.1 Group "Abused"

1. Knowledge about conversation partners is helpful to open me
2. does not work at all

3. The statement can increase the pressure to tell congruent stories with others or make you feel devalued because you assume that others have experienced much worse, especially if the event happened a while ago.
4. The own problem might feel “not important enough” for me, because the policeman is surely talking to other people about much more serious incidents.
5. That does not make my suffering any better, generalized statement...
6. suggestive
7. And what do I get out of it personally?
8. Nevertheless, he cannot conclude that I am
9. it's good to hear that others have gone through it too, but nobody has gone through exactly what I have gone through, that's arrogant to call it that
10. I have the feeling that the interviewer has experience and knows what he is doing
11. How do other people know what happened to me?
12. Takes away the fear of stigmatization/ condemning remarks.
13. This is not well formulated and with me he/she would not have access to it.
14. lies in the nature of things
15. This does not relieve me, because it does not matter to me that she has already heard a lot. It may reduce shame.
16. Because you are such a nice person or what? Stay professional and refer to your many years of professional experience if you have something to say on the subject.
17. Just because others have done that, I don't have to do it... What do other people have to do with my experiences now?

7.31.2 Group “Not Abused”

1. gives a positive sense of community
2. Nothing has happened to me (!)
3. He should not talk too much about other people
4. That sounds so stilted in my ears, I cannot judge it.
5. that “what happened to them” is misunderstandable, one might think it is about me.
6. ... and then I have to do that too? Creates pressure
7. Yes, but do I have to do that too?
8. How nice for the person concerned - and what does this have to do with me? (only provokes sarcasm)
9. So what?

7.32 Emphasizing that you are someone to disclose to (SD08): “[Your name], my job is to listen to people who have been hurt.”

7.32.1 Group “Abused”

1. Mentioning the job aspect does not give the impression of strong empathy
2. That sounds more like he's more likely to do the interview because he's paid to do it.
3. the statement: ...something has happened to them implies that something has happened to me

4. sounds like from a bad crime scene. Besides, the officer does not know yet whether something happened to me --> suggestive
5. Takes the personal out of the conversation. ....is just the job he does.
6. I can assess it better, transparency
7. I cannot do anything with it.
8. “my job”
9. This “This is my job” track seems very cold and I feel like a number on a paper, like a case that should be closed as soon as possible. It also portrays me as a helpless victim, which I may not want to be.
10. What bothers me is “my job”... as if I am only a “number” that has to be processed in this case.
11. factual
12. so one-sided
13. I do not play a role.
14. It is “only” a job for him. He is not really interested in me and my well-being. He can't put himself in my place and he doesn't care about me.
15. Implies that I was only loaded because it is assumed that something happened to me.

7.32.2 Group “Not Abused”

1. Implies that I am talking to him because something has happened to me that is not true.
2. either one feels well taken care of or with emphasis on the job human component (warmth) may be lost
3. but nothing happened to me! somehow that puts pressure on me
4. This information is not important for me - but it can be for others
5. this is about me, besides, nothing has happened to me
6. Nothing has happened to my role
7. It assumes that something has happened to me. Very suggestive.
8. That “sucks” too. It sounds zero empathic but distanced, rather sensational. I would not like to tell such a person something personal, let alone something intimate.
9. Does it seem somewhat suggestive/intimidating for the official to assume that there has been abuse (which may have happened to me), and as if he was only there for such statements
10. I like the word listening. The word happened again presupposes a negative experience that I have never experienced.

7.33 Emphasizing that you are someone to disclose to (SD10): “[Your name], I really want to know when something has happened to people. That's why I'm here.”

7.33.1 Group “Abused”

1. too intrusive, especially through the word really
2. Unprofessionally formulated
3. Term encountered ?
4. “People” has a very generalizing effect and I don't really feel addressed personally (despite being mentioned by name)
5. for me: too demanding

6. okay
7. Civil servants show interest without creating pressure of expectation. Formulation provides for a pleasant discussion atmosphere.

7.33.2 Group “Not Abused”

1. Pressure of expectation slightly increased, as indirect reference is again made to possible rape.
2. sounds like personal sympathy (which can be incriminating) and is suggestive (if (instead of if) something happened to me...)
3. Sounds not quite credible, what is really their job
4. even more blatant than question 20. feel as if I should absolutely denounce the teacher and best of all drag many of my former classmates into the public eye as abused
5. Because nothing happened to me in my role, I would feel that the policeman would try to force me to mention something that never happened
6. He is not there to find out, but primarily to ensure that perpetrators cannot continue unchecked. Good will yes, but he does not seem to have understood this fact. If I was good enough, I would point this out to him and if he could accept it would be a good basis for further action, if not, I would not talk to this person any further.
7. if it's authentic, fine. this very personal touch seems personal but inauthentic to me.
8. In itself a nice, emphatic statement, but it strongly conveys the expectation that something must have happened. Perhaps the word “if” would be better in the subordinate clause.

7.34 Emphasizing that you are someone to disclose to (SD11): “[Your name], here people can talk about good and bad things they have experienced.”

7.34.1 Group “Abused”

1. Doesn't sound particularly ambitious, but rather recited.
2. but I do not want to speak
3. skepticism, is after all police
4. When I come up with bad things, I feel “ungrateful” that I have nothing good to say.
5. I am an adult
6. For me this is a “zero eight fifteen” saying and I would switch to “Durchzug”.
7. The “here” confuses, what does the place have to do with it?
8. too general
9. What I miss in the question is the remark that this is a protected space where I can tell things. I would have liked the police to have psychologists/psychotherapists in the background who could catch something when I tell something like this for the first time.
10. But I am aware that there are consequences. To me this is too general and judgmental.
11. Should you tell about your last visit to the cinema or what? We are not here to make friends, but to record a statement or? So limit yourself to the factual, professional part.
12. Little interest, because what should be “good” about the experience?

7.34.2 Group “Not Abused”

1. I find this name very unfavorably selected.
2. He sits with me in a confidential conversation, perhaps even at my home, and says “people can come here” ?! Does not sound real.
3. you are not invited to the police to talk about good things
4. Please without valuation and how is it with itself load?
5. like question 19, but the situation is much more confining and suggesting that something has happened to me
6. Since I have experienced nothing in my role, I would feel comfortable here, but if I had experienced something, my willingness to speak would probably decrease
7. I see. And?
8. sounds too open, rather for therapy setting

7.35 Emphasizing that you are someone to disclose to (SD12): “My work is to try to help people.”

7.35.1 Group “Abused”

1. I find the statement seems very artificial and inauthentic. I also don't think that this is the job of a policeman and would probably find it ridiculous if he told me something like that.
2. Better formulated than question no. 45
3. I don't believe in helping as a police officer. That's not his job either, he has to investigate and my needs are ultimately not relevant
4. but I'm not helped here, they want something from me, so he should call it that
5. I find “my work” better than “my job”, and I also find the emphasis on helping rather than “people who have been hurt” better. Nevertheless, it doesn't seem very personal, but more like a duty for the person and I almost feel like a burden.
6. Trying is good, but doing is better. The sentence or the statement does not help me.
7. without the “try” it might be better. It would be nice if someone could do it and not just try.
8. okay
9. Not believable.
10. just “trying”?

7.35.2 Group “Not Abused”

1. I like it better than statement 9.
2. More neutral formulation
3. I don't know what he wants to say with this. Is too vague
4. Surely there sits rather an investigator, no helper
5. Just “try”? The work of the police IS to help people and NOT to just “try”.
6. unclear whether I will be helped in the situation
7. Hm. So what? Unnecessary. At least it was a nice try...
8. Empty phrase (for me personally now) that is not necessary. Especially nothing like this in front of a conversation. And “try” sounds weak. Maybe okay in the middle.

9. Here again a wrong understanding of roles is conveyed. Doesn't sound authentic, because the policeman is not there to help people.
10. Unfortunately, the police often help the victims less than they help prosecute crimes.

7.36 Generalization of the child's expressed difficulties (GD01): “Many people find it difficult to talk about it.”

7.36.1 Group “Abused”

1. Suggested that many people have been able to talk about something similar. But maybe I can't do that in this situation and then feel less worthy.
2. This sounds like the official would reduce the burden of my situation
3. I am not alone, who knows that others feel the same way and understands that, that is good
4. I do not feel pushed and I do not feel “abnormal”.
5. It is good to know that you are not alone in this
6. This is too sweeping and does not give me access to the interviewer.
7. true
8. I am understood in it when it is not easy.
9. I always thought most victims peddle their experiences all day long. It is not easy for a normal person to relive his or her trauma mentally.
10. I am not the only one who feels this way! So it is “normal”

7.36.2 Group “Not Abused”

1. Shows that you are not alone, that many people are like that and takes the pressure of expectation away.
2. this is true and I think it is good that no attributions are made to me here
3. Relieved, could feel understood
4. I personally would feel more comfortable through the apparent empathy, even if nothing happened
5. Again, I find it difficult to put myself in this situation, as I cannot confirm the accusations. I would feel pushed in one direction.
6. Maybe that also means that I don't have to, even if something was, at least not at this moment, that relieves me.

7.37 Generalization of the child's expressed difficulties (GD02): “Many people have secrets and I am here to listen.”

7.37.1 Group “Abused”

1. instead of using the word secrets use expressions like: many people have experienced things that are difficult to talk about
2. Why secret? It sounds like I'm being blamed for not talking about it earlier
3. the feeling of being understood, but still suspicious
4. builds up pressure; good/bad secrets, guilt for it?
5. I have the feeling that I am not “abnormal” and I feel more comfortable.

6. Why does he think that I am hiding something?
7. Put me under pressure that I have to tell something
8. Secrets are negative in general and so I am evaluated negatively
9. Depending on the course of the conversation, this question might encourage you to talk. Taken out of context, it seems strange.
10. The question is too general, not very sensitive.
11. unfortunately I can't tilt back, I'm not sure anymore if I always paid attention to the square brackets
12. A police officer does not just listen.
13. We all have them, don't treat me like an idiot.
14. “Secret” as a rather positively connotated term is very inappropriate in this context. Why do I have to tell the official my personal secrets now. This is unpleasant for me.

7.37.2 Group “Not Abused”

1. Would rather arouse my suspicion.
2. I have no secrets to tell you about.
3. Sounds unprofessional
4. strange formulation
5. I don't really want to reveal my secrets.
6. meaningless statement
7. ...and now at confession?
8. Again, I find it difficult to imagine this statement, as I do not feel affected and cannot confirm the accusations.
9. I feel as if the interviewer is implying to me that there is something I do not want to say. I do not find “secrets” a very appropriate choice of words. This sounds like “forbidden” and “guilt
10. see above already again so similar, analogous the same remarks.
11. Strange formulation with the secrets and listening to them.
12. The statement sounds slightly reproachful.

7.38 Empathy (EM01): “I understand that the interview is quite long.”

7.38.1 Group “Abused”

1. If I say nothing more now, I may leave immediately
2. Understanding me increases my well-being.
3. Remark is appropriate, feel taken seriously
4. I think it is good that the effort is acknowledged
5. That makes me feel more like he's complaining that it's taking too long.
6. Very strange question. I would have expected something like that from the interviewee. or formulated differently as an announcement of the interviewer.
7. ??? Offer breaks if necessary!
8. unsuitable
9. What is there to understand?
10. He knows that it was not easy for me to talk about such things.

7.38.2 Group “Not Abused”

1. The length would not bother me. This topic deserves time, I think.
2. yes, and? that this can be exhausting for me? There is a part of the validation missing. Or does he mean that it is exhausting for him?
3. Seems a bit like an excuse that I'm being asked to do so much - appreciative
4. Uh? He's doing it, all weird.
5. in case of complaints about the length, okay, more a hint about the importance of details or so but maybe better

7.39 Empathy (EM03): “I understand that you find it difficult to talk about it.”

7.39.1 Group “Abused”

1. door opener
2. I thought the officer didn't even know that something had happened to us. In view of this, the statement seems very suggestive.
3. Understanding helps with my well-being, but not with my fear of speaking
4. I don't feel like a problem (most of the time others don't understand if you don't like to talk and push about such things)
5. I would feel understood
6. It is a means of the questioner to inspire confidence, even if he does not really know my feelings.
7. in the questions, in my opinion, “I understand” occurs too often.

7.39.2 Group “Not Abused”

1. Motivates me to keep talking.
2. Although I have no difficulty in communicating the information, it is formulated in such a way that my willingness to speak and my sense of well being decreases. At the same time, this increases the pressure of expectation, since it implies that I have experienced something I do not want to talk about.
3. Understanding / Putting yourself in the position of the interviewee gives a good feeling
4. Showing understanding is good
5. Why does he understand that? How does he get it? More pleasant would be: “I can imagine that....”
6. Understanding cannot be right in this context, because no one really understands the feelings of others.

7.40 Checking the child's feelings (CF01): “How are you doing so far?”

7.40.1 Group “Abused”

1. Purely a phrase of courtesy. I would rather get the conversation over with quickly than get bogged down with such questions.

2. Appears very flippant and not appropriate to the situation. I would instinctively just say “good” or “quite okay”.
3. Stupid question
4. Gives me the feeling that my feelings are taken into consideration
5. Inappropriate question in the situation
6. stupid question
7. I find the question unnecessary - I probably don't feel well or maybe I am relieved after the interview.
8. The question is harmless for the time being and would also be more generally understood.
9. Very good, gives you the feeling of being able to take a break or stop in an emergency with a clear conscience. Sometimes it needs this reassurance
10. The official is really interested in me and inquires whether we can continue the questioning within this framework without hesitation.

7.40.2 Group “Not Abused”

1. general phrase, to which one usually answers with good anyway
2. Friendly, neutral question with which I have no problem.
3. can sound like a phrase, so that one assumes that the opposite party does not want to hear an honest, longer answer at all or that it increases the well-being, because interest in the own person
4. What is this now - would spontaneously come to my mind
5. good valve
6. So far? That would seem too brisk to me personally and not as if there was any real interest
7. Strange question for a police officer, rather for psychologists (it sounds too small talk for the very organized police talks, too irrelevant)

7.41 Checking the child's feelings (CF02): “We are now finished with the interview. How do you feel now?”

7.41.1 Group „Abused”

1. Considering the end of a difficult questioning for me, I feel comfortable and no longer have the feeling that my answer to this question will be analyzed in detail, as probably the answers to the questions asked before.
2. The question implies a little bit that one should feel relaxed and relieved now. Better for me would be “What helped you and what was difficult for you?
3. I find this question very good in the idea of having just talked about something like this
4. I hope you feel better now that you have had a chance to talk about it. We are very grateful to you, because statements like yours are very important for us to convict perpetrators. ... How do you feel now? (The “How do you feel now?” comes too quickly after the “We are now finished with the interview. You think you are finished and then you are asked again.
5. I don't want to talk to a police officer about this any further. Feels like pressure
6. It's good that the interviewer doesn't directly break the tents and disappear, but that you are still “supported”.
7. Why “interview”?

8. I would be happy to be given the opportunity to talk about my negative feelings
9. The statement “we are finished now...” is too abrupt and as a conclusion not very elegantly solved. That would bother me.
10. The question can be asked by a therapist, not an investigating policeman !
11. 2x now? “We are now finished with the questions, would you like to add anything else? ... How do you feel at the moment?”. Provide contact details of psychologists in case the person is not ready to go home so agitated or needs support in the future.
12. The term “interview” seems less oppressive and threatening than the criminal procedural measure of “interrogation”. It is done and yet the official is still interested in me, although he now already has my statement.
13. Creates pressure that I may have to feel “bad”.

7.41.2 Group “Not Abused”

1. a good situation to possibly come to the point (if not already done) that you feel a little uncomfortable, because you don't know what is true with regard to the teacher and you are now unsure how to express yourself/ how to present and reproduce things as well and meaningfully as possible
2. Concern for well-being is very friendly.
3. I like these questions after the conversation is actually over. That is cautious.
4. It's nice to know that I am also interested in how I am doing now. Caring, not only demanding
5. Very empathetic
6. A little stiff.

7.42 Checking the child's feelings (CF03): “How did you feel before we spoke?”

7.42.1 Group “Abused”

1. What does that matter?
2. Is not relevant for me in this context and is not his business
3. Shows interest of the interviewer beyond the actual question I think that's good.
4. Gives space to talk about my feelings/fearings without demanding anything from me
5. This question is not relevant.
6. I think it is a police interrogation ? The description of my feelings is out of place
7. Whether I could continue speaking would depend on whether I would like to reveal further.
8. Probably better than now but still nervous and excited, worried, unwell,... can be summarized as “not particularly great”.

7.42.2 Group „Not Abused”

1. Neutral question
2. I notice that well-being and willingness to talk are very much linked, that is, with me.
3. Depends on the time of the conversation and whether I want to discuss my feelings with this person
4. I feel in good hands and my worries and fears about the conversation are taken seriously

5. I personally would be pleased about this request
6. I am in a police interview, not in a therapy interview. I find this question leads too far...
7. Irritating question. Rather evokes defense with me.
8. Relevance?

7.43 Checking the child's feelings (CF04): “How did you feel when you spoke to me today?”

7.43.1 Group “Abused”

1. Shows interest in me & my situations, less focus on pressure to educate.
2. Possibly too much, depending on the emotional state.
3. What is the question?
4. my feelings are taken seriously
5. Encourages reflection on oneself.
6. rhetorical question
7. It looks like I've got it behind me.
8. Ask about your current state of health.
9. Explore to what extent the confrontation with what I experienced then still burdens me today and how my body reacts to it.
10. Is this relevant? Do I not want to hurt the official if I say I feel uncomfortable?

7.43.2 Group “Not Abused”

1. I would feel a little uncomfortable because I feel that the only socially acceptable answer is “good”.
2. I find inquiries after the interview so attentive and cautious.
3. Feedback as control parameter
4. I don't want to reveal that much now.

7.44 Exploring emotions (EE01): “Tell me more about [your fears; your anger].”

7.44.1 Group „Abused”

1. I am allowed to talk about my feelings instead of what happened, it is easier
2. I can imagine that it is difficult to talk to a police officer about his feelings.
3. Concrete questions about the fears and anger would help me more.
4. ... if the question does not come too early, ok.
5. This is none of his business
6. How does he know that I have fears anger?
7. If this does not come right at the beginning of the conversation, it is fine.
8. Usually I am more afraid to talk about my fears. Especially in an unfamiliar environment.
9. asks such an investigator ??
10. If I don't want to deal with experiences, then I don't want to deal with my feelings about them either.
11. He knows how I feel right now.

12. maybe I don't have anger and could feel the pressure of this statement that it is normal to feel anger.

7.44.2 Group “Not Abused”

1. Formulated as a question even more inviting
2. Here I would personally feel that I could say that I was afraid that the police would only be interested in a certain statement and not in my statements
3. In this situation, I do not want to (have to) deal with my fears, but rather report on events ....
4. 1. does that do something to the matter????? 2. am I ready for it right now?
5. With the optional demand: how did it feel, what did you think, what did you do, etc. quite well.
6. It is suggested that I should feel fear/rage. However, if I have never been abused, I may not feel any negative emotions. This should also be considered.

7.45 Exploring emotions (EE05): “I see you are crying [or other emotional reaction]. What happened?”

7.45.1 Group “Abused”

1. Better: If you feel like it, you can tell me what happened
2. could perhaps be perceived as somewhat unpleasant if crying is explicitly mentioned
3. When I start to cry, it is obvious that something must have happened. Then I personally prefer to take some time to collect myself
4. Pressure of expectations/ curiosity(?)
5. It depends on whether I am crying because the question poser formulated something too insensitive or whether I was simply overwhelmed by my memory.
6. Stupid question
7. that I see them crying could be left out, or why mention the obvious again?
8. It would be uncomfortable for me to have to talk at such an emotionally charged moment. I would first want to calm down a bit
9. “What happened?” - this question would be too clumsy for me and I would also not feel taken seriously.
10. May I hand you a handkerchief.
11. When I cry in front of someone and then especially when he or she also talks to me about it, my well-being decreases.
12. trying to learn more
13. maybe better: “Would you like to talk about it?” (no one is forcing you)
14. To comment on the obvious does not help, the witnesses are not stupid after all. “You want to tell me what happened?” would be better here.
15. Reflection of my emotional world. The official is interested in my well-being by means of an openly asked question.

7.45.2 Group “Not Abused”

1. Very direct expression and invasion of privacy

2. A civil servant or an official, oh no, the description says yes, it is a male policeman! So: a civil servant who could handle tears with such sovereignty would - if he is authentic and it doesn't sound like a hollow phrase - have immediately gained my trust.
3. unpleasant situation
4. “what happened” is suggestive, one could ask: “would you like to tell me why?”
5. I would like to cry without explanation, but you may hand me a handkerchief and wait until I like to talk again
6. clumsy intrusion into my protection zone
7. It's nice that the policeman can see that, but he didn't have to mention that now. I can feel myself crying. How about offering me a handkerchief?
8. It is difficult for me to imagine crying when I assume that this is an accusation against the teacher that I cannot confirm.
9. I am crying ....obviously I am not feeling well right now. I would like to have an empathetic counterpart who can stand my crying and gives me time to collect myself.
10. What happened leads to pressure of expectation rather than “What's on your mind?
11. It depends very much on whether a basis of trust is already there - it can be one way or another.
12. I find it hard to imagine that I would cry in this situation
13. is attentive
14. I would be uncomfortable to be spoken to about crying in this way.

7.46 Exploring emotions (EE09): “[Your name], what would happen if you told me about it?”

7.46.1 Group “Abused”

1. Since it's been a long time, it might not occur to me that something might happen. But when he suggests it, such ideas come to me, he is the professional
2. Fears can be expressed and addressed
3. Addresses my fears and anxieties, can tell them
4. This makes me feel harassed or rather offended (“why don't you tell me anything?!”), but very soon after that I realize that the person is right and I start telling.
5. Everything could come up again and I have to deal with the memories I buried.
6. The wording would confuse me a bit. Since I can't know what will happen, of course, I should rather be asked about my fears.
7. This question would not appeal to me.
8. If, after having experienced sexual violence, I developed any kind of behavior or disorder, such as an anxiety disorder or at least that I get too involved in things, a lot of bad things would come to my mind (which I consider bad, at least) that would rather prevent me from telling details.
9. ??
10. little well-being, because thoughts of what has happened
11. Questions always expect an answer, in this case you may not have a concrete answer because you are simply afraid
12. Exactly this question would scare me, because I wouldn't know what could happen.
13. Do not use the name inflationary.

14. Too indirect questioning; tends to cast doubt on whether a statement would be meaningful/productive.

7.46.2 Group “Not Abused”

1. The question takes some pressure off
2. So much sensitivity and alertness would be great!
3. nothing happened to me, I would be afraid that he would not believe me, but then I could say that
4. of what? Is there nothing spectacular to tell, or are you implying something?
5. very strongly manipulating and putting under pressure
6. Here I personally would have the feeling of being pushed to a certain statement, or being persuaded to make an incorrect statement, by feeling that my primary school teacher would be better off in prison
7. Of what? I assume that nothing has happened to me.
8. Triggers new thought spirals instead of concentrating on concrete things in the past
9. Why does he want to know that? Could he protect me from possible consequences? Or guarantee me that they will not occur? Otherwise: what is it to him?

7.47 Exploring emotions (EE02): “[Your name], is there something that worries you?”

7.47.1 Group “Abused”

1. I find it okay, open, not too pushy.
2. I do not know if I can really trust
3. A more open formulation would be better (purely socially I would have the feeling to answer with “no”)
4. The open formulation gives you the opportunity to decide for yourself whether you want to report something
5. The question entices people to confide in each other.
6. I would probably not tell a stranger, especially since it was noted in the case description that I wanted to suppress it
7. Probably already but how I pay my rent or something similar is not the question now.

7.47.2 Group “Not Abused”

1. A more concrete question would be desirable
2. Asking for concerns in very general terms and therefore with a positive effect on well-being and willingness to speak. Pressure of expectation is also taken away.
3. I do not like to talk about my worries.
4. much too general, this leaves you in the dark about what the person opposite wants to hear
5. It is of interest how I am doing, fears, anxieties get a place - if I want
6. Personally, I would find the question less researchful without mentioning my name
7. I find openness to results good
8. unclear, whether general or retrospective to the primary school period

7.48 Echoing emotions (CE01): “You said you were sad/raged/that you cried.”

7.48.1 Group “Abused”

1. I would find it easier to answer a question. I might find it difficult to answer a statement I have made
2. Offers a good opportunity if I want to go into detail and shows interest and that the other person is listening.
3. I register that the person is listening to me correctly
4. With regard to which situation should I have said that?
5. gives the feeling that you are being listened to attentively
6. Too much demand
7. feel understood
8. An unpleasant topic, but this sentence helps to address it
9. Shows me that I was listened to
10. Frager has recognized my situation. A psychologist would ask this question more than a policeman...
11. one is perceived and taken seriously
12. What kind of answer do you want? “Yes, I said so”?
13. Reflection of my emotional world. The official can put himself in my position.

7.48.2 Group “Not Abused”

1. in what context?
2. here the context would be relevant, because I don't know a real reason why this statement should fall.
3. Why should I say that when I have positive memories of the teacher?
4. It is easier to react to open questions than to statements
5. that is validating
6. Inviting to tell more
7. goes too deep into or too close to the person too early
8. I cannot imagine being angry/crying when I cannot confirm the accusations.
9. Active listening is well received
10. It would depend very much on the situation in which this demand comes, and the “how”. It could tend to work in both directions: Increase well-being and willingness to talk and reduce the pressure of expectations - or vice versa.
11. active listening is good
12. If I said this during the interrogation, it shows that the official was attentive. This makes me feel comfortable and I would like to continue (hypothetically).

7.49 Open questioning about feelings and thoughts during the incident (OI01): “You said you were sad/disgusted/wanted to run away. Tell me more about that.”

7.49.1 Group “Abused”

1. When it comes to memories, my sense of well-being decreases and the pressure of expectation to go into even more detail increases.
2. (.....). What in the situation triggered this feeling?” A what question might be better than Tell me more about it.
3. Too much pressure of expectation
4. depends on the relationship
5. “Tell me more about it” is too much of a challenge for me. This takes away my decision whether I want to tell you more about it.
6. this is a factual question, it's okay.
7. possibly formulate the second one as a question, if you want to tell more about it
8. I would consider that greedy and probably say nothing more.
9. “Can you describe your feelings more precisely?” Ask more concrete questions, that gives more security.
10. Reflection of my emotional world. The official can put himself in my situation and shows interest in it.

7.49.2 Group “Not Abused”

1. I don't really know now why I am disgusted/ sad/... why I should be disgusted/ sad/... But in principle I would like to continue talking after such a request.
2. Unpleasant
3. great, because it is an open question that is not suggestive
4. more distance to the feelings, talking would perhaps make it easier
5. I feel impaled and maneuvered into a corner, almost threatened “talk away”, without any ifs and buts
6. I said I was sad, so telling more is not so easy. - Statement little empathic
7. Again, I can't imagine having said this, as I cannot confirm the accusations.
8. Sentence in imperative is very demanding
9. Again, it depends extremely on whether or not I feel well looked after there before, or not. Depending on the situation, this could be a friendly invitation or a very unpleasant pressure of expectation.
10. unpleasant situation

7.50 Accepting and recognizing emotions (AR02): “I understand what you are trying to tell me.”

7.50.1 Group “Abused”

1. In this situation, this statement might seem like a cliche. One cannot assume that the person sitting opposite really understands what I am saying, since he or she may not have been in a similar situation before.

2. Depends on the context.
3. The evaluation is only true if the person actually feels that the police officer understands him or her and does not just say so.
4. It is too interpretative; one has the feeling that he is not really listening and that his image has already been formed.
5. “... trying to say”, comes across as if the interviewee is not expressing herself clearly. Better would be: “I understand.”
6. I do not do it well enough
7. Nice. Then perhaps paraphrase briefly or repeat what was said to check if you REALLY understand what was said.
8. I doubt it
9. my thought: male police officer (probably) without abuse experience - can he understand me?
10. I find this question a bit presumptuous! Very suggestive!
11. This phrase should be handled with care. Who understands the horror that an abuse victim has experienced? “I probably can't even begin to imagine what you've been through but I'm trying to understand it” or “...but we're trying to find out the truth to be able to draw conclusions if necessary.
12. He can understand my experiences.
13. Depends on the context... if I really can't find words to describe things: good // if not: can he see the future?

7.50.2 Group “Not Abused”

1. How does he know what I am trying to tell him? That should be more concrete I think
2. I find the agreement and signaling of empathy important.
3. Feedback is good.
4. Pretends too much understanding
5. he does not know whether he understands what I want to say, I can only “believe” that he understands
6. Telepathy?
7. Do you?
8. Feel rather skepticism and ask me if he/she really understands this. Would rather wish me to mirror my statement by paraphrasing.
9. Suggested that I do not manage to say clearly what I have experienced
10. was not exactly this question before? Oh no, it was in the past. I have understood. Not better that way either. How would he know that if I obviously haven't said it yet? Awkward.
11. Understanding and “trying to say” sounds contradictory

7.51 Accepting and recognizing emotions (AR03): “I understand/can understand what you say.”

7.51.1 Group “Abused”

1. Understanding and comprehension can be out of place here.
2. the person cannot understand it!

3. In principle nicely meant, but questionable to what extent a man can understand such a thing.
4. I go with the feeling that I am believed
5. I would feel understood
6. If the person is not a victim himself/herself, he/she cannot understand this. The sentence is fatal.
7. If something like “I understand you” comes up too often in a conversation, it might sound like a cliche and at some point you don't feel understood anymore, but get the feeling that the person opposite is just saying that, but actually has no idea. This can lead to frustration or disappointment.
8. Can he really understand this ?
9. Probably he cannot understand what I am feeling. Unless he has had similar experiences or is very empathic.
10. Only if it is de facto so, do not use it as an empty phrase.
11. The official is interested in me and can put himself in my shoes.
12. follow

7.51.2 Group “Not Abused”

1. I find “understanding” difficult in this situation.
2. That usually feels good when someone says something like that. At least I do.
3. he cannot understand
4. I am understood, does good
5. Personally, I find this objection encouraging to continue talking, that nothing happened to me and how I perceived everything
6. Even more pleasant with: “I think, ...” and then ask again whether it is so ... true.

7.52 Reassurance (RA01): “Do not worry. I won't tell/address any of the concerns that have been mentioned above].” / “In some cases, it is possible to help [families with problems/people who have hurt others].”

7.52.1 Group “Abused”

1. It seems a bit like a phrase the officer says to get me to talk.
2. Concerning the well-being: I assume that this statement would increase the well-being in this situation, because the person apparently often thought about the fact that other people should not know about it (since they never talked about it with anyone), but if the person should assume from the outset that the contents of the conversation will not be passed on anyway because of the obligation of secrecy, this explicit assurance could perhaps have the opposite effect, because not telling others about it does not seem so obvious anymore.
3. One is with the police and it is about solving the crimes. Sounds implausible to say that no one is told
4. Stupidly formulated. Better: You don't have to worry about me passing on information to others. (or so)
5. could be formulated even more convincingly, e.g. go into more detail about who has no access to this information

6. Statement basically conveys security, formulation again very unprofessional. Rather: “what you tell us here will be treated confidentially”.
7. Do not feel taken seriously because I am worried. Besides, I know that it will be passed on.
8. why does he emphasize that? that's a matter of course! and whether I'm worried or not is up to me
9. Police? Information will be forwarded.
10. Gives feeling of “safe space”, I feel liberated.
11. Creates a protected framework that greatly reduces the fear of negative consequences
12. This is not well formulated and does not calm down a bit.
13. that's what I expect: Secrecy
14. I thought it was a cop? He will tell others about it! Maybe describe more precisely which others he means.
15. That sounds like a lie. After all, the information is important for the investigation.
16. This is not believable.
17. We are not in kindergarten. Please refer to your legal duty of confidentiality and ensure that you also comply with it, with the appropriate legal exceptions, if your counterpart represents a danger to himself or others.
18. Easier said than done, not to worry. Besides, my statement will be recorded. What happens to it?

7.52.2 Group “Not Abused”

1. That is a downright lie
2. I would expect this, especially if the person leading the conversation is a psychologist.
3. Implies that there is something to hide. At the same time, however, considerate behavior that induces a feeling of security.
4. He should say, I “may” not say anything, “there is confidentiality”
5. What others?
6. not confidence-inspiring
7. That cannot be and therefore fuels my mistrust.
8. Confidentiality is good, it opens a window of trust, I would find an explanation here, who could get which information when, but it is still good
9. and I'm supposed to believe that?? Please be a little more precise!
10. Because I have experienced nothing negative in the role, the statement would put me under pressure
11. What else am I being questioned about? I cannot believe this statement. After all, the person is investigating.
12. suggests that there might be something to tell
13. That sounds somehow unprofessional and childish
14. At the beginning of the conversation I would have liked to have had an agreement on confidentiality and not a succinctly thrown in “Don't worry”. That does not feel serious.
15. Data protection situation is more important
16. If this has not been made clear BEFORE the conversation begins, then hops and malt are lost anyway. and the word CARE is extremely inappropriate in this context.
17. In police files it is clear for several to see... even when it goes to court

7.53 Reassurance (RA02): “Sometimes it helps people if they can talk about something and do not have to keep a secret.”

7.53.1 Group “Abused”

1. avoid the word secret
2. I would not call it a secret. More like a traumatic experience.
3. this is true, but the police is not necessarily such a place where I confide in myself for the first time
4. That makes pressure, but “good pressure”. I like that.
5. I am an adult today, please talk to me like that
6. “no secret” could make me feel that I could not have a say in which details I tell and which I don't. This could perhaps lead to the feeling of telling everything or nothing
7. “Secret” is negative in general and therefore one feels negatively affected/evaluated by this statement.
8. already better
9. Supporting factors are well pointed out for me: no more carrying around the burden of secrecy! Would rather encourage me to talk!
10. Works like a phrase.
11. as above. The term “secret” is clearly too positive in this context.
12. Difficult question, but feels redemptive.

7.53.2 Group “Not Abused”

1. has a very insinuating effect on me and at the same time as if I would not find a hearing with my rather positive description of the teacher and only the negative aspects would be taken up
2. I have no secrets, therefore pressure of expectation slightly increased.
3. With this he would hit the mark with me and I would relax very much.
4. Well formulated: Option given to talk about it without building up additional pressure.
5. This is suggestive, nothing happened to me
6. very understanding and general
7. Assumption. Who thinks I would make a secret of it ....
8. Works a bit like a bait.
9. See note question 8.
10. This implies that I have a secret. Depends on the situation ....when I have already made it clear that I cannot remember and then hear this statement, then I think that my counterpart does not believe me.
11. It helps as far as I know even most of the time, and at the same time this question conveys that he REALLY does not want to put pressure on you. Finally a human being again.
12. Wrong understanding of the role of the police officer. He is not a therapist. And he implies that you have a secret.

7.54 Removing responsibility from the child (RE01): “If [something happened; someone hurt you], [it is not your fault, because you are not responsible for it].”

7.54.1 Group “Abused”

1. Understanding is signaled
2. wonderful, good to hear that
3. Takes away my feelings of guilt, which is very important
4. Takes away the fear of being convicted/accused of something. “If” leaves all options open
5. very sensitive
6. In order to be able to say something, it is easier if the blame is taken away from me. Very good approach.
7. This statement would only help me if I had expressed that I felt responsible. Otherwise it is too meaningless for me.
8. Relieving feeling. I really couldn't help it that the assaults happened...
9. The statement in the right context would possibly also increase my well-being.

7.54.2 Group “Not Abused”

1. also here a pleasant formulation, since it does not give the impression that the questioner has a ready-made opinion
2. I find it important, especially with children, to repeat this often.
3. Also no direct implication that something has happened to me and additionally avoidance of feelings of guilt
4. a victim who reproaches himself will think that this is nice, but on the emotional level it will not get through and it is a statement that may not be true at all. I would not say anything about that. I would only ask questions about whether the person is blaming himself...
5. Could have a relieving effect
6. Although the pressure of expectations is increasing, so is the willingness to speak in order to correct any false assumptions.
7. Women must first learn..... to believe this - at least it is reassuring that HE knows that!
8. Could perhaps reassure in the right place, but find the assumption that something happened and the reaction directly to deliver something difficult (perhaps suggestive)

7.55 Removing responsibility from the child (RE02): Generalization: “If something happens to children, it is not their fault.”

7.55.1 Group “Abused”

1. prefer not to formulate as generalization
2. Unintelligible question for me
3. Maybe a bit too general.
4. I don't blame myself that something happened to me, why so strangely formulated?
5. I do not feel guilty and the police officer feels the same way
6. very good! reduces my sense of guilt, lifts it to a structural level, away from my person
7. Error ???

8. In itself good. But when I blame myself, this phrase seems rash and shallow.
9. explanation missing, too general
10. Takes away the fear of being judged for something
11. Contentwise completely ok, but I don't find it linguistically appealing solved and therefore it doesn't appeal to me.
12. I do not like this sentence. It somehow implies that if something happens to people who are not children, it is their fault. Of course that is not meant. But if something similar happened to me afterwards - as a teenager or adult - I might be inhibited to talk about these incidents. Because I could misinterpret the sentence and see it as confirmation that I, as a (nearly) adult person, am also to blame.
13. true
14. Such general statements are not so important at such a moment. It would not go down well with me at such a moment, because many victims think that they are guilty anyway because of possible perpetrator projects.
15. Instead of the keyword “generalization”, a short description of the situation would make it clearer when or in which context this statement is used.
16. It is not only like that with me, but with others as well.
17. Doesn't do me any good.
18. Since it is known that victims of abuse tend to blame themselves, such insurances should be standard.
19. What do other children have to do with it now? But this statement is not always true!

7.55.2 Group “Not Abused”

1. depending on this, it could look like the interviewer does not believe me that nothing has actually happened
2. A personal reference would be better
3. This saying depends on the situation, that is, on what happens to them.
4. Willingness to speak only moderately increased, because I already have nothing more to say.
5. inappropriate
6. Correct from the developmental psychological point of view, through the psychological and cognitive development (developmental stages and stages) it is assumed that the child is always in the weaker position and therefore the adult is responsible and accountable.
7. yes, but some victims think so anyway, I find such a statement invalidating, it is an attempt to control, to ascribe responsibility to oneself, as a victim you don't believe that anyway
8. can relieve, but a little overall
9. I know, but nothing happened to me in my role.
10. Could be even clearer: “...it is NEVER .....
11. Can at least also be used as a hook for telling more detailed circumstances, even if one rejects generalizations
12. This should be a matter of course.

7.56 Exploring unexpressed emotions and conflicts (EU01): “If you find it difficult to talk about it, tell me what is bothering you.”

7.56.1 Group “Abused”

1. The part of the sentence with the if is restrictive and unnecessary
2. somewhat unclear, as worries might be related to the experience?
3. It is good that “alternative topics” are also offered.
4. Gives me the feeling that he wants to understand me and doesn't want to force me to do something I don't want
5. okay
6. Before I can say anything, I first have to create a safe framework for myself, communicate my concerns and try to reduce them beforehand.
7. exactly that is often difficult, I think
8. Because it is not directly about the facts, but about the obstacles.
9. The official responds to my concerns. We can build and develop the conversation in peace, which relaxes the situation.

7.56.2 Group “Not Abused”

1. Access to information not directly, but rather slowly, which positively influences the willingness to speak and well-being
2. Strange request: it is difficult for you to tell, so tell it.
3. I find it too openly formulated, what do my worries have to do with the situation here, I understand it in such a way that the question is what the fear is, what could happen, whom I tell, but one could also think that I am allowed to tell about my everyday worries
4. Worries get space - my hand is extended
5. That's a nice question to ask, why I am so hesitant. Here I personally would not be afraid of a certain intention
6. The answers turn out like this because, as I said, I cannot confirm the accusations. I would have the feeling that something was being accused of me. I would be afraid of doing someone wrong with my statement. On the one hand I would not want to call possible victims liars, on the other hand I would not want to falsely accuse anyone. If I really could not confirm the accusations, I would feel very pressured by this question.
7. “If”- feels good. And telling what worries me, being invited to it, is also good for me.
8. I find good

7.57 Warmly emphasizing that the child is he key source to information (WI01): “I am asking you these questions because I was not there.”

7.57.1 Group “Abused”

1. reminds me that I was all alone and that I must remember completely correctly, creates pressure
2. has (read) an unpleasant undertone
3. That is obvious.
4. I get a declaration

5. I know that myself, exerts pressure
6. I think it's a good question because you are encouraged to tell more details than you might have done on your own.
7. Of course not, would be even nicer
8. A “stupid” statement in this context and not professional.
9. I only find this question useful if I have problems to answer in a way that the process is understood. Otherwise I would feel uncomfortable. It is clear that the policeman was not there. I would know that. And this sentence would perhaps make me doubt my own intelligence.
10. funny question
11. The victim is aware of the fact that the person was not there ... seems very unprofessional.
12. Acts reproachful and justifying. He is lucky that he did not have to be there at that time!

7.57.2 Group “Not Abused”

1. He should be glad not to have been there
2. Unsuccessful formulation
3. Stating the obvious, I think. But sure, can help if I would be very insecure at the beginning of the conversation. In the course of the conversation it would be a funny statement.
4. This is obvious and could therefore be interpreted negatively.
5. yes, I know that the person was not there. The sentence is somehow nonsensical.
6. Too indiscreet
7. one does not have to say the obvious
8. I don't need this explanation, maybe important for others
9. I personally think that this question shows empathy, and also that the policeman wants the truth and does not pursue any direct intention with his statements
10. Is it still possible?????!!!!! Would he have liked to have been, or what?????

7.58 Legitimating expressions (LE01): “You can talk to me about good and bad things.”

7.58.1 Group “Abused”

1. Are there good things?
2. Why would I want to tell anything other than what I came for. I understand that police officers are not supposed to suggest that something happened but the statement is strangely phrased.
3. Why does he think I have bad things to report?
4. What does he want?
5. neutral question/ neutral feeling
6. I'm an adult! I don't know her. I do not trust them
7. Gives me the feeling that I can decide for myself when to reveal bad things
8. that is meaningless.
9. The rating “good” or “bad” does not help me.
10. No coffee klatch.

7.58.2 Group “Not Abused”

1. No forcing the negative view, which has a positive effect on well-being and willingness to speak.
2. Why about good? I do not understand that.
3. not relevant statement
4. The rating is unpleasant and it is demanding
5. Here I would personally feel comfortable to say that nothing happened
6. Ok, if I want to help prove someone's innocence, then good things in the police force also make sense.
7. Can” in the beginning okay, also reassuring. Possibly emphasize later that it is important to tell everything relevant (good and negative)
8. Very meaningless.

7.59 Legitimating expressions (LE02): “In this office you can really say anything.”

7.59.1 Group “Abused”

1. Office is cold and impersonal. Better: They can tell me everything - personal relationship strengthened
2. Not a convincing statement.
3. “and no one will condemn them for it” would be a good addition.
4. ..say everything you feel ready for
5. polemic
6. totally arrogant, why should I believe that?, that scares me
7. Clarifies the “safe space”, I think is good.
8. Why? Can't anybody hear us here? Am I in danger (again)?
9. Would feel like I'm being talked into something
10. Very clumsy statement and not inspiring confidence.
11. The word office does not necessarily promote well-being.
12. Of course, I have to make sure that I don't have the feeling that someone might suddenly come into the office or overhear the conversation from outside.
13. good encouragement
14. on the one hand nice, because you have nothing to be ashamed of, but could also increase the pressure of expectations
15. Nonsense, it has consequences to make a statement to the police.
16. Creates an atmosphere of trust; feeling of “among us

7.59.2 Group “Not Abused”

1. Why only in this office ?
2. Would rather have the opposite effect on me and make me suspicious.
3. not suitable in the situation
4. nice, so also that the alleged perpetrator has not done anything to me :)
5. Too sweeping - unbelievable
6. I do not like chumminess at all

7. Personally, I would feel the pressure to say something that the policeman expects, but at the same time I would feel a little more comfortable
8. I would like the general conditions to be clarified at the beginning and not to be thrown in between. Confidentiality is super important in this setting, here I feel like it is taken lightly.
9. How does he know what I can or cannot do

7.60 Expression of confidence/optimism (EO01): “I am sure you can describe it well.”

7.60.1 Group “Abused”

1. If the situation is a very long time ago and I have consciously and successfully repressed it over the last years, this statement is almost like an attack on my memory. I have consciously decided never to remember the situation exactly.
2. “it” referring to sexual violence? - then “I am sure” is too assaulting and builds up pressure
3. How does it know that? distrust
4. Would put me under a lot of pressure to tell everything completely
5. too high pressure of expectation on me
6. he suspects
7. Confidence makes talking easier.
8. Repeat.
9. I will not do anything wrong.

7.60.2 Group “Not Abused”

1. That sounds like the beginning of a conversation. This policeman doesn't even know me. I don't believe such a statement, or rather it sounds like an empty phrase to me.
2. Willingness to speak is linked to fear of saying the wrong thing
3. Positive reinforcement, encourages to talk about it.
4. But I probably cannot describe it well and I know that.
5. a little pressure makes it
6. I find the wording disconcerting
7. Maybe motivating for some, I find it rather “sucking up”.
8. I personally would feel that I could say what I want, as long as it was the truth
9. How would he know that? Pretentious!
10. One feels pressured to describe something well. Especially in such a situation, you might want to say something more neutral “<Everything you say helps us. If I should not understand something, I will ask again afterwards.”

7.61 Expression of confidence/optimism (EO02): “I think you can describe it well.”

7.61.1 Group “Abused”

1. How does my counterpart want to know that?
2. I feel taken seriously
3. How does the interviewer know this?
4. provides security and confirmation

5. Takes away my insecurity
6. I don't think so and I don't want to, I don't care what you think, your expectation triggers expectation pressure in me
7. Doubts?
8. Good reinforcement
9. It puts you under pressure to at least tell something.
10. This sentence builds up too much pressure.
11. how does he know that ?
12. Confidence in my ability.
13. That's nice if you think so. What does that tell me?
14. He trusts me, so I can also trust him. I may describe the facts of the case as I see fit.
15. It puts me under pressure to be really good at it.

7.61.2 Group “Not Abused”

1. I find positive agreement important in this context
2. Well-being is reduced and pressure of expectation increased, because people are forced and asked directly about what happened. Creates the feeling that I have to say what the official wants to hear from me.
3. too much pressure
4. Describe what specifically?
5. should mean, I have already described it or is this a request?
6. this also creates pressure to perform, what if I can't describe it well?
7. could sound encouraging, for me it rather triggers pressure to finally start and how does he know that?
8. This statement would personally encourage me to continue speaking
9. Affirmation helps to keep talking
10. Before or after I have told? If this is a praise - then it opens, if it is a request, then all protection mechanisms are activated

7.62 Expression of confidence/optimism (EO03): “I am sure you can tell me.”

7.62.1 Group “Abused”

1. Too high expectations.
2. How can someone be sure of something if they are not in the same situation?
3. Why is she so sure? Perhaps not the best formulation.
4. The tone in which this is said is very important.
5. can apply pressure, can be helpful
6. Hmm?
7. The high expectations would put me under pressure
8. The choice of words is unfavorable... he/she is sure, but I am not sure.
9. the question exerts pressure on me and does not exactly encourage my willingness to speak.
10. Do not make assumptions about what other people can or cannot do. Whoever has come to the interview obviously has the intention to talk about what happened.

11. The officer expects me to tell about an incident. He is urging me to make a statement.
12. “...and if I don't want to talk about it...? “

7.62.2 Group “Not Abused”

1. Demanding phrasing that challenges me to say something I might not want to say
2. This now comes after the request to look him in the eye, so I still find this statement a bit encroaching.
3. this puts pressure, what if I can't do it (for whatever reason)
4. Annoyance, pressure, I don't feel like it anymore
5. very manipulative and pressuring. Would think about ending the conversation now ...
6. It's about whether I'm sure.
7. Cross-border!!!! Pretentious, disrespectful, manipulative.
8. very general statement, evaluation depends on initial situation

7.63 Offering help (OH01): “I want to make it easier for you, how can I help you?”

7.63.1 Group “Abused”

1. I feel that my feelings are taken seriously.
2. Empathy relaxes the situation.
3. very good!
4. I feel perceived, this makes me more open and I am ready to tell more
5. I find a good question!
6. I want to finish, because I have nothing to say
7. I am being addressed and that is good
8. here I feel valued, he offers help
9. I could not answer that question.
10. Don't assume that it is possible, but ask, “Is there anything we can do to make the situation more pleasant for you? A cup of tea to “hold on”, the possibility to make a statement in writing, the respondent should be of the same gender as the interviewee in order to remove hurdles.
11. Searches for solutions.

7.63.2 Group “Not Abused”

1. Neutral formulation that shows that my well-being is important but at the same time not a demanding expression.
2. I believe that in a situation like this, which is very new to me and probably more familiar to the official, I would feel a bit overwhelmed if I had to know at the beginning of the conversation what would help me at that moment. If the person who is questioning me there seems open and trusting, then I would rely on him or her knowing how to make it easier for me as a respondent. If this question came up later in the conversation, I might find it more helpful.
3. mindful/caring
4. The “how” is disturbing. Maybe he can't do it at all. At the same time nice that he wants to - and again at the same time clearly, he expects something

7.64 Offering help (OH02): “Would it be easier for you if you wrote it down yourself?”

7.64.1 Group “Abused”

1. Control over the situation is pleasant
2. gives the person concerned more control over the situation
3. The feeling of having several options gives me security, sometimes it is easier to put traumatic experiences into words in writing than to formulate them orally
4. Deals with me
5. Yes, I often find it easier to write things than to say them. Good suggestion! I would not have dared to ask for it myself.
6. I find it very nice that I am given options for action and that I can participate in the decision making process
7. good proposal
8. here readiness to speak = readiness to write
9. Very good.
10. The officer tries to help me.

7.64.2 Group “Not Abused”

1. I find a very good instruction!
2. It is very kind of you to try to find alternatives that might make it easier for me to report on the topic
3. That sounds accommodating, but I don't think writing something down would meet my needs.
4. good proposal
5. with readiness to speak in this case I would mean “readiness to write
6. Writing it down is often much easier than saying it.
7. Alternative offer can relieve, here somebody thinks about making it easier for me
8. Here they pay attention to my feelings and try to accommodate them or to make sure that I feel comfortable
9. Again, I find it difficult to put myself in this situation, as I cannot confirm the accusations.
10. Offers alternative possibilities, which I like
11. Writing it down helps to feel in control
12. A simple offer that I can accept or decline.
13. To write down “even” against the very objective police protocol puts already under pressure, or can we not at all?
14. You notice that the policeman wants to make it easy for you

7.65 Offering help (OH03): “Start speaking calmly and I will help you by asking you questions. I am here to help.”

7.65.1 Group “Abused”

1. “To help YOU” I would find more personal.
2. I find it pleasant to be able to talk at my own pace and not have words put in my mouth
3. I get support

4. questions can trigger, I prefer to keep control
5. It gives a light structure and gives me an impulse to just talk away. I do not feel alone.
6. the first sentence was still ok, but “I am here to help” would make me feel insecure. Nobody asked for help.
7. exaggerated, very psychological-therapeutic question
8. very open --> without pressure, nevertheless offered help (well-being could still decrease because you don't know where to start)
9. not very compassionate, if not even arrogant.
10. Stop the repetitions, you'll feel really stupid.
11. The police officer shows interest so that it is easier to talk about the experiences.
12. Facilitates the entry

7.65.2 Group “Not Abused”

1. the first part of the statement is pleasant (the second less so) because it takes away the worry of having to explain everything perfectly and you now rely on Ann to hook the interviewer on the important points.
2. Clear statement, I think.
3. Good feeling
4. Very good
5. instead of “2x help” the word only 1x support would be cheaper, I don't want to be that helpless
6. what can I say, I don't know anything. I don't need to be helped! Does he think I was abused, too? What's the point?
7. I personally like this question, because it is not so demanding but empathic. This would make me feel more comfortable.
8. Helping you speak?
9. So if I don't have a real concern of my own, I don't tell them anything! Start to speak. As if I was an actress and he was the cameraman, or what?
10. good introduction
11. The last movement creates a certain expectation of a negative experience, before which one should tell.

7.66 Encouraging non-verbal communication (EN01): “Go ahead and look me in the eye, so I can look you in the eye.”

7.66.1 Group “Abused”

1. I am being tested! he wants to see if I am lying
2. Too intimate.
3. Inappropriate request.
4. If the person feels more comfortable not to look the other person in the eye, this should be accepted in any case and no request should be made to do so differently.
5. This would overstrain me in the situation and I would feel uncomfortable. If necessary, I would say instead, “Could you maybe try to look at me while they continue to talk?

6. Maybe the person doesn't want you to look into their eyes while they remember something that hurts.
7. Huh, why does he want to look me in the eye all the time. Doesn't he believe me?!
8. this limits me enormously in my freedom to talk about it
9. could be perceived as very unpleasant
10. Why???
11. sounds a bit suggestive
12. I almost feel compelled. Extremely strangely formulated. UNPROFESSIONAL
13. I don't like to make eye contact - especially not with such personal and difficult topics. I would answer everything only them as fast as possible, so that I do not have to look further into the eyes.
14. But I do not want
15. It's hard to talk about something like this and look at someone while doing so. It is easier to look away.
16. It would be very uncomfortable for me to have to keep the eye contact compulsively. I might think that he distrusts me and wants to check my statements for credibility.
17. Extremely inappropriate and inappropriate in this context.
18. When I talk about difficult topics, I feel incredibly uncomfortable when I look at someone. For me it is much more comfortable to look at my fingers/flowers in the room/wall behind the other person. When I look into someone's eyes, it's easier for me to feel intimidated and I'm less good at them. Which should be difficult in this situation anyway.
19. what kind of question is that ? Comes across as strange
20. This seems very threatening, not very empathetic to a victim of sexual abuse.
21. Not conducive at all, very difficult content - I wouldn't want to look him in the eye and certainly wouldn't want to calmly tell emotional traumatic experiences.
22. Feeling defenselessness.
23. Why do you want to keep someone necessary eye contact in a conversation where you are incredibly vulnerable and want to hide away? Maybe this is even something the victim has heard from his tormentor! Be more sensitive!
24. Why does he want to look into my eyes? It seems oppressive. Doesn't he believe me? I don't want him to.

7.66.2 Group “Not Abused”

1. By the remark to want to look me in the eyes, one gets the feeling to be examined, I feel the behavior also as somewhat encroaching too intimate for a so shame loaded situation
2. Very critical statement. I feel too insensitive.
3. Extremely violent
4. Direct confrontation intimidates me and increases the pressure of expectation.
5. Very inappropriate question
6. I am an adult and I want to decide for myself when I look someone in the eye.
7. too intrusive
8. possibly a too big demand - instead “I would like to be able to look you in the eye”.

9. I don't like to be told how to do things. If I can't look him in the eye on such a difficult subject, then I won't do it
10. if I am ashamed of something, then I don't want to have to look into the eyes of the person opposite
11. too strong confrontation
12. Annoyance. I'll look at her if I want to!
13. I feel interrogated, portrayed as the guilty party and again “impaled on the policeman's gaze with no way out”. They contradictory formulations, first seemingly nice, then brutal. My trust is gone after the sentence!
14. Personally, I would not know whether I would (want to) have the conversation after such a statement
15. Very demanding, therefore somewhat unpleasant.
16. One feels as if he wants to check whether I am lying when I tell the story
17. Very uncomfortable, as if person would want to find out if I am lying, assault
18. Disgusting. Telling me where to look and expecting it to be okay with me when someone wants to look me in the eye the whole time!
19. looks funny
20. looking at it does not have to be, makes skeptical
21. It sounds very requesting and rude, straight with loading experiences one does not want to have necessarily direct eye contact.
22. Why should I look into your eyes? You think I'm lying?

7.67 Encouraging non-verbal communication (EN02): “[Your name], go ahead and sit next to me.”

7.67.1 Group “Abused”

1. Why beside it?
2. find the “sit next to the person” offer with the background of the story possibly too encroaching, but it depends on the situation and the policeman
3. For someone who has already been sexually harassed himself, he certainly does not seek closeness to a stranger in such a situation.
4. If you sit down next to me, this can be understood as an invitation, which probably does not trigger good feelings in victims of abuse.
5. the one with the sitting next to me, I find very inappropriate
6. physical. Proximity inappropriate
7. I would find it very critical of a male policeman, especially in cases of sexual assault.
8. also somewhat suggestive
9. It's not possible. As an abuse victim, I certainly don't want to sit next to a man I don't know and talk about how another man abused me. Absolutely crossing the border and disregarding any professionalism!
10. I want to be asked and not instructed
11. That's not possible!
12. cross-border

13. I may not want to, assault, caution
14. I don't want human proximity (especially not to “strangers”) in such a topic.
15. Never!
16. “sit down next to me” sounds more like a request than an option. That would put me under more pressure
17. Offering proximity is probably rather out of place for an abuse victim.
18. The worst panic.... is when it comes to the topic of physical closeness of a stranger - what helps?
19. Maintaining the distance!
20. Placing yourself next to a stranger - especially if you have experience of sexual violence and unwanted close contact - could make you feel uncomfortable, tense and unwilling to talk.
21. what's the point? This is not possible.
22. I would like to be allowed to decide for myself where I sit.
23. Why sit next to him? It is actually not relevant where I sit.
24. physically possibly too close
25. That would be too close and possibly encroaching.
26. This is creepy and not helpful to calm abuse victims.
27. Why should I sit next to him now? It's too close... It's uncomfortable.
28. The police officer is a stranger to me, I would like to keep a (certain) distance.

7.67.2 Group “Not Abused”

1. it is an unknown policeman I do not want to be close to
2. I would feel like a fool and like a little child.
3. Very unfavorable, especially when talking to victims of sexual abuse.
4. I would like to keep a certain formal distance from the official.
5. Sounds unsuitable
6. I prefer to sit with a little distance. I don't even know this man.
7. In the event that sexual harassment has taken place in advance, the request for an approach is frightening.
8. irritating, why should I sit next to it? i want to decide for myself how i sit, it might be better to ask at the beginning if you want to sit opposite or next to it
9. Sitting opposite is better.
10. too much proximity
11. Maybe still on the lap???
12. Entering my protection zone
13. This statement would be too demanding for me personally
14. I find this request somewhat border-crossing.
15. I find that funny, why should I sit down next to him in the middle of the conversation...
16. This feels intrusive and encroaching!
17. encroaching
18. Disgusting performance! Extremely abusive!!! Does something like this REALLY exist with the police? I really hope that if anybody knows about a policeman who behaves like this, he/she will step in and make sure that this person loses his/her job! That is not possible at all!!!!

19. It seems inappropriate to ask to sit (close) next to someone (hierarchy) when sex. allegations where a similar power differential may have prevailed
20. It is unpleasant to get too close to strangers.
21. Placing it next to it does not fit into the professional context, rather false-triggering.

7.68 Encouraging disclosure (ED01): "It is very important that you tell me if something has happened to you."

7.68.1 Group "Abused"

1. Say for whom this has great importance. Better: it might help you, if something happened, to talk to me about it, so that you can free yourself from it
2. Pressure build-up
3. the explanation is missing and what happens then
4. Acts like an interrogation
5. He already knows
6. You can certainly write even more complicated.
7. that is more objective
8. I felt a greater sense of pressure and paid less attention to my own needs.
9. It would have no meaning for me if I did not want to deal with events anymore.
10. How many times have we had this now? 5x?

7.68.2 Group "Not Abused"

1. despite the fact that nothing happened to me is a good/ pleasant formulation
2. Shows the sense to dare to tell about it.
3. It is not assumed that anything has happened to me, only that it could possibly be the case.
4. Is unpleasant, but an important question
5. the "if" is very important here!
6. Too sweeping, what do I do when I tell something, builds up pressure
7. Personally, I would feel here that I could say everything and that everything I would say would be okay
8. "if" takes some pressure of the "big meaning".
9. For whom please? And why? Without further explanation, it only adds pressure.
10. Find it quite okay, non-suggestive, motivating.
11. Isn't it also important if I can state that nothing happened to me?

7.69 Encouraging disclosure (ED02): "[Your name], if there is anything you want to tell me, I want to know/listen to you. It is important that I know this/that I listen to you."

7.69.1 Group "Abused"

1. I would like to listen to them is very pleasant, shows interest in me
2. "I want to..." sounds very demanding. "I would like..." is more appropriate.
3. "It is important that..." seems a little uncomfortable to me and reminds me that this is just his or her job

4. Better to listen than to know
5. “It's important that I listen to you.” ...is a prerequisite and should not be pronounced. Better: “If there is anything you want to say to me, you can do so here with peace of mind.”
6. Gives me the feeling that my statement is important, although formulated very bumpy
7. I find there is a significant difference between knowing this or listening to you, my answers would be different for each formulation.
8. Knowledge is not possible - pressure!
9. I find “listening” better than “knowing”. That is more personal.
10. listening to you the feeling is more pleasant than I would like to know
11. By emphasizing the importance, I would feel pressured to actually tell something.
12. “... it is important... “ automatically increases the pressure and I would switch to draft
13. A study has shown that the use of the word “because” is more likely to produce results. If the question is already insignificant, one can still build that in.
14. The version with “I listen to you” is much more friendly/relaxing than “I know that”.
15. empathetic
16. I feel taken seriously in this question and strengthened in my independence and not pushed to anything.
17. too demanding
18. There is less pressure to listen to the offer than to “want to know
19. Do not treat adults like children or as if they were not fully sane. Of course, the officer is there to ask questions and listen to the person who answers, after all, that is his job.
20. second alternative with listening

7.69.2 Group “Not Abused”

1. in the case of actual abuse, this formulation could be helpful, but in “my” situation without previous abuse, it would rather prevent me from telling further things and make me skeptical about the interviewer.
2. “Listening” is more appropriately phrased for me.
3. Knowing and listening is too different, I cannot make a statement.
4. invitation to report and that it is important to listen to me
5. I personally find the naming very rigid and formal, not very empathetic.
6. I can't answer this question like this, because they are two different ones. Variant A (“do I want to know this”, “it is important that I know this”) lowers my well-being a little, increases the pressure of expectation a little, does not change my willingness to speak. Variant B (“to listen to you”, “that I listen to you”) increases my well-being a little, lowers the pressure of expectation a little, does not change my willingness to speak. Variant A seems too demanding, variant B I find basically difficult. “I want to listen to you” sounds a bit half-hearted and also a bit hypocritical. “I want to listen to you” would be a statement in which I would feel taken much more seriously, because it sounds more specific and would rather inspire my confidence. This variant would moderately increase my sense of well-being, reduce the pressure of expectation somewhat and moderately increase my willingness to speak.
7. Question difficult to answer, that “knowing” and “listening” have an influence on others

8. The first half of the sentence loads, the second half nullifies this by the pressure it generates.
9. better with “know” in both cases

7.70 Encouraging disclosure (ED03): “Please continue and tell me more.”

7.70.1 Group “Abused”

1. This question is too vague & unsettles rather than promotes the willingness to speak.
2. objective and focused on topic, this is how he gets ahead with me
3. Unobjectionable question, rather small talk
4. I feel under pressure
5. too high expectations, it goes too fast, makes fear
6. The statement assumes that I have already told you something... so the situation seems to have become more positive.
7. How about responding to the interviewee, a short acknowledgement, praise or expression of interest? That is interesting, I understand, etc.
8. Objective
9. Has an intrusive and demanding effect.
10. “Tell me more”
11. Encourages me to keep on reporting.

7.70.2 Group “Not Abused”

1. By a friendly invitation or request, I would feel that there is a real interest in what I am saying. That alone would relax me in the situation.
2. what if there is no more?
3. Invitation to tell further/more
4. I would prefer questions of this kind, because they radiate a certain openness of results.
5. Very neutral statement, which does not include any particular expectation/suggestion.

7.71 Using the interviewee's name (UN01): To be addressed by name: “Thank you for coming, [your name].” or “If I say things wrong, please let me know, will you, [your name]?”

7.71.1 Group “Abused”

1. Does not seem authentic in some sentences
2. Perceives me as a person.
3. individually different - maybe ask at the beginning what is perceived as more pleasant?
4. Using the name too much and too often is reminiscent of freshly trained call center agents and has a rather negative effect. Just mentioning the name once is enough. I find it positive in the first sentence. Not in the second sentence.
5. It depends on whether my first or last name is used. first name: unprofessional; last name: I feel personally taken seriously
6. The feeling for me is taken into consideration
7. Wrong and right

8. It makes the situation more individual and I don't have the feeling of being just one of many, but that someone is really interested in me.
9. I would not care if my name is mentioned or not.
10. “Or-questions” cannot be answered here (there is only one possible answer)
11. It is important to be addressed by name, as it sometimes does not sound like a sensitive use of language. I find this question stupid, it would be better to start with the name.
12. I assume that I am addressed by my last name. This makes such a conversation more formal and impersonal for me and creates more distance than if I am not addressed by name.
13. it's a police investigation, the sentence is a bit strange
14. Perhaps the naming is too intense, urgent and personal - hard to say.
15. are two different statements
16. I keep the control.
17. The use of the name is generally beneficial, but if it is used excessively, one quickly feels manipulated because it seems so artificial.
18. Being addressed by name creates distance between him and me. The official seems too objective.

7.71.2 Group “Not Abused”

1. I would leave out the last name.
2. To be addressed directly by name is a bit unpleasant.
3. I love to be addressed by name.
4. Being addressed by name creates proximity
5. I find a salesman thing. It is unsympathetic to me if it happens too often
6. Seems more personal, attentive
7. Being addressed by name increases the pressure for me personally and gives me a feeling of discomfort
8. I like to be addressed by name
9. What kind of “things” should be meant? Doesn't sound very trustworthy. At least I have the encouragement to correct it.
10. can seem nice, attentive, but also become too much

7.72 Final comments on all techniques at the end of the survey

7.72.1 Group “Abused”

1. In my opinion, my well-being is strongly based on the expectations of the other person. So if the expectations of me increase, my well-being decreases because I could do something wrong. My willingness to speak is then accordingly directed at my well-being, if I feel bad, I probably don't want to talk about a situation I feel so bad about.
2. I felt very uncomfortable with the statement that you should look the policeman in the eye. I would be less willing to reveal information and was irritated.
3. Words like “should” and “must” force an oppressive or intimidating feeling in most statements/questions; they should be avoided.
4. I found the questions with looking into the eyes or sitting next to the interviewer strange in connection with sexual abuse, felt strange.

5. Without a real situation it is very difficult to answer the questions. For me it would be decisive how the questions are asked and how the person asking the questions generally affects me.
6. The large number of questions made it difficult to put oneself in the situation throughout the questionnaire
7. Some remarks were far too confidential for me. I assume that I have a possibly longer questioning in front of me, about a bad event that happened a long time ago. I would like to get this over with and I don't want to be “got to know well”. The most helpful things were a good door opener, factual questions about the topic, questions about how the situation could be made easier for me (sitting comfortably, taking a break, etc.).
8. I find it difficult to assess individual questions that are not included in the context.
9. Most of the questions/statements were extremely unprofessionally formulated and in my opinion exceeded some limits. For such a questioning one comes to the presidency to make unpleasant but necessary statements. There statements that one would like to get to know me better have no loss. Furthermore, the policeman does not serve as my personal therapist, but as the authority that convicts the teacher and brings him to account. I would also prefer to be questioned by a female police officer. If you are already talking about such an unpleasant topic, it is much easier for me personally if this happens in a pleasant but professional atmosphere. I find it unpleasant to be addressed with my first name. Many questions were formulated very invasively and made me feel very uncomfortable.
10. I had no sexual harassment I could not free myself from, so I was able to do the interview well. Questions that were too confidential made me feel uncomfortable. It quickly felt abusive and I had the feeling that I was experiencing something similar again.
11. Few questions were really appropriate for the described situation in
12. Question 79 cannot be answered in this way. If I hadn't only been asked the questions that are supposed to be positively supportive, I would be much better off.
13. I don't think I understood the aspect “pressure of expectation” very well. Or maybe the aspect did not fit very well for me in these questions. It is perhaps important to mention that I generally have a very low willingness to speak (no matter what the subject is) and that I often answer as I expect, what the other person wants to hear from me. And I don't like social interactions in general, especially when it comes to very personal topics. In short: I am extremely introverted :D
14. If I have sanded this up correctly, is this for teacher training?! Why then should I imagine a conversation with an unknown policeman/policewoman, and not with a teacher? I would have judged things very differently, partly because I am less sympathetic to policemen and ask myself how they can really help me. Because a sexual abuse trial is hard. Often it's statement against statement and evidence is difficult, especially after a few years.
15. The survey took much longer and therefore frustrated at some point. Unfortunately the statements/questions were not very empathetic, what one would expect in such situations and often too general.
16. The questions seem too technical and cold to me. In many parts the emotional level is missing. Many questions are also written rather than spoken language. The language must also be adapted to the respondent (age, education, origin, etc.).
17. In general, I would take care that sentences that are intended to convey trust or well-being to the victim with the same expression, such as “I am listening to you”, “this is my job” or “I

understand you" are not used too often, because at some point it no longer inspires confidence but becomes eerie. Nor would I go too much into the fact that I often talk to people who confide in me, because it might seem so, as if I were to compare people. So, me as the person interviewing and not me as the person interviewed.

18. Some of the questions seem too "psychological" for a police investigation. Maybe I have a different idea. In any case, the questions must be asked sensitively, since the respondents will certainly have partly terrible memories of events. This requires specially trained persons.
19. often had difficulties, because I liked the questions themselves, but the idea of a police officer (in uniform?) was uncomfortable for me
20. The questions were very mixed, I would have liked the questions to have been arranged more according to the beginning and end of the conversation.
21. In such a situation with a policeman asking these questions, I can imagine that I would be annoyed. I do not believe that people affected by sexual violence believe the policeman who so often expresses his understanding. It seems like a cliche because of the frequency. And too much "thank you" is also funny.
22. I believe that willingness to talk could often be associated with well-being Some of the questions I asked were of a general nature It was not stressful for me, as I have no personal experience
23. I would not speak so openly to a policeman directly if I did not have the direct intention of disclosing events. To be honest, it would be more credible for me if a policeman would express himself in a friendly and empathetic way, but I would not buy statements like "he cares about me".
24. I was in a neutral, curious mood before the interview. Now I am annoyed. One has the constant feeling of being taken for stupid. The questions may be more suitable for children but as an adult you feel like a fool. Especially young adults are very sensitive and close down when they have the impression not to be taken seriously. With regard to further training for teachers you should keep this in mind. Children and young people to those bad things happened, had to become usually very fast adult and take over responsibility, since nobody else did it for them. The last thing you need in this situation are people who treat you from above.
25. The study looks very promising and could provide a good starting point for future teachers. It should be noted that the survey of children and adolescents is very different from a survey of adults. The interviewer needs a high degree of empathy and a good feeling for the other person. Especially children are easy to influence. Therefore, suggestive questions should be avoided at all costs! If there are first indications of suspicion of sexual abuse of a child, the police should be informed immediately, who can take further measures. Furthermore, too frequent questioning of the child could lead to further traumatization and secondary victimization.

7.72.2 Group "Not Abused"

1. Some questions lacked context: if the teacher did not sexually harass me as a child, why would I start crying in conversation? Then I could not put myself in the situation so well.
2. I found the "it's my job" remarks mostly rather annoying and unnecessary, because other formulations already (and better) expressed this and actually everybody knows what investigators' job is, especially after they have already been invited to a meeting and accordingly

have already contacted by phone / mail etc. Basically, I could imagine that some of the questions might be received differently by an abuse victim and that they might be asked as the conversation progresses, if the suspicion of abuse is confirmed.

3. I found positive reinforcements va. helpful (e.g. I can understand your statement, it is not easy to talk about it)
4. Very accurate, detailed and apparently well thought-out survey!
5. It took almost an hour to fill out this survey. Maybe the time could be a bit more vague. E.g. “The survey takes about 30min, but can be a bit longer if you answer in detail. It was not a burden for me, because the official I was supposed to imagine asked me such pleasant questions. Had this fictitious person been insensitive, it would probably have been unpleasant for me.
6. There were quite a few questions. In terms of content, it was often difficult to relate them to the activities of the police. Without any previous professional knowledge I would certainly have assessed the effect of some questions/remarks differently.
7. Range of questions well designed. It becomes clear what is at stake. Perhaps one or two additional questions could be included, which are more extreme (regarding the content of the question and the social-emotional competences towards the interviewed person). This could lead to an even more precise delimitation of which questions are well posed from one's own point of view and which are not. In general, however, the questions were well balanced and one could put oneself in the situation.
8. Very interesting study! Would like to know more about it!
9. too many items, in the end I had little motivation left
10. I find it very funny to tell the policeman what I generally like to do. I understand that this should help to build up trust. Nevertheless, it seems in the wrong place. I wouldn't ask the policeman about his hobbies either.
11. The study was quite long and I commented a lot, so I think my well-being is limited precisely because it took so long.
12. I found it helpful to thank and praise the interviewee; I find it rather strange to want to get to know someone better or to learn something about him/her; I found all questions purely about worries and the topic more appropriate
13. It was astonishingly exhausting and tiring.
14. The topic was not a burden. The questioning took too long for me and some questions annoyed me. These were mainly questions that were either very emotional, assaulting (sit down next to me, look at my face ...) or showed arrogance (I know what you want to tell me ...)< Positive I found appreciative, caring and open questions and remarks that give me room for maneuver - the feeling of being able to help shape the situation, not just a means to an end (used again?)
15. I find some questions for teachers who speak with children/young people useful and appropriate, but in the described situation of being questioned by the police it is rather inappropriate. And the frequent requests to talk about what happened, about my secret and bad things, if nothing happened to me and I have already told the person asking about it, naturally seems to put pressure on me, although most of the questions could be answered well individually, if nothing happened to me.
16. I personally found the study to be very long, I needed a 2 minute break in between.

17. In general, I think it is more pleasant for me personally when I have the feeling of being “part of the team”. So I want to have the feeling that I have a piece of the puzzle that needs to be solved and my counterpart and I work together to find and dig out that piece of the puzzle. I want my counterpart to meet me at eye level and to be my colleague. I hope it was somehow understandable what I mean?
18. I found it fundamentally difficult to imagine a scenario on the one hand where I could not confirm the accusations and then imagine, however, having said things that did not fit m.M.n. Accordingly, I found it incriminating to be suggested/imputed that something had happened to me after all. In general, I find it difficult to generate empathy through standardized guidelines, since every person is an individual case. Although these guidelines could serve as an aid for inexperienced interviewers, empathy is an inner attitude and a matter of practice and experience. It seems to me to be most important that the interviewee recognizes that the interviewer's interest is sincere and open-ended and that the interviewee feels that he or she is taken seriously. If questions are asked in a clichéd manner and the interest is not sincere and open-ended, the interviewee will very quickly notice this.
19. If this is the way police interrogations are conducted, then I am surprised that perpetrators are punished at all. My greatest respect to all who get through this. It makes me horribly angry and shakes me deeply. Where to put this anger? If this is really the system that is supposed to protect us and provide for our rights- then this is another impotent rage. Then we are still sooo in the beginning, soooo far from justice, it is terrible. And what does this have to do with teachers? The whole time it was about police officers. Where is the connection, please?
20. Some questions are difficult to answer if you assume a situation without sex. assaults. they have a pathologizing effect? I also found it very clichéd (especially as a male teacher at an elementary school) that in the fictional example it must be a male teacher. (a homosexual. Colleagues became sex. abuse on children. A lot of press. In retrospect the accusation could not be confirmed, but the mother of the child has a problem with homosexuality)
21. In my opinion, everyday phrases such as “I'm here for you” or “I understand you” are not conducive to communication.
22. Except for a few items (sit with me, look at me) the items were quite good. The focus on the personal interest in getting to know me, being grateful, knowing me better, etc. all seemed strange to me.
23. The questionnaire is clearly too long. Also, the control questions are sometimes very conspicuous (directly one after the other). Many statements make little sense in a police context or are very unrealistic.
24. Many of the questions presuppose that I, as an alleged victim who has never been sexually abused, am affected/afraid/rage. This gives me the impression that I should feel bad about the offense, even though I am actually neutral to it.
